# Diverse and complex male polymorphisms in *Odontolabis* stag beetles (Coleoptera: Lucanidae)

**DOI:** 10.1038/s41598-017-17115-5

**Published:** 2017-12-01

**Authors:** Keita Matsumoto, Robert J. Knell

**Affiliations:** 1 Department of Life Science, Natural History Museum, London, SW7 5BD United Kingdom; 2 School of Biological and Chemical Sciences, Queen Mary University of London, Mile End Road, London, E1 4NS United Kingdom

## Abstract

When male animals engage in intrasexual contests then any alternative tactics they use can be associated with dimorphisms in the expression of weapons. Some species have recently been found to exhibit trimorphism in their weaponry, suggesting that the processes leading to their evolution and maintenance of these polymorphisms can be more complex than previously thought. Here, we describe the extraordinary diversity of polymorphism within the genus Odontolabis: there are dimorphic species (*O. siva* and *O. platynota*), trimorphic species (*O. cuvera*, as previously described, and *O. sommeri s.stricto*) and, uniquely, tetramorphic species, with males of *O. sommeri lowei* and *O. brookeana* showing four clearly differentiated male morphs: small “Gammas”, “Alphas” which express large, long mandibles, “Betas” which have long mandibles with different morphology and “Boltcutters”, with short, wide mandibles. Such polymorphisms are usually thought of as being maintained as a status-dependent conditional strategy, but we found only one size threshold: in most cases males develop into Gamma males below a certain size but there is no relationship between morph and body size amongst the larger, ‘weaponised’ morphs. We suggest that the complex polymorphisms in these animals are probably maintained by a combination of a conditional strategy and a genetic polymorphism.

## Introduction

Intrasexual phenotypic dimorphisms in male animals are widespread and known often to be associated with the use of alternative tactics for acquiring mates^1,2^. Typically, there is a large or heavily armed “major” or “fighter” morph, which engages in aggressive interactions with other males in order to monopolise access to females, and a smaller or lightly armed “minor” or “scrambler” morph which either avoids such interactions or only occasionally engages in them, preferring to rely on either mobility or “sneak”tactics to acquire mates. Examples of these from the invertebrates include the mite *Sancassiana berlesei* ^3^, the dung beetles *Onthophagus acuminatus* ^4^ and *O. taurus* ^5^ and the earwig *Forficula auricularia* ^6^. Most of these dimorphisms are determined by the males in question following a conditional strategy^7–9^, as in the case of the dung beetles which develop into minor morphs if they are below a threshold body size, and into major morphs if they are above it^4,5^. There are also a few examples of genetically controlled male dimorphisms, for example in the damselfly *Mnais costalis* ^10^ and the cichlid fish *Lamprologus callipterus* ^11^. In addition to these dimorphic species there are also trimorphic examples, with males expressing one of three distinct morphologies. Unlike most dimorphisms, the best known of these trimorphisms are genetically controlled, such as those found in the Ruff, a wading bird (*Philomachus pugnax* ^12^), the side-blotched lizard *Uta stansburiana* ^13^ and the marine isopod *Paracerceis sculpta* ^14^. Rowland and Emlen^15^, however, described apparent condition-dependent trimorphism from several species of the scarab genus *Phanaeus*, from the lucanid *Odontolabis cuvera* and from the weevil *Parisoschoenus expositus*, with two body size thresholds determining which morph a male devleops into. More recent work has found similar trimorphisms in the lucanid *Dorcus rectus* ^16^, the weta *Hemideina crassidens* ^17^ and the harvestman *Pantopsalis cheliferoides* ^18^. In the latter case the harvestmen develop into a morph with small chelicerae below an apparent body size threshold, but above this threshold they can develop into one of two different morphs with different weapon morphologies, with morph being unrelated to body size.

Here, we investigate the diversity of polymorphisms in males of the stag beetle genus *Odontolabis* (family Lucanidae) in more detail. Male polymorphisms have been known from the Lucanidae for some time: apparent dimorphism in *Lucanus cervus* was first described in 1977^19^. Eberhard and Gutierrez^20^ described dimorphism in males of *Cyclommatus tarandus* and, again, *Lucanus cervus* in 1991, and dimorphism has since been described in a number of other lucanid species^21,22^. Most further research has concentrated on the different morphs of *L. cervus*. Two detailed studies of the allometric relationships of body parts in *L. cervus* have found that head width covaries with mandible length such that the ‘switchpoint’ between the morphs can be detected in measurements of the head as well as of the mandible, reflecting increasing muscle mass in the heads of the major males^23,24^. Hardersen *et al*.^25^ were able to use measurements of beetle remains left after predation to show that the size threshold for above which males tend to develop into majors is fluid and changes during a season, with larger beetles eclosing as minors later in the summer. Lagarde *et al*.^26^, on the basis of a study of a small number of animals, found that the larger ‘major’ males are more likely to initiate aggression than the small ‘minor’ males, and more likely to win contests. Romiti *et al*.^24^ also demonstrated, using a capture-mark-recapture study, that minor males appeared to have higher survival probabilities than major males, possibly as a consequence of differences in dispersal behaviour between the morphs.

On the basis of this limited set of information, we can cautiously infer that the dimorphism in *L. cervus* at least is likely to be similar to the well-described male dimorphism found in, for example, *Onthophagus taurus*. This species also exhibits a threshold body size below which males develop into minor morphs and above which they mature as horned major morphs, and minor beetles rely on ‘sneak’ tactics to acquire matings whereas majors engage in aggressive contests to gain access to and monopolise females^5^. Similarly, there do seem to be differences in aggression between the morphs of *L. cervus*, although the exact details of how the morphs differ are not currently clear - it is certainly possible, for example, that the minor males of *L. cervus* are relying more on mobility to acquire matings than on sneak matings with females who are already defended. In *O. taurus* the dimorphism is believed to arise because the beetles are following a conditional strategy arising from status-dependent selection, with small males gaining greater fitness benefits from following the sneak strategy and large males profiting more from following the aggressive strategy^27^. Whether similar status-dependent selection is behind the dimorphism in *L. cervus* is not known but it is, at least, a likely explanation.

Turning to male trimorphisms with different weapon morphologies, these have, to date, only been described from two species of lucanid and a small number of other invertebrates. We know little about how widespread these are, or whether the described trimorphisms map to discrete behavioural strategies in the same way that dimorphisms do, or how variable the nature of these trimorphisms are. Non-conditional genetically based trimorphisms known from animals such as the side-blotched lizard are often governed by the “rock-paper-scissors” game theory model^13^, and Rowland *et al*.^28^ extended the status-dependent environmental threshold model^8^ to cover the three-morph rock-paper-scissors scenario. This model predicts the existence of trimorphisms and thresholds consistent with observations from *Oxysternon* dung beetles, but whether the trimorphisms known in lucanids are also consistent with a two-threshold, three morph model is not currently clear.

There are, therefore, some important gaps in our knowledge of trimorphisms within the Lucanidae. In this study we examine the morphology of six species of *Odontolabis* using both linear measurements and geometric morphometrics, in order to determine 1) whether other species of *Odontolabis* also display trimorphism, whether they show different forms of polymorphism or whether they are monomorphic; 2) whether the relationship between morph and size indicates a threshold based morph determination mechanism and 3) whether the morphology of the head and prothorax covaries with morph in these species in a comparable manner to that described from *Lucanus cervus*.

## Results

### Morph allocation

**Figure 1:**
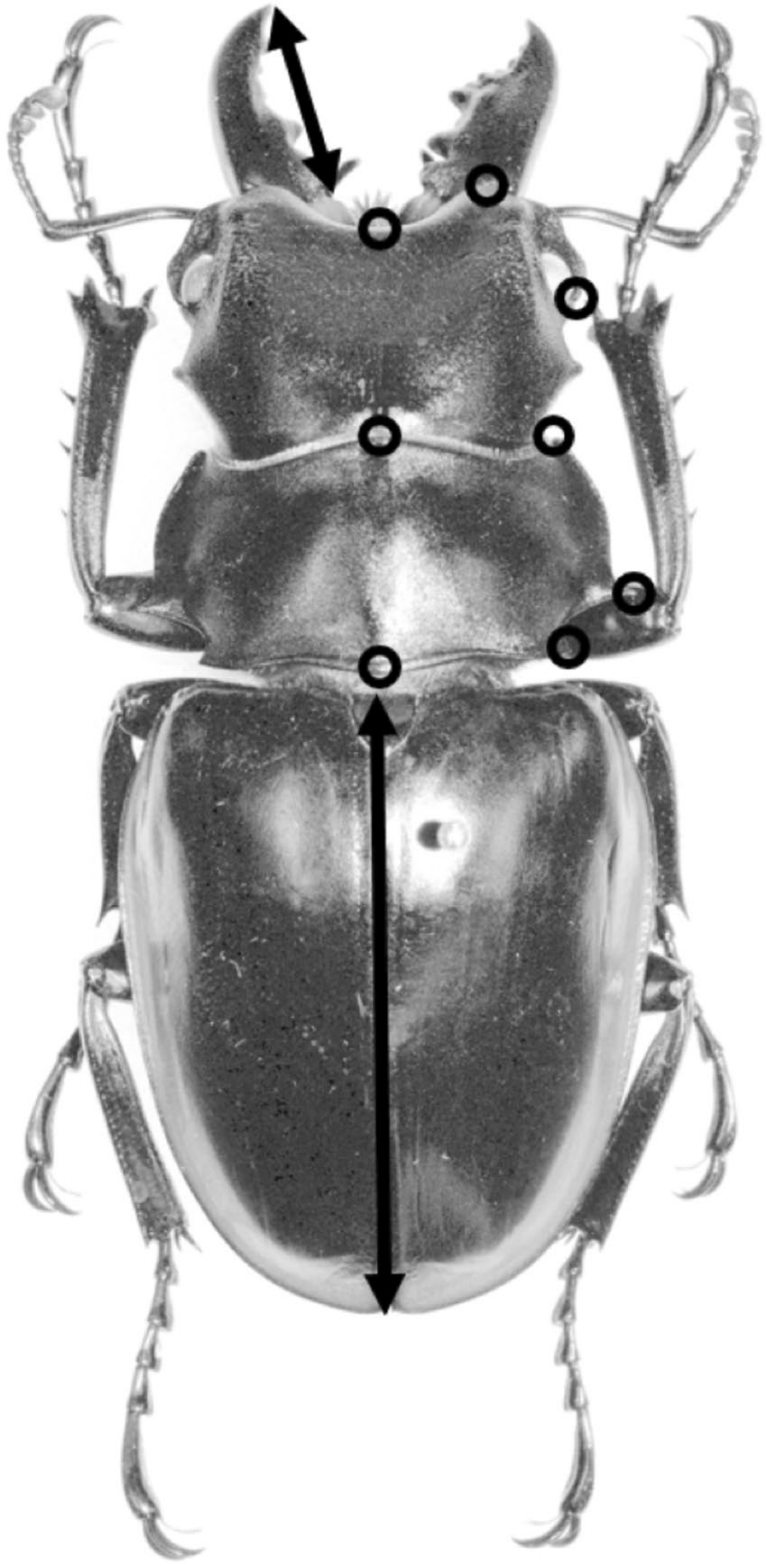
Measurements and landmarks. Measurements of left mandible and left elytron, and the location of the landmarks measured from all specimens. (Picture of *Odontolabis cuvera* from Natural History Museum of London).

**Figure 2:**
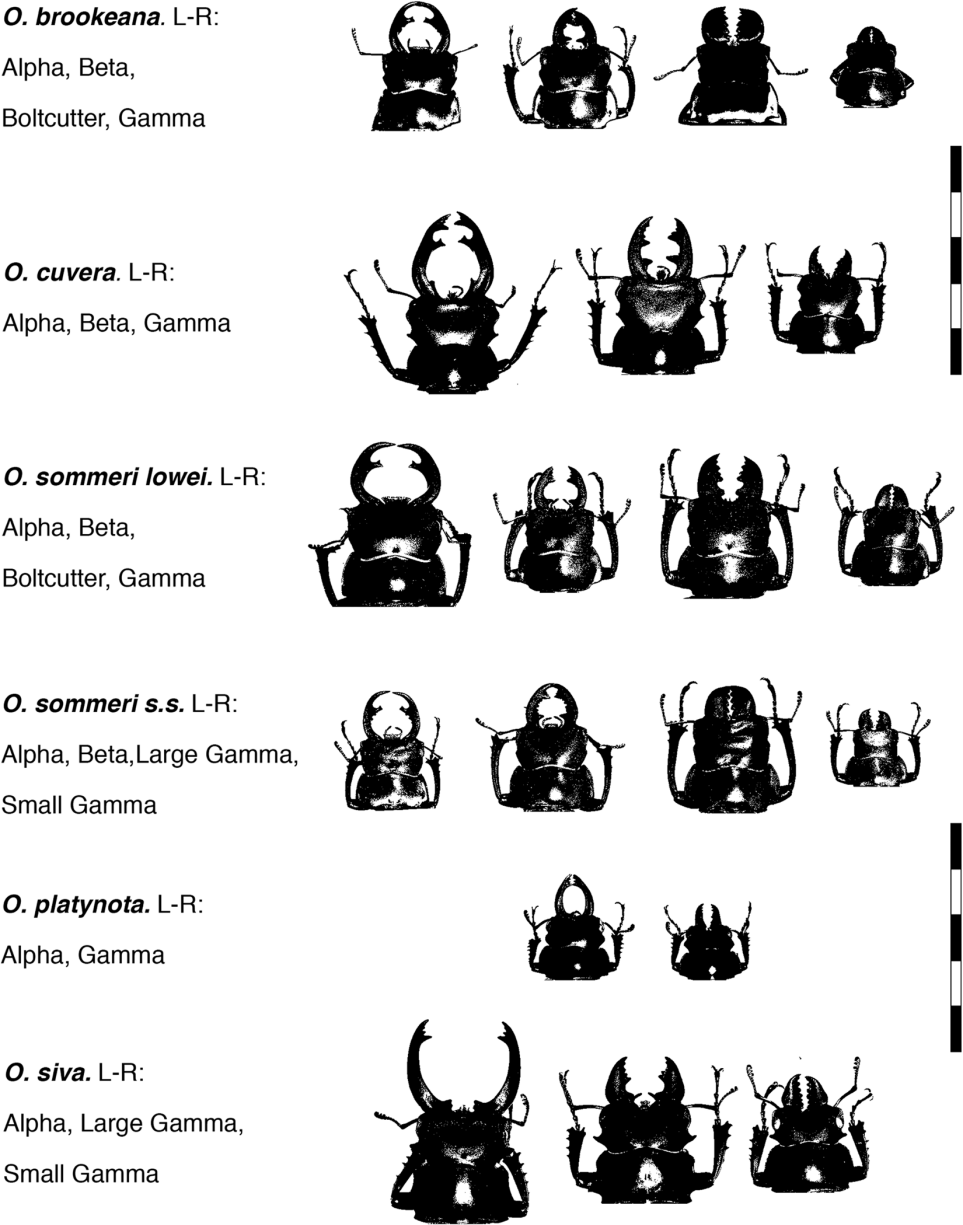
Examples of the different morphs from the six species studied here. Scale bar is in cm. Note that in a few cases mandibular palps or other parts have been digitally removed in order to make the shape of the mandibles clearer.

**Figure 3:**
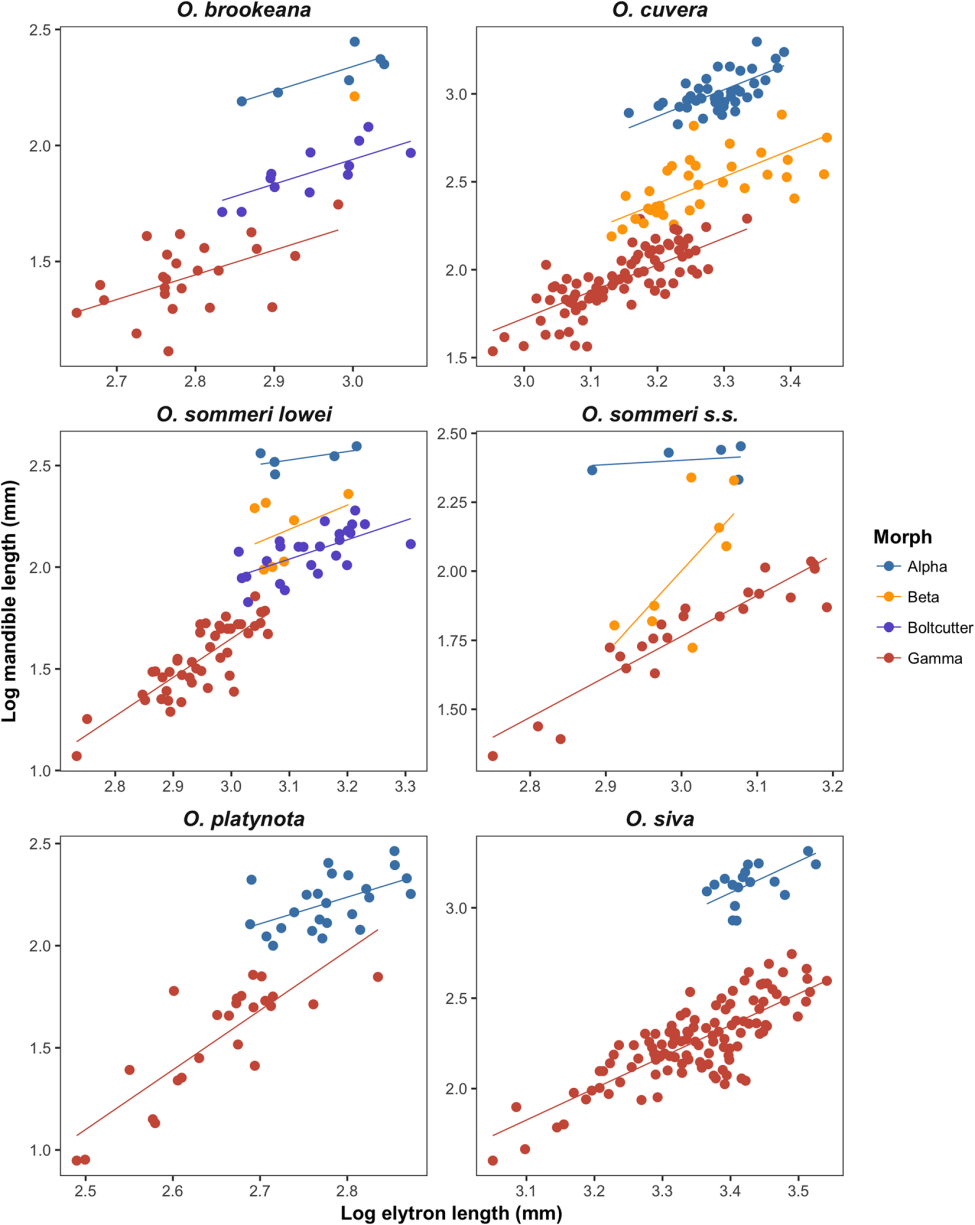
Allometric relationships between log mandible length and log elytron length (a proxy for body size) for all six species. Lines are fitted from statistical models describing the relationship between mandible length and elytron length selected on the basis of AIC. In some cases (e.g. *O. cuvera*) the model has a common slope for all morphs, in others (e.g. *O. sommeri lowei*) the slopes differ. Note that for *O. cuvera* and *O. siva* the common slope model and the different slopes model were not distinguishable on the basis of AIC scores because the difference in AIC was less than two. For these we show the model with the lowest AIC score overall.

Initial morph allocation was carried out using the methods described in^29^ on the basis of linear measurements of mandible length and elytra length (a proxy for body size: Fig. 1), as well as by visual inspection of the animals. The full analysis and all R code is available in the supplementary material. We found that the six species examined here express remarkably diverse male polymorphisms. Figure 2 shows examples of the various morphs from each species, and Fig. 3 shows the allometric relationships between body size (as indicated by elytron length) and mandible length, with lines indicating the predicted values from the best-fit models describing this relationship. Two species (*O. platynota* and *O. siva*) have dimorphic males, developing either into Alpha males with extended mandibles or Gamma males with small mandibles. *O. cuvera*, as has previously been described, has three male morphs, with Beta males developing mandibles with different morphologies from the Alpha males, and Gamma males which develop small mandibles. *O. sommeri lowei* and *O. brookeana* have four distinguishable male morphs: Alpha, Beta and Gamma males which resemble those described from *O. cuvera*, and a fourth morph of males with broad, robust mandibles which we call the Boltcutter morph. *O. sommeri s.stricto* had three identifiable morphs, Alpha, Beta and Gamma but a larger sample size might reveal a distinction between Boltcutter and Gamma males: the large Gamma males have large, robust mandibles but there was insufficient evidence of a qualitative difference with the smaller males to conclude that they are separate morphs. A full analysis of morphology and morph allocation is given in the online supplementary information.

**Figure 4:**
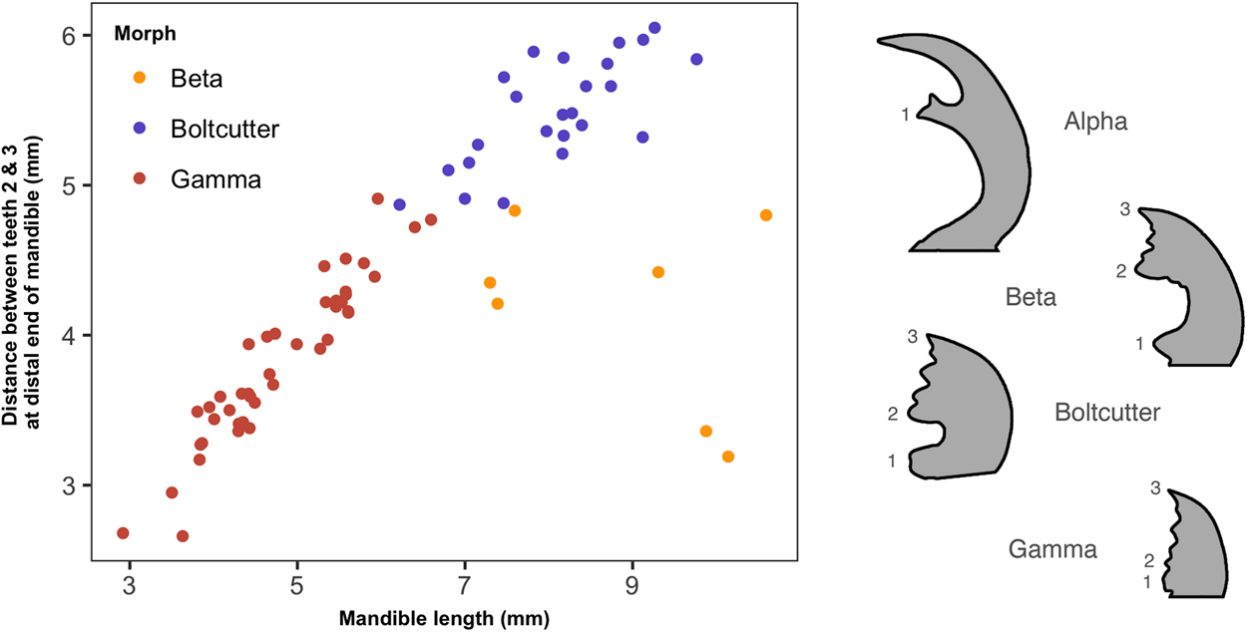
Further exploration of morph allocation in *O. sommeri lowei*. Left: the distance between the two most distal major teeth on the mandible (teeth 2 & 3 in^15^) plotted for Alpha, Beta and Boltcutter morphs of *O. sommeri lowei*. Alpha morphs are not plotted because they do not express these teeth - instead their mandibles end in a sharp point. Right: examples of the mandibles of the different morphs showing the location of the teeth.

To check whether the allocation of *O. sommeri lowei* to four separate morphs was justified, and in particular to check whether the division between the Beta and the Boltcutter morphs could be confirmed, we measured the distance between the two most distal major teeth on the mandibles, as was done by^15^ in the case of *O. cuvera*. Figure 4 shows the inter-tooth distance plotted against mandible length, and there are clear differences between Beta and Boltcutter males, with beta males having substantially smaller inter-tooth distances. The Gamma and Boltcutter males also group separately in this plot, further supporting the division into four morphs.

In both *O. brookeana* and *O. sommeri lowei* Alpha and Beta males are much rarer than in *O. cuvera* (see supplementary material). In the latter species they make up 26 and 21% respectively of the total sample, whereas in *O. brookeana* they contribute 14 and 2% respectively (there was only one Beta male represented in the *O. brookeana* sample) with Boltcutters making up 28% and Gammas 56%. In *O. sommeri lowei* Alphas and Betas make up 6 and 9% of the sample, with Boltcutters making up 29% and the remaining 56% being gammas.

No species seems to have more than one size threshold controlling their development into different morphs (Fig. 3). In *O. brookeana*, *O. cuvera, and O. sommeri lowei* there is a size threshold below which males tend to develop into Gammas and above which males tend to develop into one of the alternative morphs, but in none of these cases is there any suggestion of a further threshold separating (for example) Beta and Alpha males. It appears that when the animals are below a certain size they develop into Gamma males, but above this size their developmental pathway is not strongly influenced by body size: notably, in none of these species is the largest male an Alpha morph. In *O. sommeri s.s*. there does not appear to be such a size threshold: rather, males of all sizes develop into Gammas but only above a threshold size do they also develop into Beta or Alpha morphs.

Turning to the two species with dimorphic males, in *O. platynota* there does appear to be a size threshold above which most males develop into an Alpha morph. In *O. siva*, by contrast, Gamma males are found across the size distribution whereas Alphas are only found amongst the larger males, so in this species it appears that the size threshold is much more probabilistic. As with the tri- and tetramorphic species, the largest male of *O. siva* is not an Alpha morph.

### Morphometric analysis

**Figure 5:**
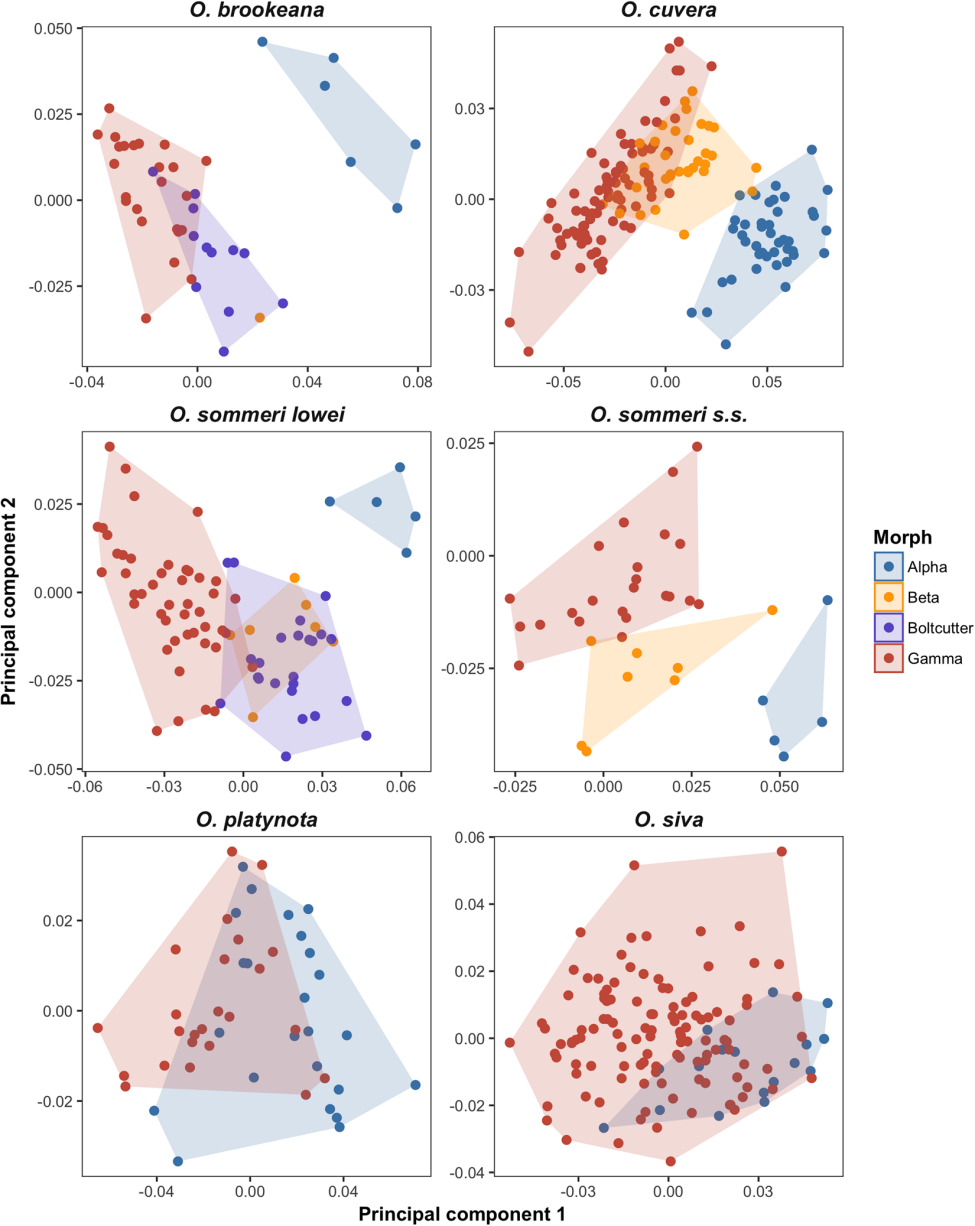
Relative warp analyses for the six species studied here. Shaded areas indicate convex hulls for each morph.

Figure 5 shows plots of the first and second principal components arising from relative warp analysis of the morphometric data. In all of the tri- and tetra-morphic species the Alpha males cluster separately from the other morphs, and in the two tetramorphic species the Boltcutter males and the Beta males are found together (although there is only one Beta male for *O. brookeana*), and show a reasonable amount of separation from the Gamma morphs. The two trimorphic species differ in how the Beta and Gamma males cluster, with Betas largely clustering with Gammas in *O. cuvera* but not in *O. sommeri s.s*. These differences between morphs are retained when body size is included in the analysis as well (see analysis in the supplementary material).

**Figure 6:**
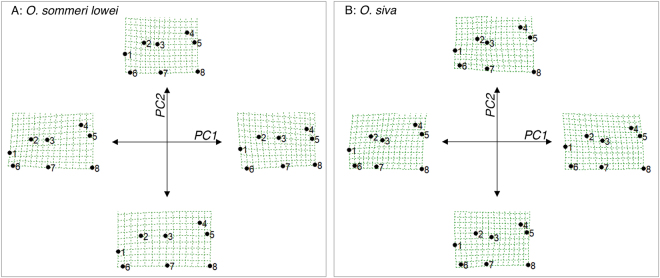
Example thin plate splines showing the changes in head and prothorax shape along PC1 and PC2. Left is *O. sommeri lowei* and right is *O. siva*.

On examination of thin-plate spline (TPS) plots (Fig. 6a, see also supplementary material) we found that all of these analyses lead to qualitatively similar changes in shape with PC1 and 2. PC1, which is associated with the majority of the separation between morphs, is mostly driven by changes in the relative size and shape of the head. Gamma morphs typically have low values of PC1 and typically have relatively narrow heads compared to their prothoraxes, with the anterior ends of the head being much narrower than the posterior ends. High values of PC1, as found in Alpha males, are associated with heads that are relatively wide compared to the prothorax, and with relatively wide anterior ends of the head. Negative values of PC2 are mostly associated with relatively long heads, with the increased length arising from an increased distance between the eye and the front of the prothorax: positive values of PC2 indicate relatively short heads.

For the two dimorphic species, the picture is rather different. In neither case is there clear separation between the Gamma and the Alpha morphs, and in *O. siva* the two convex hulls overlap almost completely, with many Gamma males having head shapes that do not differ from those of Alpha males. Furthermore, the TPS plots (Fig. 6b and supplementary material) indicate that although the kinds of change in shape associated with PC1 and PC2 are similar to those found in the tri- and tetramorphic species, the magnitudes of the changes in shape are rather less.

## Discussion

Within *Odontolabis* we find extraordinary diversity in the forms of male polymorphism, with different species having di-, tri- and, surprisingly, tetramorphic males. Even within species with the same number of morphs there is diversity in the way these morphs relate to body size and mandible size, with Gamma males developing at all sizes in the cases of *O. sommeri s.s*. and *O. siva*, whereas in the other species it is only the smaller males that develop the Gamma morphology. In total, then from our six species of *Odontolabis* we find five different kinds of polymorphism. These are 1) dimorphic (*O. platynota*); 2) dimorphic with Gamma males developing at all sizes (*O. siva*); 3) trimorphic (*O. cuvera*); 4) trimorphic with Gamma males developing at all sizes (*O. sommeri s.s*.) and 5) tetramorphic (*O. brookeana* and *O. sommeri lowei*). This diversity of polymorphisms is unprecedented within the animal kingdom: the only comparable example of which we know is the diversity of polymorphisms in *Phaneaus* ^15^, but these mono-, di- and trimorphic dung beetles all seem to have similar threshold mechanisms operating, whereas here we have diversity in the types of size threshold and the way that the animals develop into the various morphs.

The presence of tetramorphic males in two of these species is striking, and to our knowledge, this is the first time that any animal species with such a range of distinct male morphologies has been identified. Some species display highly polymorphic colouration of males arising from sexual selection (e.g. Guppies *Poecilia reticulata* ^30^; tree lizards *Urosaurus ornatus* ^31^) but to the best of our knowledge no examples with clear morphological differences between four male morphs have previously been described. Given the novelty of this finding, an obvious question is how confident we can be that these species really do have four male morphs. One potential source of error here is taxonomic: if some specimens are misidentified then we might find ourselves mistakenly assuming that a sample of specimens that are really from a mixture of species is from a single species. The specimens we used for this study are from a collection originally assembled by the noted lucanid taxonomist Hugues Bomans, and the curation and determination of the collection is of a high standard, so we think that misidentifications are unlikely. It is possible, of course, that we could have a cryptic species complex in the case of *O. sommeri lowei* or *O. brookeana* causing us to find tetramorphism in these animals, but we regard this as unlikely: we would have to posit a cryptic species complex with males that are identical in appearance aside from their mandibles, and which are all drawn from the same distribution of sizes. Since we have identified similar tetramorphism in two species we would have to have a similar species complex in both species. Overall, we consider the two species each with four morphs explanation to be more parsimonious here.

A second possibility, of course, is that we are incorrect in our conclusion that these animals can be divided into four morphs. In *O. sommeri lowei*, however, we can see clear divisions between the morphs on the basis of both qualitative differences in shape and differences in the allometric relationships between groups. The Boltcutter and Beta morphs do not appear especially well separated by the allometric plot shown in Fig. 3, but statistically a four morph model gives a considerably better description of these data than a three morph model with Boltcutters and Betas combined (see supplementary material for the full analysis). Furthermore, these morphs do show good separation morphologically on the basis of the distance between the second and third major teeth on the mandibles, as shown in Fig. 4. We therefore have confidence that *O. sommeri lowei* is indeed tetramorphic. We have somewhat less confidence in the same conclusion for *O. brookeana* because there is only one Beta male in the sample, but there is no particular reason to think that this should be regarded as an aberrant specimen rather than a single representative of a rare morph.

We have no information on the behaviour of male *Odontolabis*: there are no published reports of their behaviour that we can find, so we do not know how these beetles use their enlarged mandibles. The Gamma males certainly appear to be an unarmed morph which is not investing in weaponry, presumably because their fitness is maximised by following an alternative strategy: either attempting ‘sneak’ matings with females who are being guarded by other males, as in the case of *O. taurus* ^27^, or possibly by using higher mobility to reach unguarded females before other males, as has been suggested might be the case in some dimorphic Dynastidae^32^. This latter explanation is speculative and the extent by which animals such as *Odontolabis* might be impaired in flight by their weaponry is unclear: computational fluid dynamics modelling of the lucanid *Cyclommatus metallifer*, males of which carry exceptionally large mandibles, suggests that their weight should impose a significant energetic cost during flight, but empirical attempts to find flight costs associated with secondary sexual ornaments or weapons have not been especially successful^33^; indeed, in the Rhinoceros beetle *Trypoxylus dichotomus* the large and elaborate horns carried by large males have surprisingly small effects on flight performance because of their light weight and the slow flight speeds of these beetles^34^. Whether the mandibles of lucanids have similarly small effects on flight performance is unclear. The “weaponised” morphs presumably engage in contests over mating opportunities, and the differing shapes of the enlarged mandibles lead us to speculate that the different morphs use these in different ways during contests. The robust pincers carried by the Boltcutter individuals have the appearance of weapons which are adapted for cutting, and these could well be used directly to try to injure rivals. The longer mandibles carried by Alpha and Beta males are probably better adapted for wrestling and picking rivals up, as found in contests in *C. metallifer* ^35^. It’s notable, however, that in both subspecies of *O. sommeri* and in *O. brookeana* the mandibles of the Alpha males end in a sharp, inwardly facing point whereas the mandibles of the Beta males end in a wider array of minor teeth which would be more suited for gripping an opponent. In *O. cuvera* and the two dimorphic species the Alpha males’ mandibles are wider at the ends and better adapted for gripping. Is it possible that the sharp, dangerous looking mandibles of the Alphas in the tetramorphic species are adapted for a more risky and physically damaging form of combat which is necessary to counter the Boltcutter morphs?

The use of geometric morphometrics to investigate head and prothorax shapes adds to our understanding of these polymorphisms by showing that different mandible morphs are associated with different head and prothorax shapes: different morphs mostly have qualitatively different shapes for their heads and prothoraxes. The clear separation of the Alpha morphs in all four tri- and tetramorphic species, with their convex hulls not overlapping with those of the other morphs at all in most cases, indicates that these animals, in particular, are developing in very different ways from the remainder of the males. These differences are not simply determined by body size, with all big beetles having large, wide heads - analysis of the morphometric data with body size included as an explanatory variable (supplementary material) confirms the differences seen in the PCA plots. Distinct differences can also be seen between some other morphs, such as the differences between Boltcutter and Beta morphs on one hand, and Gamma morphs on the other in *O. sommeri lowei* and *O. brookeana*. The lack of such clear differentiation between morphs in the two dimorphic species is also notable.

Goyens *et al*.^36^ pointed out that the long mandibles of male lucanids will cause reductions in bite force because the long mandible means that the output lever in the system will be much longer than the input lever. By using micro-CT scans of the heads of male *Cyclommatus metallifer* they were able to show that these animals compensate for this by firstly developing a longer input lever - the distance between the mandible hinge and the attachment point for the mucles that closes the jaw - and secondly by growing larger closer muscles. These determine the shape of the head because a long input lever means that the anterior end of the head must be wider, and the attachment for the jaw closer muscles is at the rear of the head, so larger muscles will be associated with the posterior of the head being enlarged. This relationship between mandible size and head size is also supported by an interspecific comparison which found that species with large heads also have relatively large mandibles^37^. Given this, the clear difference found between morphs of the tri- and tetramorphic species in the geometric morphometric analyses makes sense. Alpha males have notably longer mandibles than all other morphs, and they have relatively wide, more rectangular heads, which is likely to reflect similar adaptations to those found by Goyens *et al*.^36^, with longer input levers and more investment in closer muscles to compensate for the loss of bite force associated with the longer mandibles. In the two tetramorphic species the Boltcutter morphs were also distinct from the Gamma morphs and again this largely reflects similar changes in the relative size and shape of their heads to the Alpha males, although not of the same magnitude. The Boltcutter males have much shorter mandibles than the Alpha males, and so will not require as much investment in either the input lever or the mandible closer muscle to achieve the same bite force as the Alpha males, and this appears to be reflected in the shape of their heads. It is quite possible that Gamma males simply avoid conflict, or only rarely engage in it: their small heads do not appear to carry large closer muscles, and the narrow anterior ends indicate that the input levers for their mandibles are not enlarged. It’s possible that these shape differences also point towards differences between *O. sommeri lowei* and *O. cuvera* in terms of the behaviour of the Beta morphs. In the former species the Beta males cluster with the Boltcutter males, indicating a similar degree of investment into mandible closer structures, but in *O. cuvera* the Beta males are mostly clustered with the larger Gamma males, suggesting either that the Beta males are not investing particularly strongly in apparatus to close their mandibles, which might reflect a reduced emphasis on combat for the Beta males of this species.

The relative warp analyses for the two dimorphic species contrast with the tri- and tetramorphic ones. In neither of these cases are the Alpha males are well separated from the Gamma males, and in *O. siva* there is complete overlap between the two groups. This suggests that although Alpha males of this species are investing in large mandibles, they are not investing in structures to compensate for the reduced bite force that must arise from the increased length of the output lever. It is possible that this reflects a different function for the mandibles: for example, it could be the case that Alpha males are using their mandibles mainly for signalling and not using them in combat as much as in the tri- and tetramorphic species.

The mechanisms behind the maintenance of these polymorphisms is not currently known. *O. brookeana, O. cuvera, O. sommeri lowei* and *O. platynota* all appear to have a single size threshold below which males tend to develop into Gamma morphs. This makes sense in terms of the status-dependent conditional evolutionary strategy^7,8^, with small males developing into non-combative morphs because their fitness would be reduced if they attempted to compete with larger males. Above this threshold, however, we see no relationship between body size and morph, even in *O. cuvera* which was previously described as having two thresholds^15^. The allometric plots for these species resemble the one for the trimorphic male harvestmen of the species *Pantopsalis cheliferoides* recently published by Painting and co-workers^18^. The authors proposed that in this case the polymorphism was maintained by a combination of a condition-dependent threshold and a genetic polymorphism, and this seems also to be a possible explanation for the patterns seen here. Given the diversity of weaponry in the tetramorphic species we tentatively propose that this complex polymorphism might be maintained by a genetic rock-paper-scissors interaction in males above the threshold size. This is, of course, speculative and behavioural and genetic studies of live animals are clearly needed to help resolve these issues.

## Methods

### Study species and datasets

We used the collection of *Odontolabis* held in the Natural History Museum, London for this study. We chose all of the species that were represented by more than thirty undamaged and appropriately mounted male specimens for study. These are: *Odontolabis brookeana* (locality: Borneo, Sumatra and Java), n = 43; *O. cuvera* (India, Vietnam), n = 164; *O. platynota* (North Myanmar, China, Laos), n = 51; *O. siva* (Burma, Laos, Vietnam), n = 144, *O. sommeri* (Borneo, Sumatra and Java), n = 36 as well as the subspecies *O. sommeri lowei* (n = 82). *O. sommeri lowei* is now recognised as a subspecies of *O. sommeri* ^38–40^, but initial analysis found that the allometric relationships between body size and mandible length differ between *O. sommeri s.s*. and *O. sommeri lowei* and so the two were analysed as separate species. The dorsal side of each specimen was photographed with a Nikon D3200 and Sigma 105-mm macro lens five separate times - one image was used for the measurements used in morph allocation, and all five were used for landmark digitisation for the geometric morphometric analysis.

### Morph allocation

The lengths of the left mandible and left elytron, the latter used as an indicator of body size, were measured from a single image of each specimen using ImageJ^41^. The left-hand side of body parts was chosen because many of the specimens were mounted with the tip of the left mandible overlapping the tip of the right mandible. In *O. sommeri lowei* only the distance between the two major teeth at the distal end of the mandible was also measured in all morphs except the Alpha morph in order to facilitate allocation of other males to different morphs. We allocated males to morphs initially on the basis of visual inspection, including checking for the differences in mandibles between alpha and beta males described in Rowland and Emlen^15^. We followed this visual inspection with the analysis procedures detailed in^29^ including examination of scatterplots, frequency histograms of the ratio of mandible length to body length and comparison of fitted models using candidate sets of morph allocations. We fitted finite mixture models using the R flexmix package^42^ to the mandible length: body length ratios (as in^43^ but using ratios) to assist in morph classification.

We chose not to use geometric morphometric approaches to morph allocation for several reasons. Firstly, Romiti *et al*.^23^ found, in the case of *Lucanus cervus*, that geometric morphometric approaches were unnecessary for capturing variation in mandible length, and that differences between males could be adequately described using a linear measurement only. Secondly, the mandibles of these animals are so variable that finding a suitable set of homologous landmarks would be very difficult - as an example, the Alpha males of several of these species have mandibles that are pointed at their distal end and do not express the teeth found in other morphs, and in other individuals of these species major teeth on the mandibles of Alpha males cannot be identified.

### Head and prothorax morphometrics

For the geometric morphometric analysis, a set of homologous morphological characters were chosen and represented by the coordinates of sets of landmark points^44^ from the right side of the head and prothorax. These were digitised using ImageJ and later converted to the thin-plate spline (TPS) file format (Fig. 1). Landmarks from the elytra were not employed in the analysis because there is sometimes a gap between the elytra and the prothorax which is a consequence of the specimen sagging during mounting. All five photographs per specimen were digitised for the geometric morphometric analysis and the measures combined in order to minimise any distortion from camera angle.

Analysis was carried out using the geomorph package running in R^45,46^. Generalised Procrustes Analysis (GPA) was used to standardise the data for the effects of scale, position and size^47,48^. The plot of the two-dimensional tangent space of principal component (PC) 1 and 2 were used to check for any errors in collecting landmarks. Relative warp analysis was then applied to all photographs, and principal component (PC) scores were calculated. Finally, the average PC values for all five images for each individual specimen were calculated. We used plots of PC1 against PC2 to visualise shape differences between morphs, combined with analyses using body size measurements as an explanatory variable (the latter presented in the supplementary material). TPS transformation grids produced using the programme tpsRelw^49^ were used to visualise how the shape of beetle heads and prothoraxes change along the PC axes.

### Data availability

The full dataset used here is available as a supplementary file, with the detailed analysis presented as the supplementary information.

## Electronic supplementary material

Supplementary information

Supplementary Dataset 1
