# Supplementary material for: Diverse and complex male polymorphisms in Odontolabis stag beetles (Coleoptera: Lucanidae)
Source: Sci Rep. 2017 Dec 1;7:16733. doi: 10.1038/s41598-017-17115-5 (PMC5711891; doi:10.1038/s41598-017-17115-5)
Supplement: Supplementary file 1 — Supplementary information [file 41598_2017_17115_MOESM1_ESM.pdf]

Diverse and complex male polymorphisms in *Odontolabis* stag beetles (Coleoptera: Lucanidae)

Supplementary information: morph allocation and analysis

Rob Knell
School of Biological and Chemical Sciences
Queen Mary University of London
Keita Matsomota
Department of Life Science, Natural History Museum, London

Contents

O. brookeana **2**
Model fitting for *O. brookeana* 5
Morphometrics for *O. brookeana* 7

O. cuvera **11**
Model fitting for *O. cuvera* 16
Morphometrics for *O. cuvera* 17

O. platynota **21**
Model fitting for *O. platynota* 24
Morphometrics for *O. platynota* 26

O. siva **30**
Model fitting for *O. siva* 33
Morphometrics for *O. siva* 35

O. sommeri lowei **38**
Model fitting for *O. sommeri lowei* 44
Morphometrics for *O. sommeri lowei* 47

O. sommeri s.stricto **52**
Model fitting for *O. sommeri s.s.* 57
Morphometrics for *O. sommeri s.stricto* 59

Morph proportions by species **63**

Figure with all species allometry **66**

Contents

O. brookeana

Odontolabis brookeana has four male morphs. There is a clearly separated group of six individuals with large mandibles who correspond morphologically to the Alpha morphs originally described for *O. cuvera* by Rowland and Emlen (2009), and there is a single individual in this sample which corresponds morphologically to the Beta males described by Rowland and Emlen, with a large tooth towards the base of the mandibles. This individual clusters with the small mandible males in the geometric morphometric plot, which supports its position as a Beta rather than just a weird alpha. Of the remainder of the specimens there are two morphs with short mandibles - a group of small males with small mandibles, which are equivalent to the minors in other species, and then a separate cluster of males with robust "boltcutter" mandibles. These can be distinguished visually and are also in separate groupings on the allometric plot.

A frequency histogram of the ratios of mandible length to elytra length has three peaks and a gaussian mixture model with three clusters gives a good fit to these. These correspond to the three morphs with more than one representative in the sample (Alpha, Beta and Gamma) and classification by the mixture model maps to the visual classification in most cases.

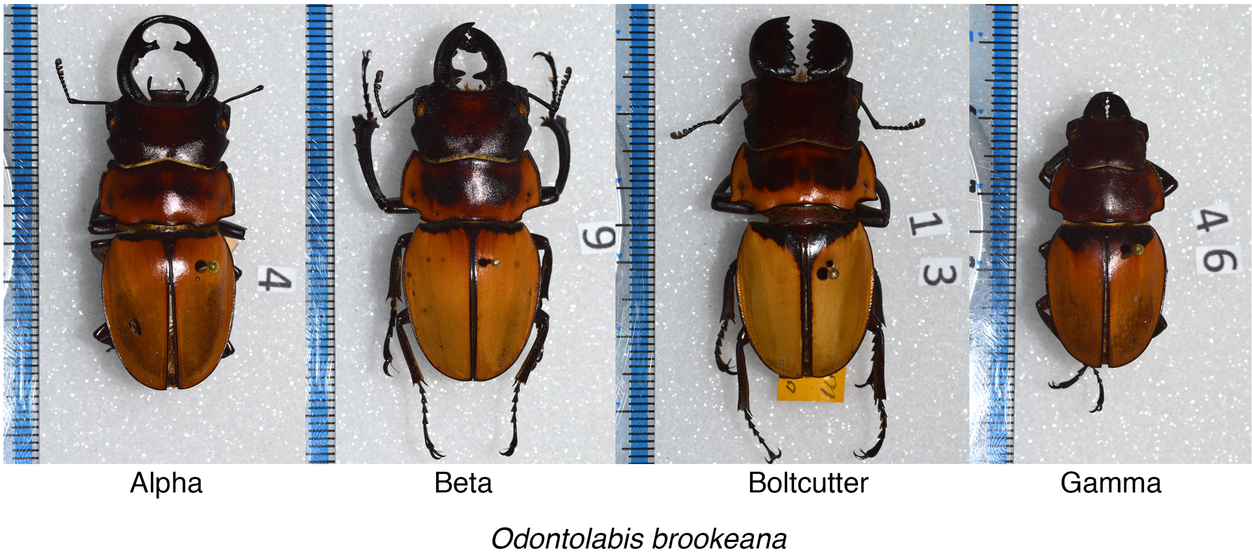

Figure S1 Examples of the different morphs for *O. brookeana*

```
brook <- subset(alldata, species == "brookeana")

# Set up new variable for ratio
ratio <- brook$left_mandible_straight/brook$elytra_middle



# Fit mixture model NB row 6 (Beta morph)
# excluded

mix.mod <- flexmix(ratio[-6] ~ 1, k = 3,
    cluster = (as.numeric(droplevels(brook$morph_visual[-6]))))
```

```r
#### plot histogram

x1 <- seq(min(ratio), max(ratio), length = 100)

counts <- table(clusters(mix.mod))


d1 <- dnorm(x1, parameters(mix.mod)[1, 1],
    parameters(mix.mod)[2, 1]) * counts[1]/sum(counts)

d2 <- dnorm(x1, parameters(mix.mod)[1, 2],
    parameters(mix.mod)[2, 2]) * counts[2]/sum(counts)

d3 <- dnorm(x1, parameters(mix.mod)[1, 3],
    parameters(mix.mod)[2, 3]) * counts[3]/sum(counts)

densities <- data.frame(x1, d1, d2, d3)

p1 <- ggplot(data = data.frame(ratio), aes(ratio,
    ..density..)) + geom_histogram(fill = "grey80",
    colour = "black", bins = 16) + theme_bw() +
    xlab("Mandible length / elytron length")

p1 <- p1 + geom_line(data = densities, aes(x = x1,
    y = d1))
p1 <- p1 + geom_line(data = densities, aes(x = x1,
    y = d2))
p1 <- p1 + geom_line(data = densities, aes(x = x1,
    y = d3))
p1
```

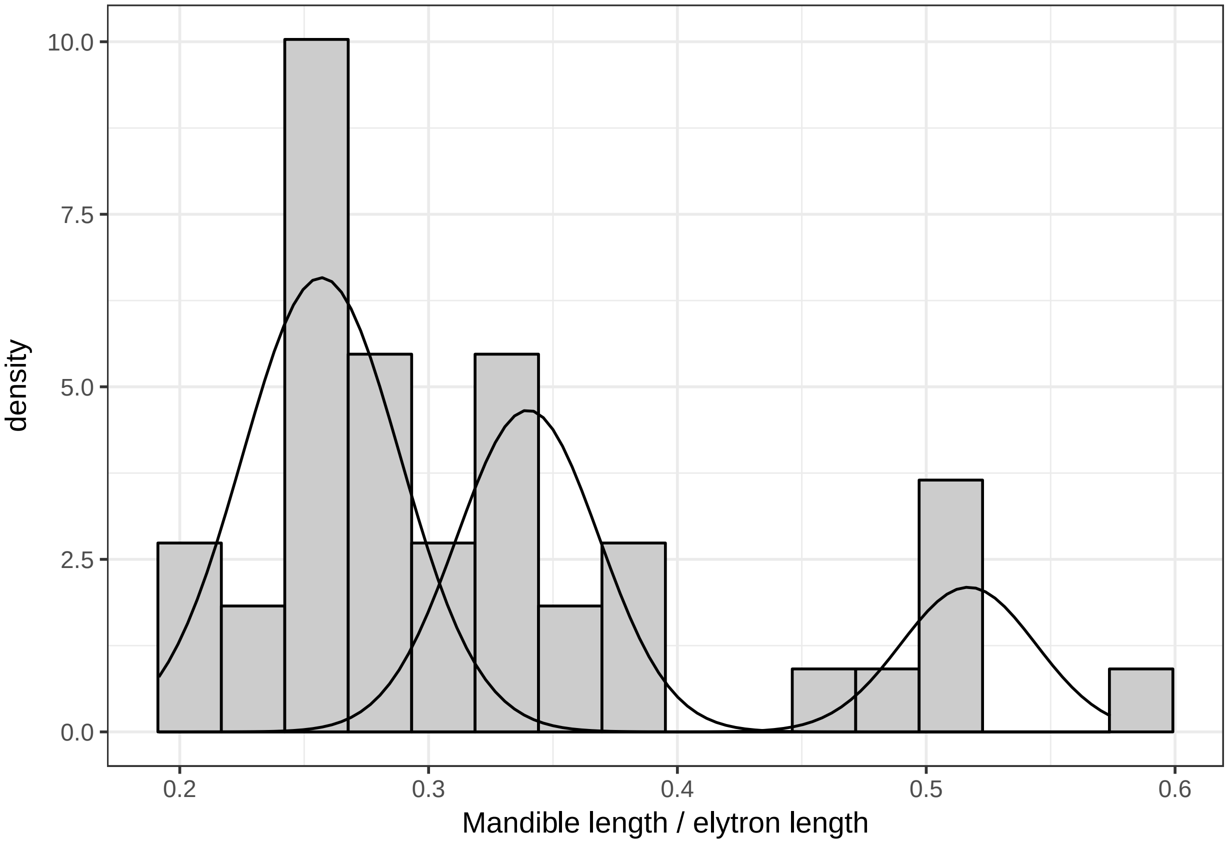

Figure S2 Histogram showing the frequency distribution of ratios of mandible length to elytra length for *O. brookeana* with the three fitted distributions from the mixture model.

The mixture model above classifies these animals into 6 alphas, 14 boltcutters and 22 gammas, plus the solitary beta which was excluded from the analysis. Inspection of mandible morphologies tells us that this division is not completely satisfactory since two small beetles which are clearly Gammas are misclassified as Boltcutters so the assignment is changed for these two specimens.

```
# palette1<-c('steelblue', 'orange', 'darkred')


# Morph <-
# ifelse(brook$left_mandible_straight/brook$elytra_middle >
# 0.45, 'Alpha', 'Gamma') Morph <-
# ifelse(brook$left_mandible_straight/brook$elytra_middle >
# 0.3 & Morph == 'Gamma', 'Boltcutter', Morph)

cluster <- c(clusters(mix.mod)[1:5], 4, clusters(mix.mod)[6:42])

Morph <- ifelse(cluster == 1, "Alpha", "Beta")
Morph <- ifelse(cluster == 2, "Boltcutter", Morph)
Morph <- ifelse(cluster == 3, "Gamma", Morph)

# Morph[6] <- 'Beta'
Morph[c(9, 26)] <- "Gamma"


ggplot(data = brook, aes(x = elytra_middle, y = left_mandible_straight,
```

```
    colour = Morph)) + geom_point(size = 2) + scale_colour_manual(values = palette2) +
    theme_bw() + xlab("Elytron length (mm)") + ylab("Mandible length (mm)")
```

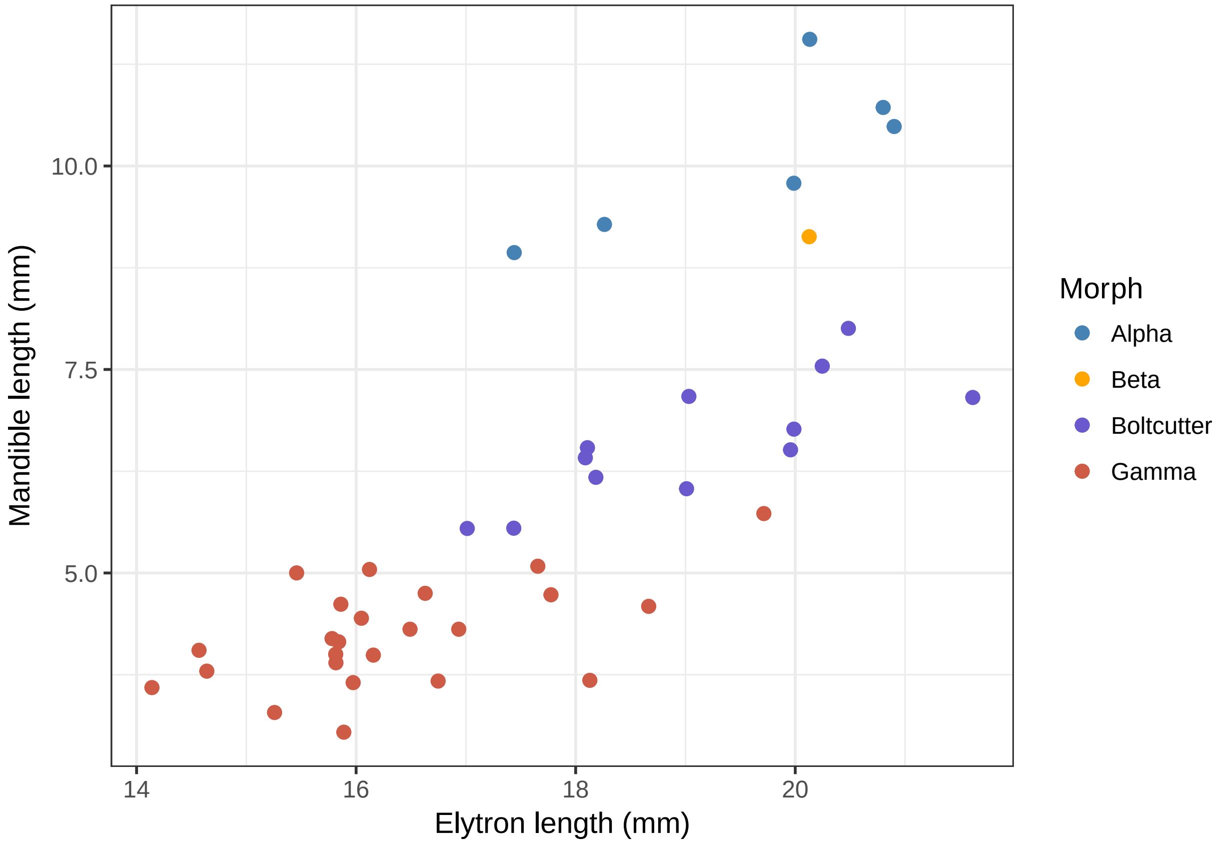

Figure S3 Mandible length against Elytron length with morph allocation shown for *O brookeana*

Model fitting for *O. brookeana*

Comparison of candidate models explaining the variation in mandible length.

mod1: Three morph, different slopes
mod2: Two morph (boltcutter and gamma combined), different slopes
mod3: Three morph, same slopes (no interaction)
mod4: Two morph, same slopes

```
Morph2 <- ifelse(Morph == "Boltcutter", "Gamma", Morph)

mod1 <- lm(log(left_mandible_straight) ~ Morph * log(elytra_middle),
    data = brook, subset = Morph != "Beta")
mod2 <- lm(log(left_mandible_straight) ~ Morph2 * log(elytra_middle),
    data = brook, subset = Morph != "Beta")
mod3 <- lm(log(left_mandible_straight) ~ Morph + log(elytra_middle),
    data = brook, subset = Morph != "Beta")
mod4 <- lm(log(left_mandible_straight) ~ Morph2 + log(elytra_middle),
    data = brook, subset = Morph != "Beta")

AIC(mod1, mod2, mod3, mod4)
```

```
##      df       AIC
## mod1  7 -57.94531
## mod2  5 -38.66344
## mod3  5 -61.61872
## mod4  4 -39.54322
```

A three-morph (plus the single beta which was excluded from this analysis) model is strongly supported: the AIC score for the same-slopes version is the lowest and so we accept this as the best description of our data.

```
brook_mod <- mod3

summary(brook_mod)
```

```
##
## Call:
## lm(formula = log(left_mandible_straight) ~ Morph + log(elytra_middle),
##     data = brook, subset = Morph != "Beta")
##
## Residuals:
##       Min        1Q    Median        3Q       Max
## -0.292747 -0.050302 -0.004455  0.074168  0.232934
##
## Coefficients:
##                     Estimate Std. Error t value Pr(>|t|)
## (Intercept)         -0.85384    0.68143  -1.253    0.218
## MorphBoltcutter     -0.40045    0.05454  -7.343 8.49e-09 ***
## MorphGamma          -0.68501    0.06470 -10.588 6.81e-13 ***
## log(elytra_middle)  1.06480    0.22875   4.655 3.88e-05 ***
## ---
## Signif. codes:  0 '***' 0.001 '**' 0.01 '*' 0.05 '.' 0.1 ' ' 1
##
## Residual standard error: 0.1084 on 38 degrees of freedom
## Multiple R-squared:  0.9117, Adjusted R-squared:  0.9047
## F-statistic: 130.8 on 3 and 38 DF,  p-value: < 2.2e-16
```

```
pred <- predict(brook_mod)  #predicted values from model

pred <- c(pred[1:5], NA, pred[6:42])  #add NA for the single beta morph
brook2 <- data.frame(brook, Morph, pred)


brookeana_plot1 <- ggplot(data = brook2, aes(x = log(elytra_middle),
    y = log(left_mandible_straight), colour = Morph)) + geom_point(size = 2) +
    scale_colour_manual(values = palette2) + theme_bw() + xlab("Log elytron length (mm)") +
    ylab("Log mandible length (mm)")

brookeana_plot1 <- brookeana_plot1 + geom_line(aes(y = pred),
    size = 0.33) + ggtitle("O. brookeana") + theme(plot.title = element_text(face = "italic"))

brookeana_plot1
```

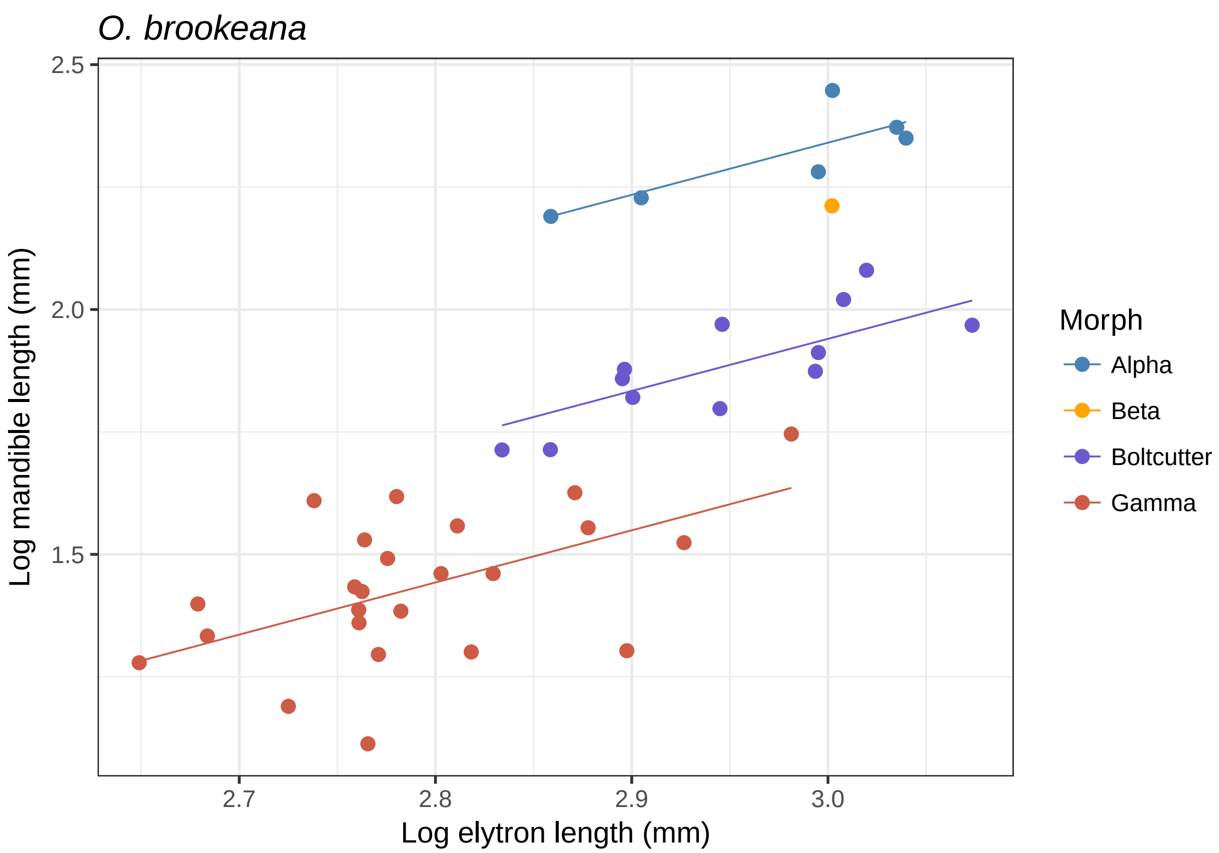

Figure S4 Final allometric plot for *O. brookeana* with morph allocations and lines from the fitted model.

Morphometrics for *O. brookeana*

```
# brook2<-data.frame(brook, Morph)

s = brook2 %>% split(brook2$Morph)

ch = s %>% # Compute which points are on the convex hull of each
# data.frame /!\ Check how 'chull' works
lapply(., function(el) chull(el$pc1, el$pc2))  # 'ch' now contains the
# row numbers of points on convex hull per sub-data.frame

# Get points for each sub-data.frame using names index
ch = lapply(names(ch), function(el) s[[el]][ch[[el]], ]) %>%
    do.call(rbind, .)  # Join all convex hull points in a single data.frame


brookeana_pca_plot <- ggplot(data = brook, aes(x = pc1, y = pc2,
    colour = Morph)) + geom_point(size = 2) + scale_colour_manual(values = palette2) +
    scale_fill_manual(values = palette2) + theme_bw() + xlab("Principal component 1") +
    ylab("Principal component 2")


brookeana_pca_plot <- brookeana_pca_plot + geom_polygon(data = ch,
```

```
    aes(fill = Morph, colour = NA), alpha = 0.2) + ggtitle("O. brookeana") +
    theme(plot.title = element_text(face = "italic"))

brookeana_pca_plot
```

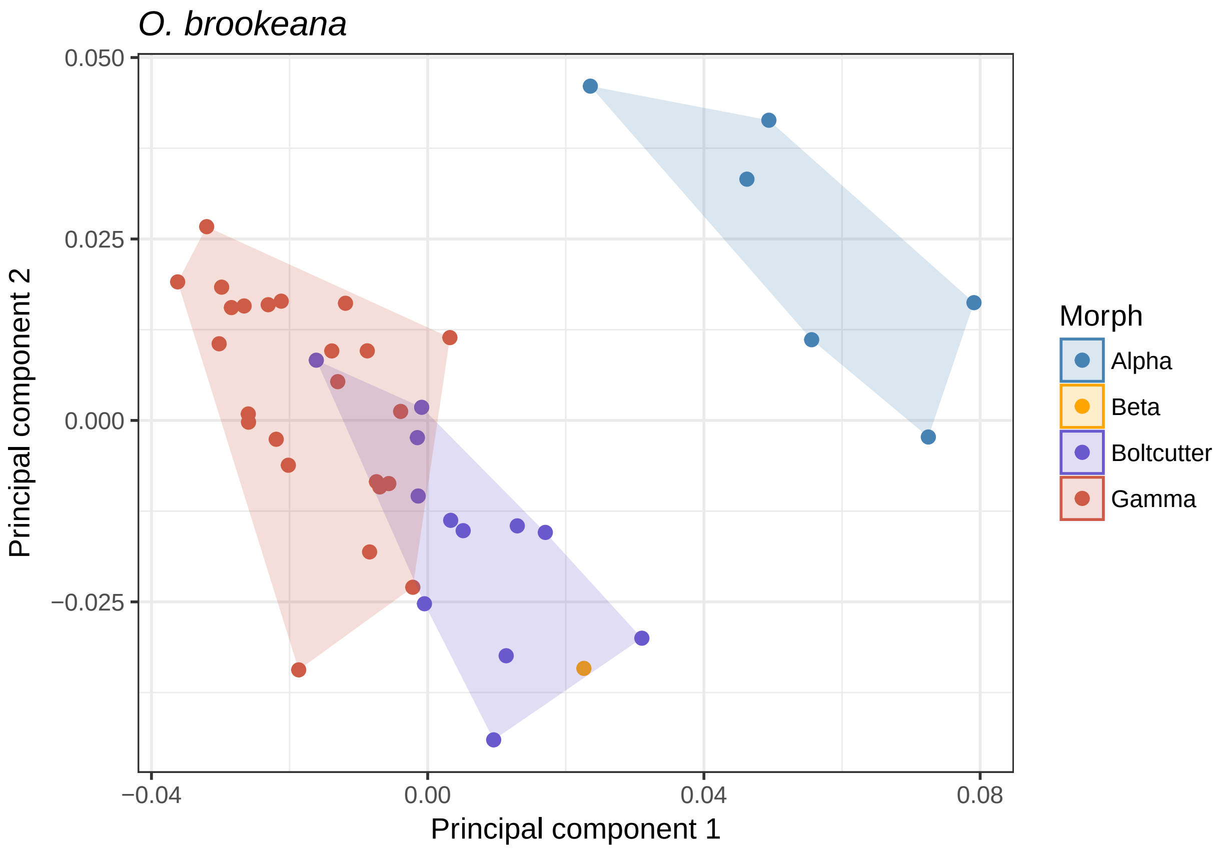

Figure S5 PC plot from relative warp analysis for *O. brookeana*

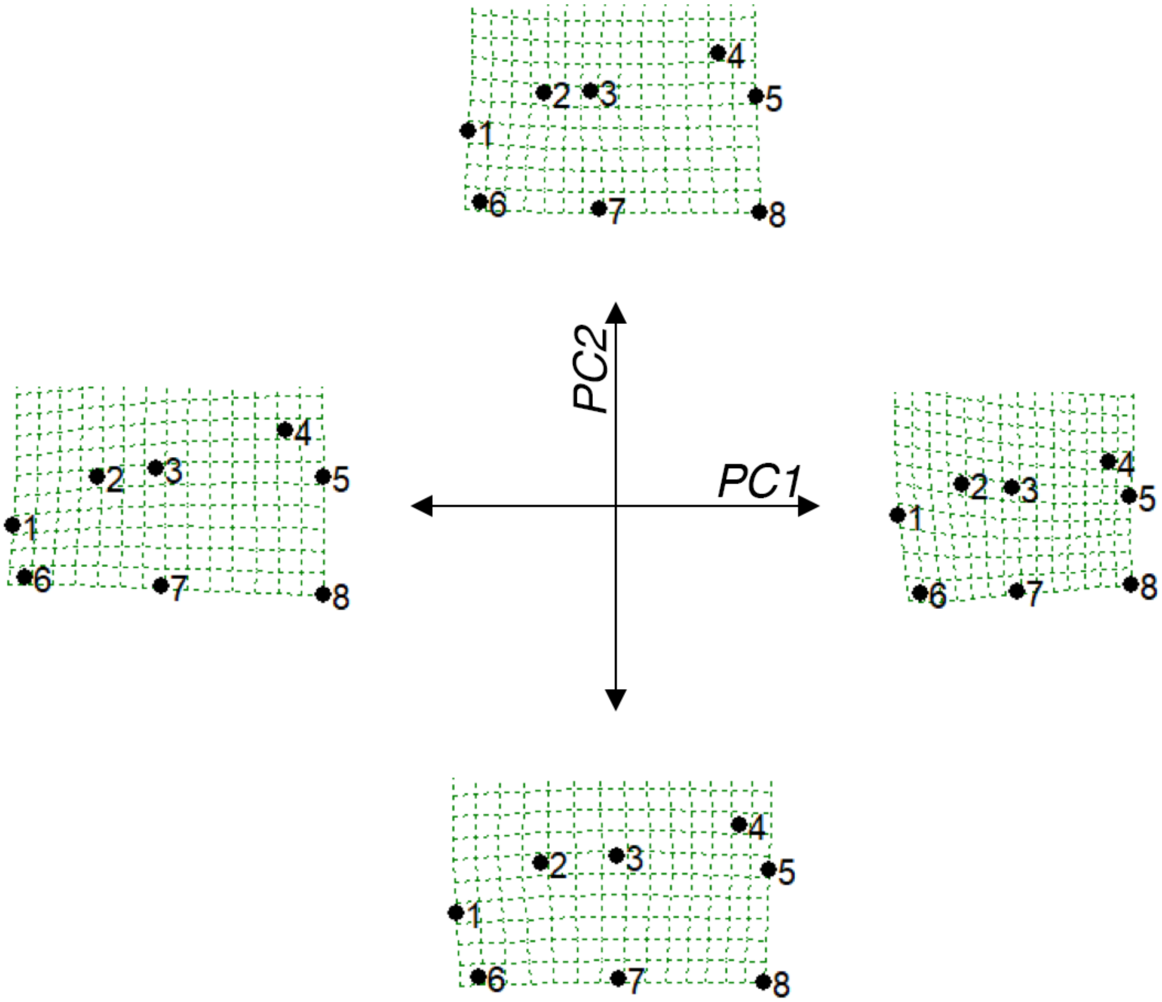

Figure S6 Thin plate splines showing how head and pronotum shape vary along the first two principal components for *O. brookeana*

The PCA indicates that the Alpha morphs are clustered separately from the other morphs, with some separation between the Gamma and Boltcutter morphs. PC1 is associated with the head becoming relatively larger compared to the prothorax, and the front of the head becoming relatively wider. PC2 is associated with the shape of the head changing so that positive values are associated with a reduced distance between the eye and the front of the prothorax. Alpha males, therefore, have relatively wide, more rectangular heads than other morphs, and have eyes that are set relatively further back than larger Beta or Boltcutter individuals.

A plot of body size (elytra length) against PC1 indicates that most of the differences between gamma and boltcutter morphs is a consequence of increasing size, but that alpha males are different from the others even when body size is taken into account. Fitting a set of candidate models to these data strongly supports the difference in shape for major morphs, with a model where the morphs were not distinguished having a much higher AIC than all models where Alpha males were identified by a factor in the analysis. The scores for models with and without Gamma and Boltcutter males were largely similar, however, and none of them was clearly better than the others: although the three-morph (not including the single Beta individual) separate-slopes model had the lowest AIC score it only differed from the three-morph same-slopes model by a very small amount and from the two two-morph models by a fraction more than two. We thus conclude that there is strong support for Alpha males having differently shaped heads and prothoraxes than others

independent of the effect of body size, but we cannot say with much certainty whether there are differences in morphology between the Boltcutter and Gamma morphs that arise from anything other than differences in body size.

```
# brook2<-data.frame(brook, Morph)

s = brook2 %>% split(brook2$Morph)

ch = s %>% # Compute which points are on the convex hull of each
# data.frame /!\ Check how 'chull' works
lapply(., function(el) chull(el$elytra_middle, el$pc1))  # 'ch' now contains the
# row numbers of points on convex hull per sub-data.frame

# Get points for each sub-data.frame using names index
ch = lapply(names(ch), function(el) s[[el]][ch[[el]], ]) %>%
    do.call(rbind, .)  # Join all convex hull points in a single data.frame


p1 <- ggplot(data = brook, aes(x = elytra_middle, y = pc1, colour = Morph)) +
    geom_point(size = 2) + scale_colour_manual(values = palette2) +
    scale_fill_manual(values = palette2) + theme_bw() + xlab("Elytra length (mm)") +
    ylab("Principal component 1")


p1 <- p1 + geom_polygon(data = ch, aes(fill = Morph, colour = NA),
    alpha = 0.2)
p1
```

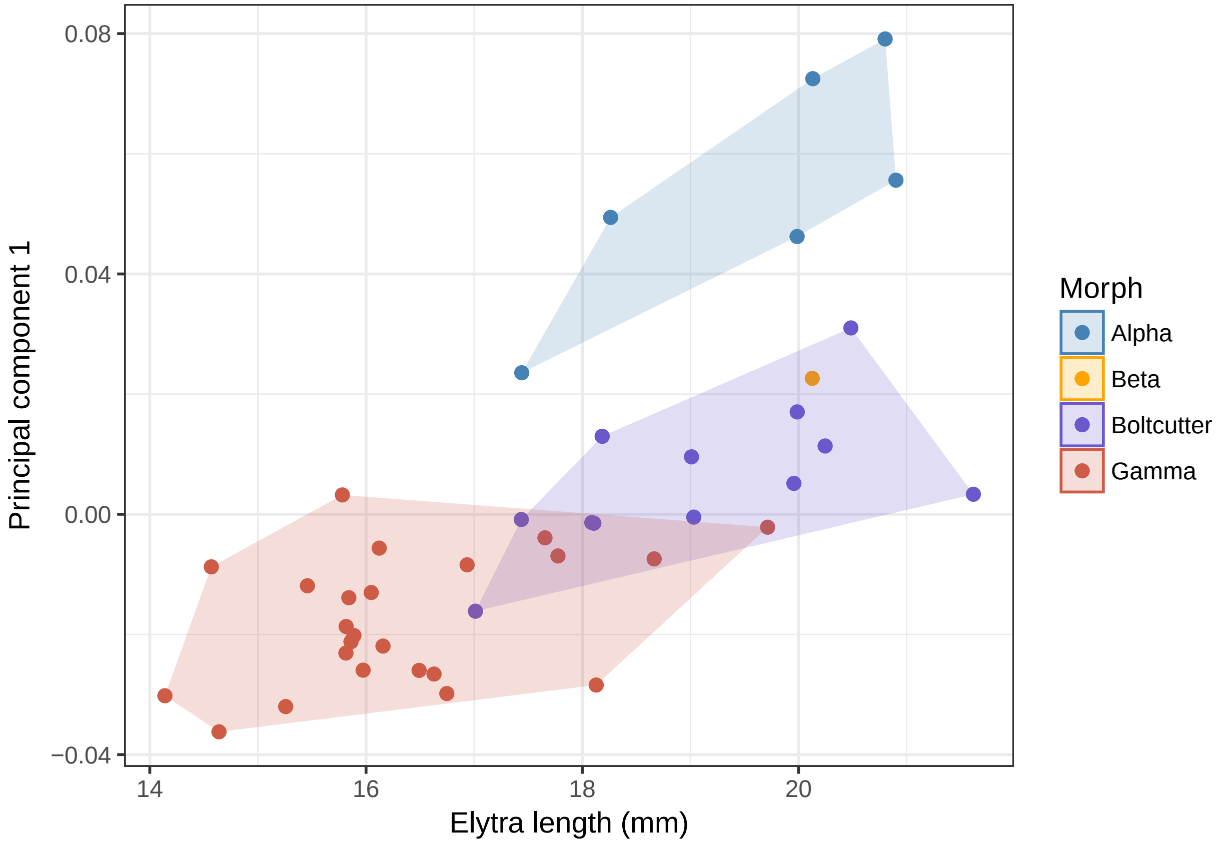

Figure S7 PC1 for *O. brookeana* from the relative warp analysis plotted against body size (elytron length)

We can further analyse this relationship by fitting a model explaining PC1 on the basis of morph and body size.

mod1: Three morph, different slopes
mod2: Two morph (boltcutter and gamma combined), different slopes
mod3: Three morph, same slopes (no interaction)
mod4: Two morph, same slopes
mod5: Body size (elytra length) only as an explanatory variable

```
Morph2 <- ifelse(Morph == "Boltcutter", "Gamma", Morph)

mod1 <- lm(pc1 ~ Morph * elytra_middle, data = brook, subset = Morph !=
    "Beta")
mod2 <- lm(pc1 ~ Morph2 * elytra_middle, data = brook, subset = Morph !=
    "Beta")
mod3 <- lm(pc1 ~ Morph + elytra_middle, data = brook, subset = Morph !=
    "Beta")
mod4 <- lm(pc1 ~ Morph2 + elytra_middle, data = brook, subset = Morph !=
    "Beta")
mod5 <- lm(pc1 ~ elytra_middle, data = brook, subset = Morph !=
    "Beta")


AIC(mod1, mod2, mod3, mod4, mod5)
```

```
##      df       AIC
## mod1  7 -255.8650
## mod2  5 -253.6835
## mod3  5 -255.1904
## mod4  4 -253.7695
## mod5  3 -208.5425
```

There is clear support for separating the Alpha morphs from the other animals, because the elytra length only model has a much higher AIC score than all the others. There is less certainty regarding whether we need a three morph or a two morph or whether a same- or different-slopes model is the best description of the data, since all of these models have AIC scores that are very similar, with only 2.18 separating the best and worst performing ones. The two three-morph models have lower AIC scores than the two-morph ones, suggesting that there are shape differences between these morphs that do not simply arise from body size effects, but we do not as yet have much confidence in this. A larger sample size is needed to provide clarity here.

```
Brook_Morph <- Morph
```

O. cuvera

```
cuvera <- subset(alldata, species == "cuvera")
```

Visual inspection of these specimens of *O. cuvera* found that they show similar trimorphism to that found by Rowland and Emlen for this species. There is an "Alpha" morph with large mandibles, with tooth 1 towards the distal end of the mandibles and a relatively small distance between teeth 2 and 3 (using the Rowland and Emlen numbering). The "Beta" morph also has large mandibles but the distance between teeth 2 and 3 is greater than in the Alpha males and tooth 1 is close to the base of the mandible. "Gamma" morphs have smaller mandibles that resemble those of females. Morphs were initially classified visually and a scatterplot of mandible length versus elytron length indicates that this produces a sensible grouping. Examination of a

histogram of the mandible length : elytron length ratio finds some evidence for trimodalism and a mixture model with three clusters gives groupings which correspond reasonably well to the visual classifications, although there are a number of individuals which were classified differently and which were examined in detail to allocate a morph.

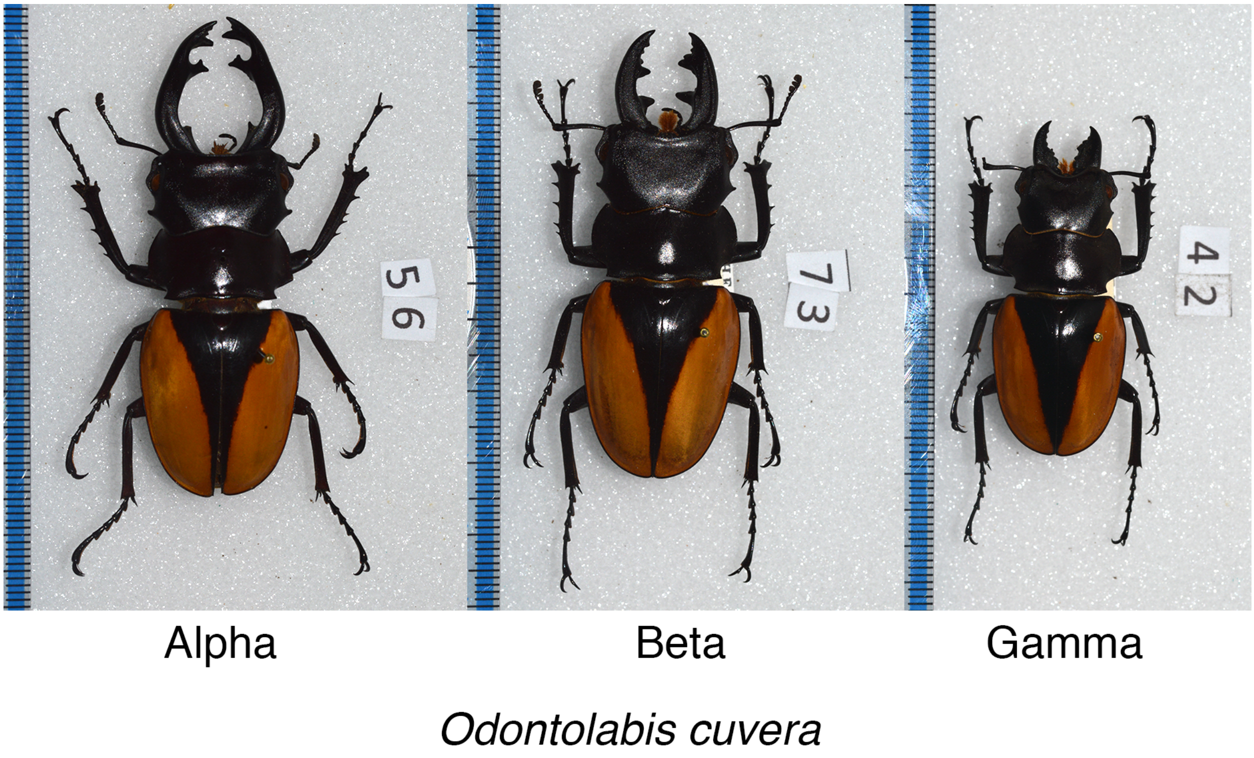

Figure S8 Examples of the different morphs for *O. cuvera*

```
# Set up new variable for ratio
ratio <- cuvera$left_mandible_straight/cuvera$elytra_middle



# Fit mixture model

mix.mod <- flexmix(ratio ~ 1, k = 3, cluster = as.numeric(droplevels(cuvera$morph_visual)))


#### plot histogram

x1 <- seq(min(ratio), max(ratio), length = 100)

counts <- table(clusters(mix.mod))

d1 <- dnorm(x1, parameters(mix.mod)[1, 1], parameters(mix.mod)[2,
    1]) * counts[1]/sum(counts)
d2 <- dnorm(x1, parameters(mix.mod)[1, 2], parameters(mix.mod)[2,
    2]) * counts[2]/sum(counts)
d3 <- dnorm(x1, parameters(mix.mod)[1, 3], parameters(mix.mod)[2,
    3]) * counts[3]/sum(counts)
```

```r
densities <- data.frame(x1, d1, d2, d3)

p1 <- ggplot(data = data.frame(ratio), aes(ratio, ..density..)) +
    geom_histogram(fill = "grey80", colour = "black", bins = 16) +
    theme_bw() + xlab("Mandible length / elytron length")

p1 <- p1 + geom_line(data = densities, aes(x = x1, y = d1))
p1 <- p1 + geom_line(data = densities, aes(x = x1, y = d2))
p1 <- p1 + geom_line(data = densities, aes(x = x1, y = d3))
p1
```

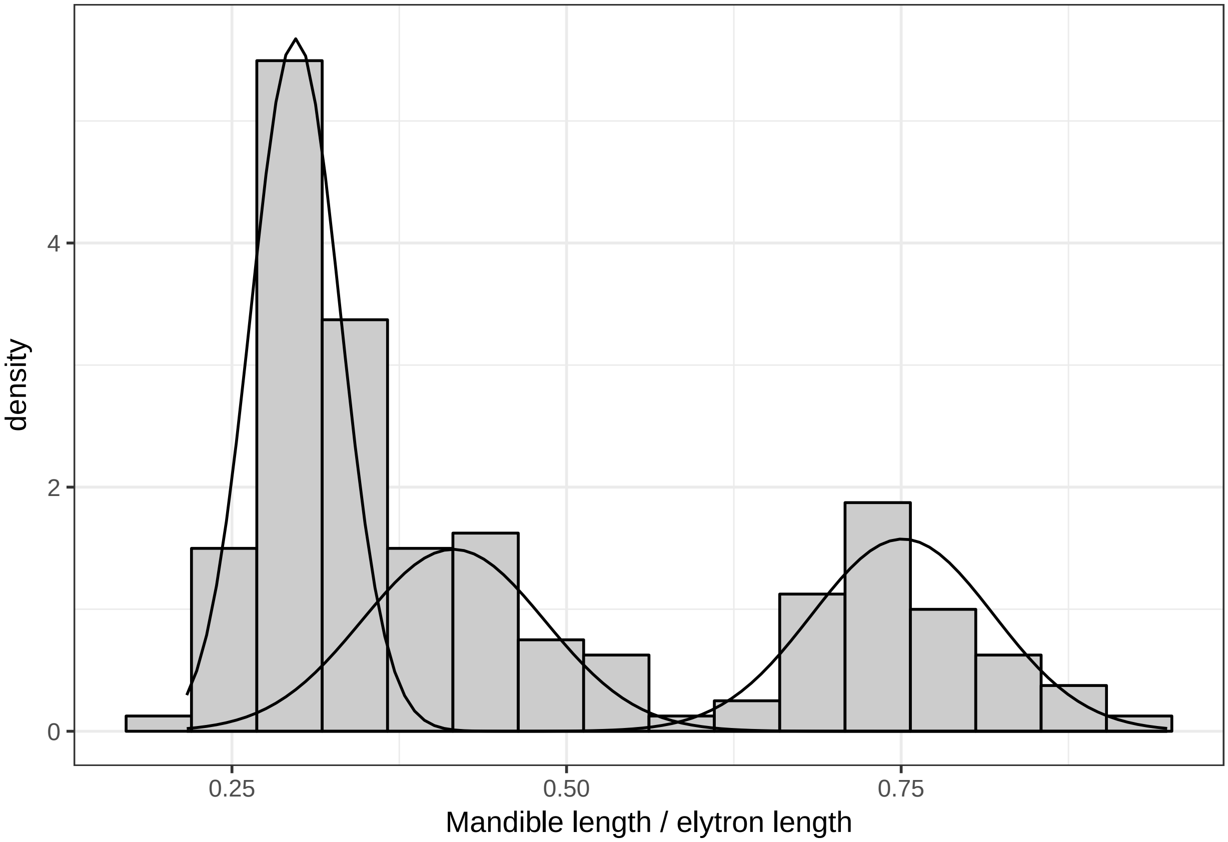

Figure S9 Histogram showing the frequency distribution of ratios of mandible length to elytra length for *O. cuvera* with the fitted normal distributions from the mixture model.

```r
Morph <- ifelse(cuvera$morph_visual == "Alpha", "Alpha", "Beta")
Morph[cuvera$morph_visual == "Gamma"] <- "Gamma"


Morph2 <- ifelse(clusters(mix.mod) == 1, "Alpha", "Beta")
Morph2 <- ifelse(clusters(mix.mod) == 3, "Gamma", Morph2)

# cuvera<-data.frame(cuvera, Morph, Morph2)


palette1 <- c("steelblue", "darkgreen", "coral3")
```

```r
ggplot(data = cuvera, aes(x = elytra_middle, y = left_mandible_straight,
    colour = Morph, shape = Morph2)) + geom_point(size = 2) +
    scale_colour_manual(values = palette1) + theme_bw() + xlab("Elytron length (mm)") +
    ylab("Mandible length")
```

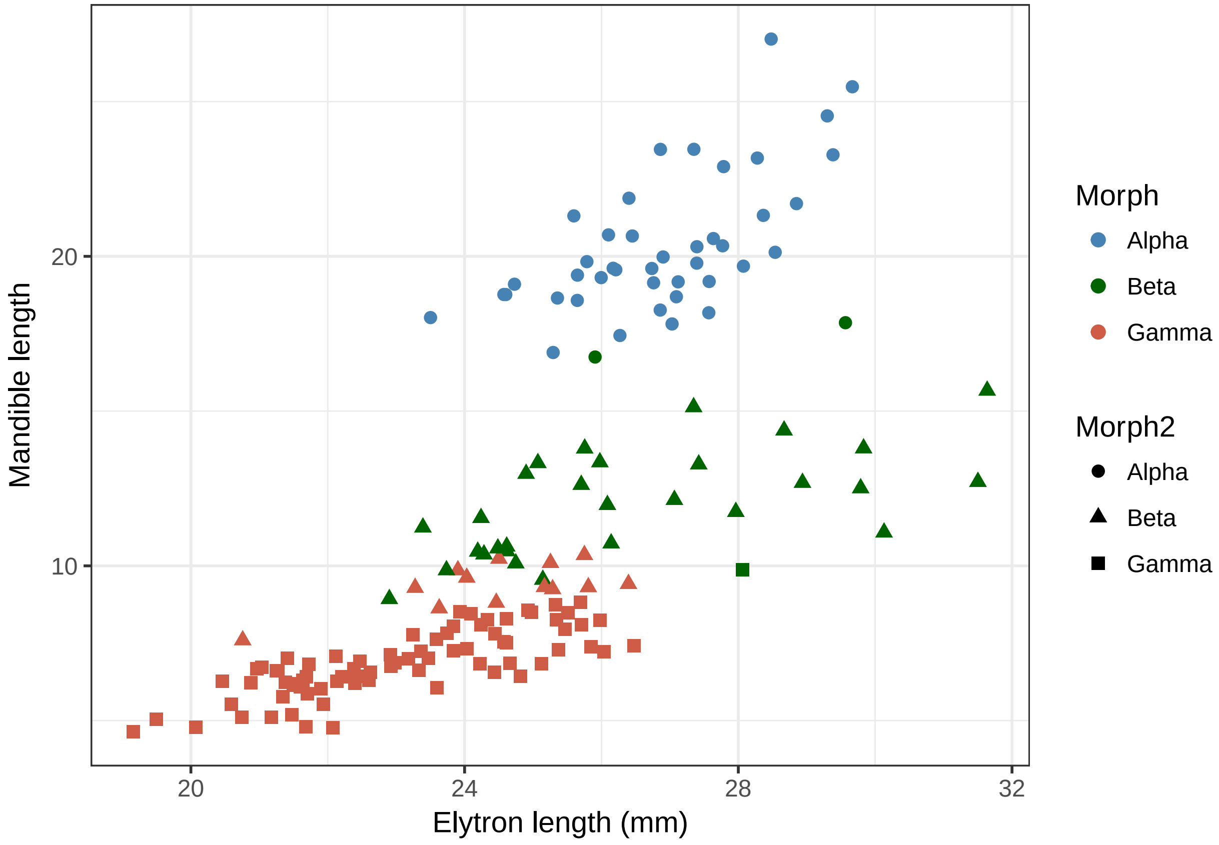

Figure S10 Mandible length against Elytron length with the initial morph allocation shown for *O brookeana*. "Morph" is the allocations made visually, "Morph2" is the allocations made by the mixture model.

Individuals which were classified differently were checked individually. The ones which are alphas on the division made from the mixture model and betas on the visual allocation were rows 19 and 94, photos 101 and 566. Both are more like betas than alphas although the mandible mirphology is really intermediate between the two. They are classed as betas here.

One beetle was classified as a gamma by the mixture model and as a beta from visual inspection. This was row number 93, photo 556 which is somewhat intermediate but probably best classified as a large gamma.

Fourteen beetles were classified as beta morphs from the mixture model but gamma morphs from the visual inspection. These are 24, 32, 39, 41, 42, 67, 68, 71, 88, 97, 116, 120, 142 & 149

24 is photo 126 and is a gamma 32 is 166 and is a gamma 39 is 211 and is a gamma 41 is 221 and is a gamma (albeit very intermediate) 42 is 231 and is a beta 67 is 411 and is a beta 68 is 416 and is a beta 71 is 436 and is a gamma 88 is 531 and is a beta 97 is 591 and is a gamma 116 is 691 and is missing so removed from the analysis. 120 is 711 and is missing so removed from the analysis. 142 is 831 and is missing so removed from the analysis. 149 is 876 and is a gamma.

Three photos are not in the set I have and I have checked all of the pictures that I was given. None correspond to these beetles (NB we have 150 photos and 164 beetles in the dataset) and on this basis they are removed from the analysis.

Final classification:

```r
Morph[93] <- "Gamma"

betas <- c(42, 67, 68, 88)

Morph[betas] <- "Beta"

rm(betas)

removed <- c(116, 120, 142)

Morph <- Morph[-removed]

cuvera <- cuvera[-removed, ]

rm(removed)

cuvera <- data.frame(cuvera, Morph)


ggplot(data = cuvera, aes(x = elytra_middle, y = left_mandible_straight,
    colour = Morph)) + geom_point(size = 2) + scale_colour_manual(values = palette1) +
    theme_bw() + xlab("Elytron length (mm)") + ylab("Mandible length")
```

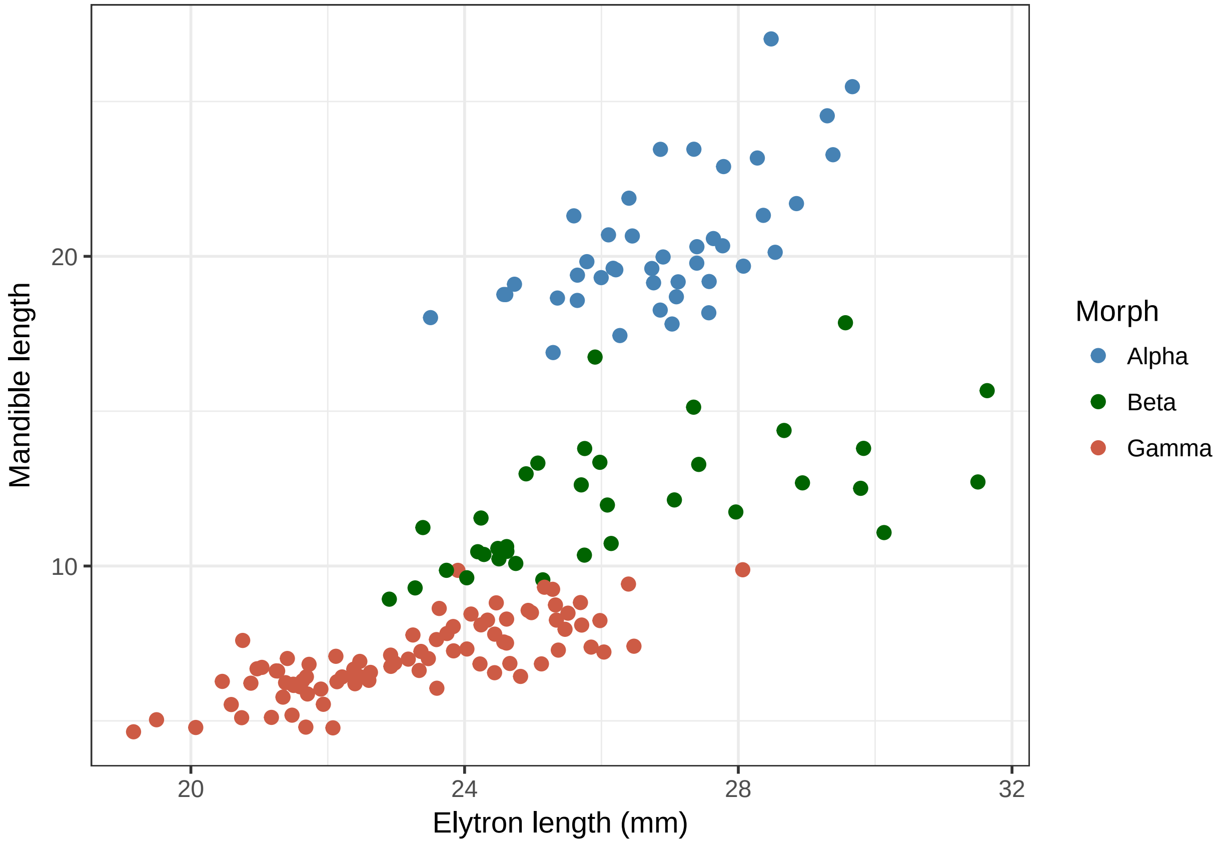

Figure S11 Mandible length against Elytron length with the revised morph allocation shown for *O brookeana*

Model fitting for *O. cuvera*

Comparison of candidate models explaining the variation in mandible length.

mod1: three morphs, different slopes
mod2: two morphs (betas and gammas combined), different slopes
mod3: three morphs, same slopes (no interaction) mod4: two morphs, same slopes

```
morph2 <- factor(ifelse(cuvera$Morph == "Beta", "Gamma", as.character(cuvera$Morph)))

mod1 <- lm(log(left_mandible_straight) ~ log(elytra_middle) *
    Morph, data = cuvera)
mod2 <- lm(log(left_mandible_straight) ~ log(elytra_middle) *
    morph2, data = cuvera)
mod3 <- lm(log(left_mandible_straight) ~ log(elytra_middle) +
    Morph, data = cuvera)
mod4 <- lm(log(left_mandible_straight) ~ log(elytra_middle) +
    morph2, data = cuvera)


AIC(mod1, mod2, mod3, mod4)
```

```
##      df       AIC
## mod1  7 -251.3450
## mod2  5 -134.1886
## mod3  5 -251.2235
## mod4  4 -130.4703
```

The three morph models both have much lower AIC scores than the two morph models, indicating that they provide the better explanation of the patterns in the data, providing further support to the idea that we have three morphs for *O. cuvera*. We cannot easily distinguish between the same-slopes and different-slopes three morph models, however, because the AIC scores for both are very similar, inidicating that each model is roughly as good as the other in epxlaining the variation in the data. The plot shows the same-slopes model.

```
cuvera_mod <- mod3

pred <- predict(cuvera_mod)  #predicted values from model

cuvera2 <- cbind(cuvera, pred)


cuvera_plot1 <- ggplot(data = cuvera2, aes(x = log(elytra_middle),
    y = log(left_mandible_straight), colour = Morph)) + geom_point(size = 2) +
    scale_colour_manual(values = palette1) + theme_bw() + xlab("Log elytron length (mm)") +
    ylab("Log mandible length (mm)")

cuvera_plot1 <- cuvera_plot1 + geom_line(aes(y = pred), size = 0.33) +
    ggtitle("O. cuvera") + theme(plot.title = element_text(face = "italic"))

cuvera_plot1
```

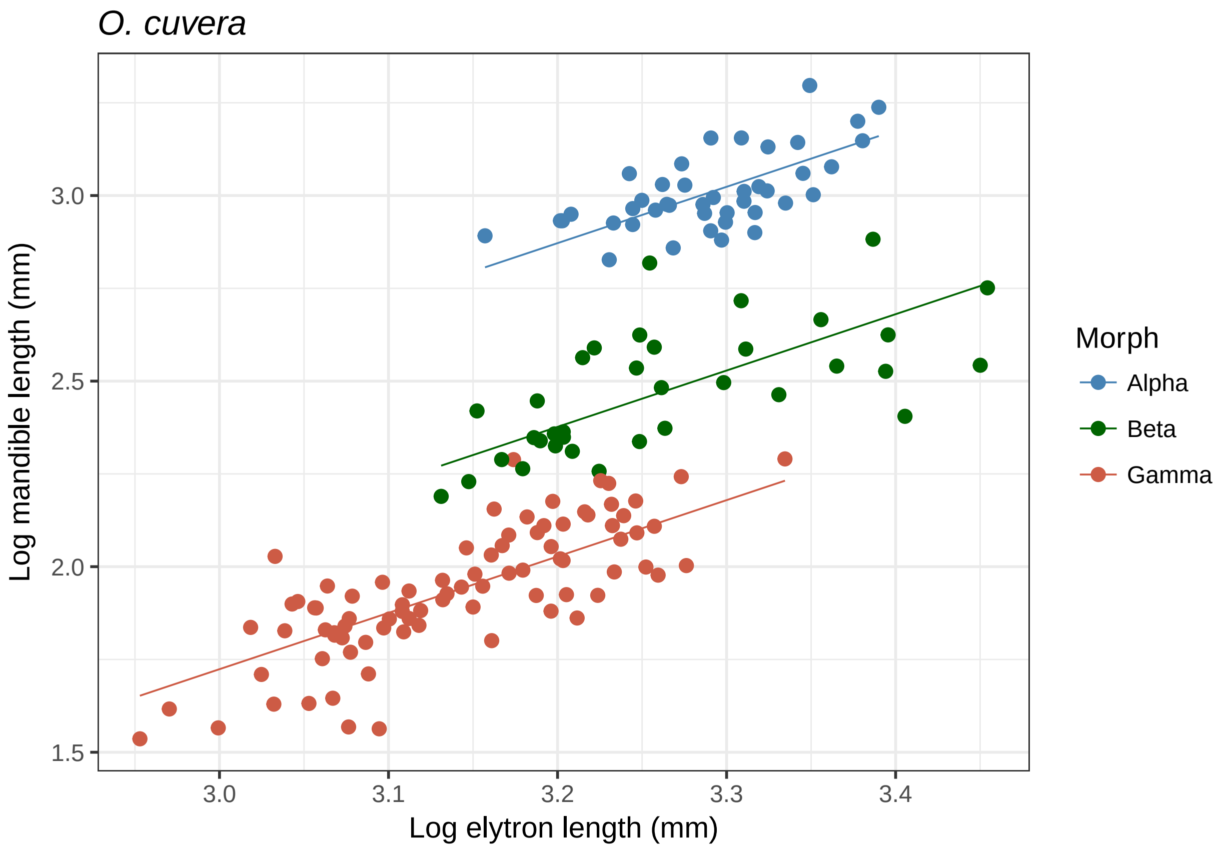

Figure S12 Final allometric plot for *O. cuvera* with morph allocations and lines from the fitted model.

Morphometrics for *O. cuvera*

```
s = cuvera %>% split(cuvera$Morph)

ch = s %>% # Compute which points are on the convex hull of each
# data.frame /!\ Check how 'chull' works
lapply(., function(el) chull(el$pc1, el$pc2))  # 'ch' now contains the
# row numbers of points on convex hull per sub-data.frame

# Get points for each sub-data.frame using names index
ch = lapply(names(ch), function(el) s[[el]][ch[[el]], ]) %>%
    do.call(rbind, .)  # Join all convex hull points in a single data.frame


# p1<-ggplot(data = brook, aes(x = pc1, y = pc2, colour =
# Morph)) + geom_point(size = 2) +
# scale_colour_brewer(palette = 'Set1') +
# scale_fill_brewer(palette = 'Set1') + theme_bw() +
# xlab('Principal component 1') + ylab('Principal component
# 2')

cuvera_pca_plot <- ggplot(data = cuvera, aes(x = pc1, y = pc2,
    colour = Morph)) + geom_point(size = 2) + scale_colour_manual(values = palette1) +
```

```
    scale_fill_manual(values = palette1) + theme_bw() + xlab("Principal component 1") +
    ylab("Principal component 2")


cuvera_pca_plot <- cuvera_pca_plot + geom_polygon(data = ch,
    aes(fill = Morph, colour = NA), alpha = 0.2) + ggtitle("O. cuvera") +
    theme(plot.title = element_text(face = "italic"))

cuvera_pca_plot
```

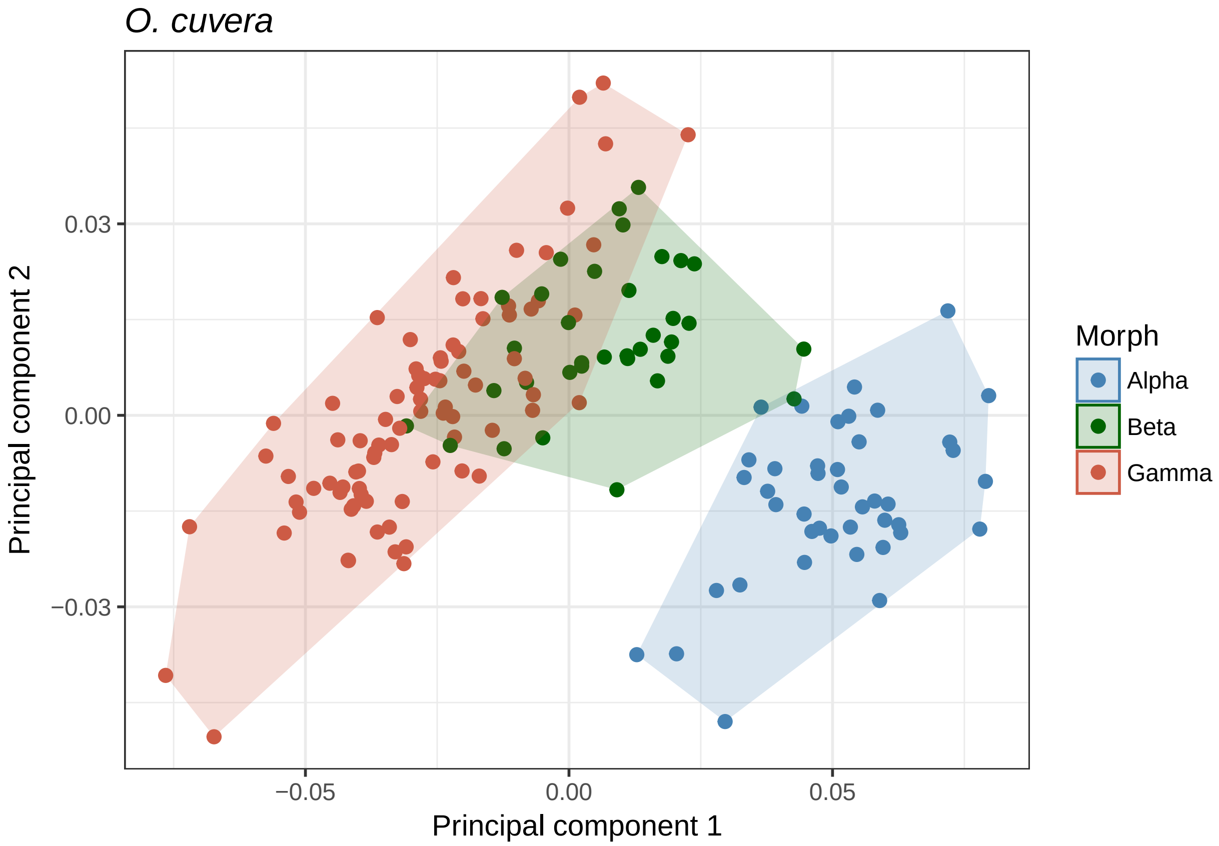

Figure S13 PC plot from relative warp analysis for *O. cuvera*

The pca plot shows the 'horseshoe' effect which indicates that there is one strongly influential axis affecting the PCA, but we can also see that there is a substantial effect of morph, with Alphas clustering separately from Betas and Gammas. The two Beta morphs which cluster with the Alphas are row numbers 19 and 94 - these are the two individuals which group with the Alphas in the allometry plot and which we earlier remarked upon as being somewhat intermediate between the alphas and the betas.

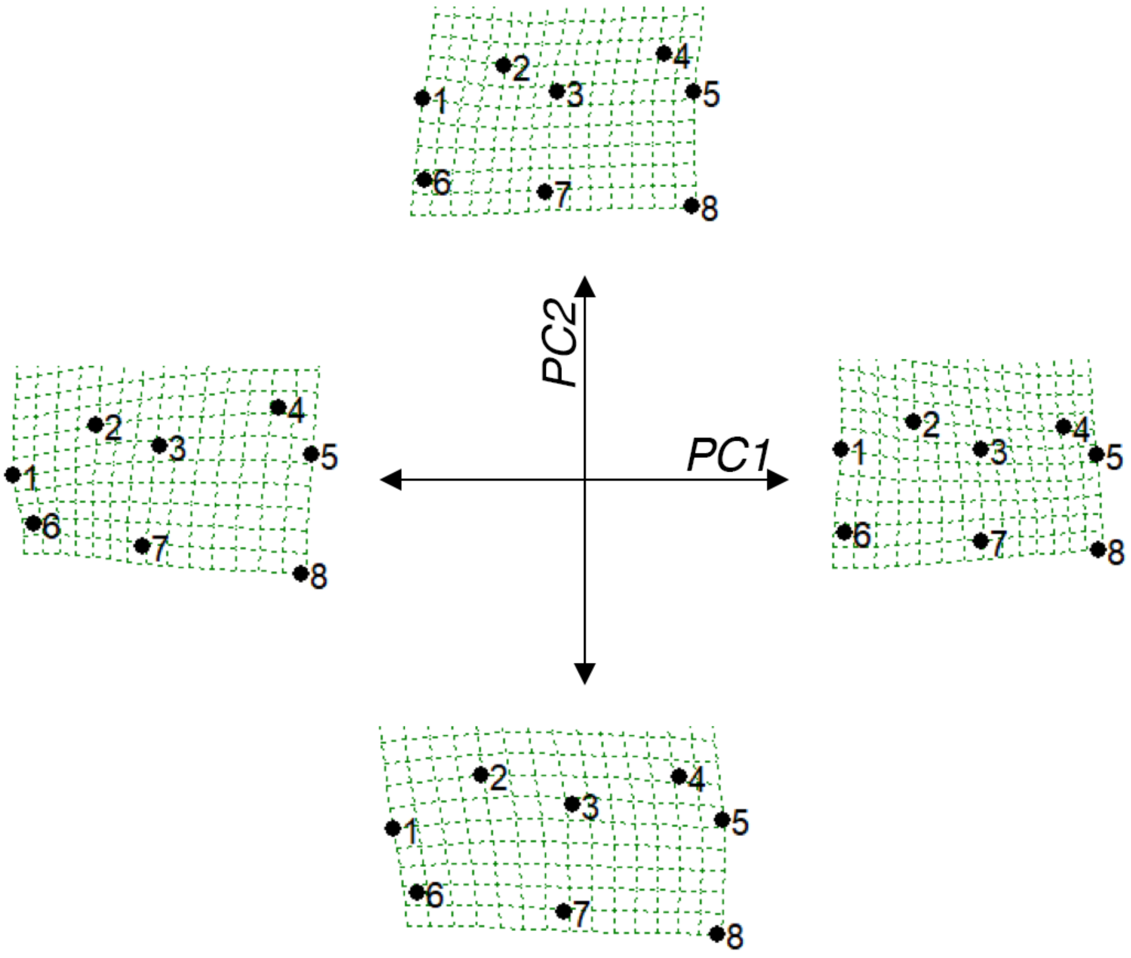

Figure S14 Thin plate splines showing how head and pronotum shape vary along the first two principal components for *O. cuvera*

Because PC2 is not especially informative in separating the morphs we will mainly look at PC1. As with *O. brookeana* this is associated with a change in head shape: a high positive PC1 value means that the animal has a relatively larger head and smaller prothorax, with the front of the head being much wider than for animals with a negative value for PC1. Alpha morphs in particular have large, more rectangular heads.

We can also look at the relationship between size and PC1 and we find that PC1 does vary with size, but also varies with morph independently of size. Further examination of the model shows us that all three factor levels are significantly different in Morph, indicating that all three morphs vary in their head and prothorax morphology.

```
ggplot(data = cuvera, aes(x = elytra_middle, y = pc1, colour = Morph)) +
    geom_point() + theme_bw() + scale_colour_manual(values = palette1) +
    geom_smooth(method = "lm", se = FALSE) + xlab("Elytron length (mm)")
```

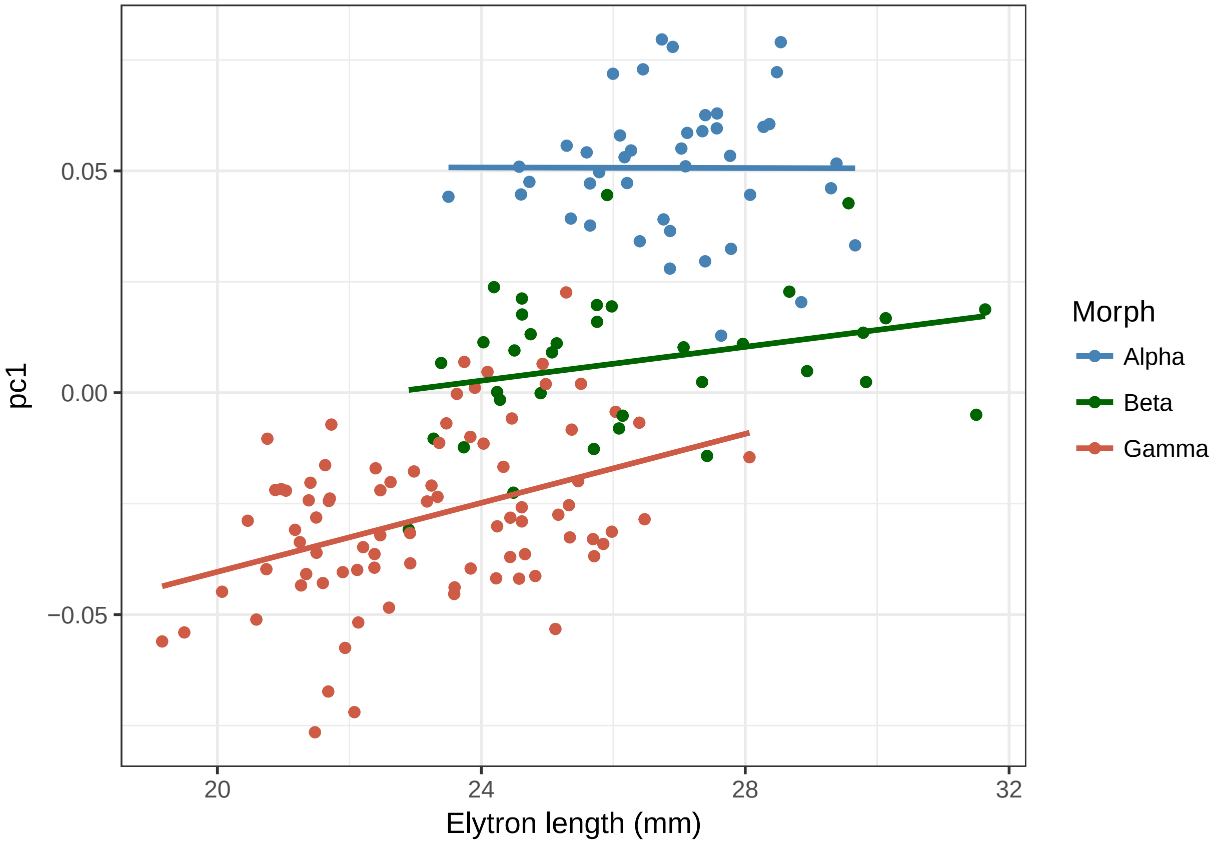

Figure S15 Body size (elytron length) and PC1 from the geometric morphometrics analysis. Lines show the predicted values form the best fit model.

mod1: Three morph, different slopes
mod2: Two morph (Beta and gamma combined), different slopes
mod3: Three morph, same slopes (no interaction)
mod4: Two morph, same slopes
mod5: body size (elytra length) only

```
Morph3 <- cuvera$Morph
Morph3[which(Morph3 == "Beta")] <- "Gamma"

mod1 <- lm(pc1 ~ elytra_middle * Morph, data = cuvera)
mod2 <- lm(pc1 ~ elytra_middle * Morph3, data = cuvera)
mod3 <- lm(pc1 ~ elytra_middle + Morph, data = cuvera)
mod4 <- lm(pc1 ~ elytra_middle + Morph3, data = cuvera)
mod5 <- lm(pc1 ~ elytra_middle, data = cuvera)

AIC(mod1, mod2, mod3, mod4, mod5)
```

```
##      df       AIC
## mod1  7 -858.2463
## mod2  5 -823.7215
## mod3  5 -858.0306
## mod4  4 -818.5712
## mod5  3 -690.6804
```

This analysis gives us strong support for there being shape differences between all three morphs that are not simply associated with body size. The two three morph models (mod1 and mod3) have much lower AIC

scores than the others but are not themselves easily distinguished, so it is unclear whether the slopes of the relationships between body size and pc1 vary between morphs or just the intercepts.

```
Cuvera_Morph <- Morph
```

O. platynota

Male *O. platynota* are dimorphic, with males with an elytron length of more than 15mm growing relatively larger mandibles than males with elytra shorter than 15mm. This can be seen in the mandible length / elytra length ratio histogram and is confirmed by visual examination of the scatterplot. Allocation of individuals to morphs via a mixture model with two clusters corresponds to the visual allocation.

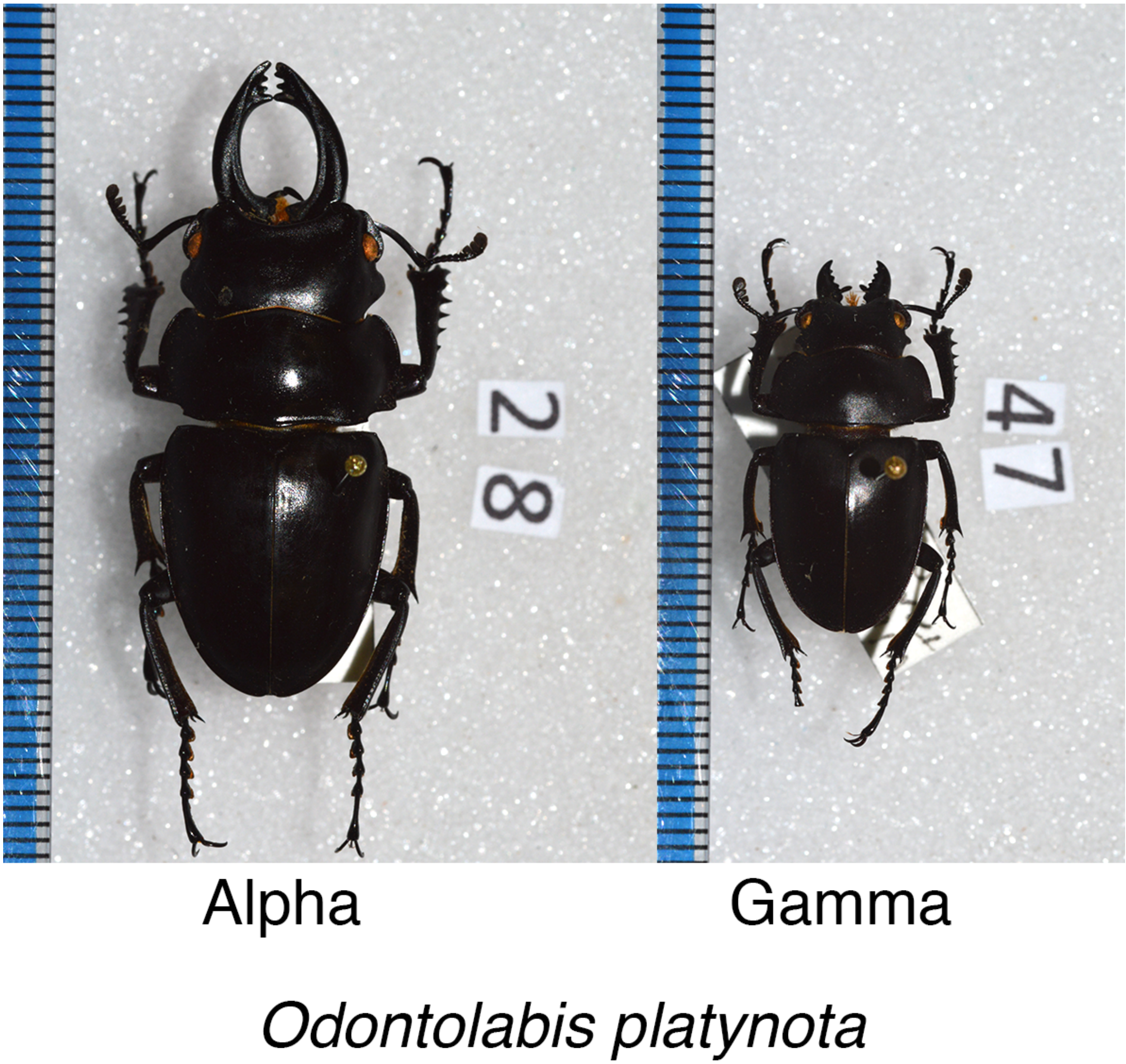

Figure S16 Examples of the different morphs for *O. platynota*

```r
platy <- subset(alldata, species == "platynota")


# Set up new variable for ratio
ratio <- platy$left_mandible_straight/platy$elytra_middle

mix.mod <- flexmix(ratio ~ 1, k = 2, cluster = as.numeric(droplevels(platy$morph_visual)))




#### plot histogram

x1 <- seq(min(ratio), max(ratio), length = 100)

counts <- table(clusters(mix.mod))


d1 <- dnorm(x1, parameters(mix.mod)[1, 1], parameters(mix.mod)[2,
    1]) * counts[1]/sum(counts)

d2 <- dnorm(x1, parameters(mix.mod)[1, 2], parameters(mix.mod)[2,
    2]) * counts[2]/sum(counts)


# densities <- data.frame(x1, d1, d2, d3)
densities <- data.frame(x1, d1, d2)

p1 <- ggplot(data = data.frame(ratio), aes(ratio, ..density..)) +
    geom_histogram(fill = "grey80", colour = "black", bins = 16) +
    theme_bw() + xlab("Mandible length / elytron length")

p1 <- p1 + geom_line(data = densities, aes(x = x1, y = d1))
p1 <- p1 + geom_line(data = densities, aes(x = x1, y = d2))

p1
```

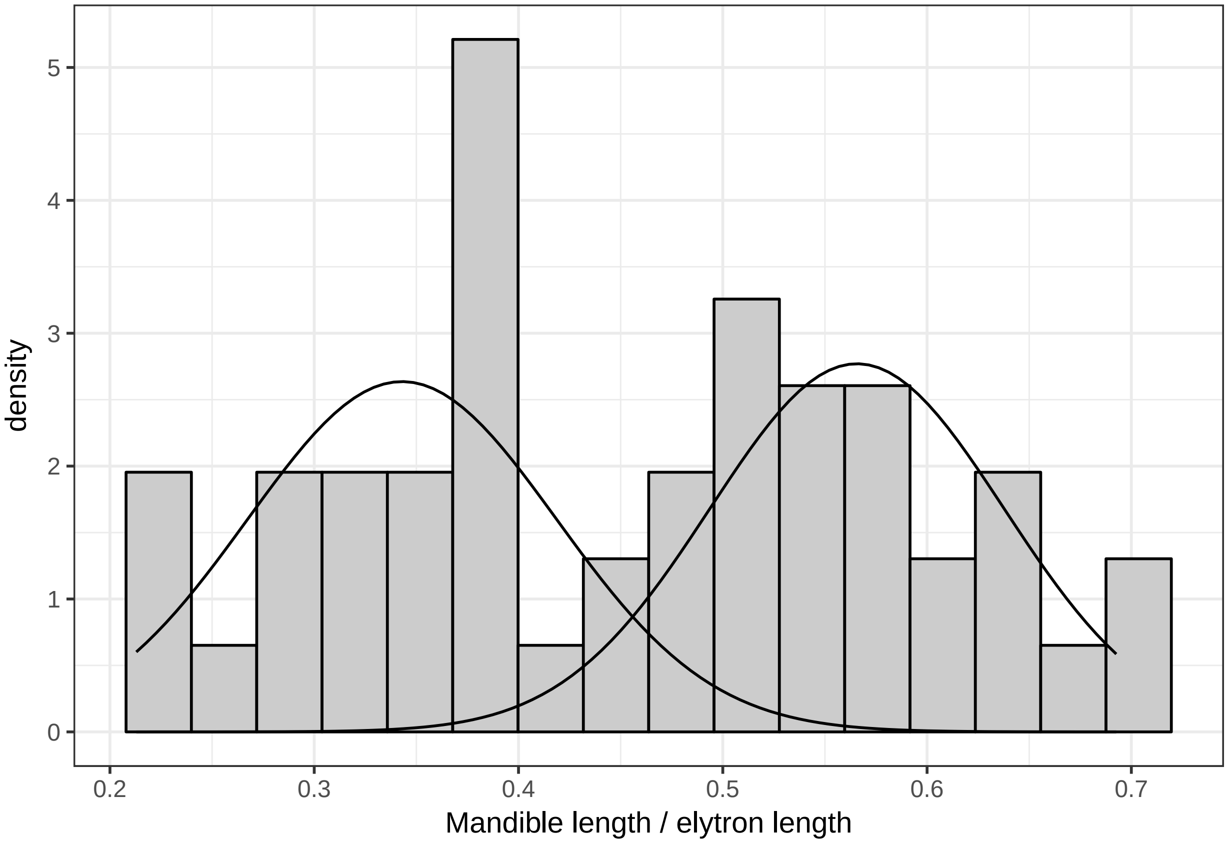

Figure S18 Histogram showing the frequency distribution of ratios of mandible length to elytra length for *O. platynota*.

The allocation to clusters from this mixture model corresponds to initial visual allocation and is shown below.

```
Morph <- ifelse(clusters(mix.mod) == 2, "Gamma", "Alpha")

platy <- data.frame(platy, Morph)

Platy_Morph <- Morph

rm(Morph)

ggplot(data = platy, aes(x = elytra_middle, y = left_mandible_straight,
    colour = Morph)) + scale_colour_manual(values = palette3) +
    geom_point(size = 2) + theme_bw() + xlab("Elytron length (mm)") +
    ylab("Mandible length (mm)")
```

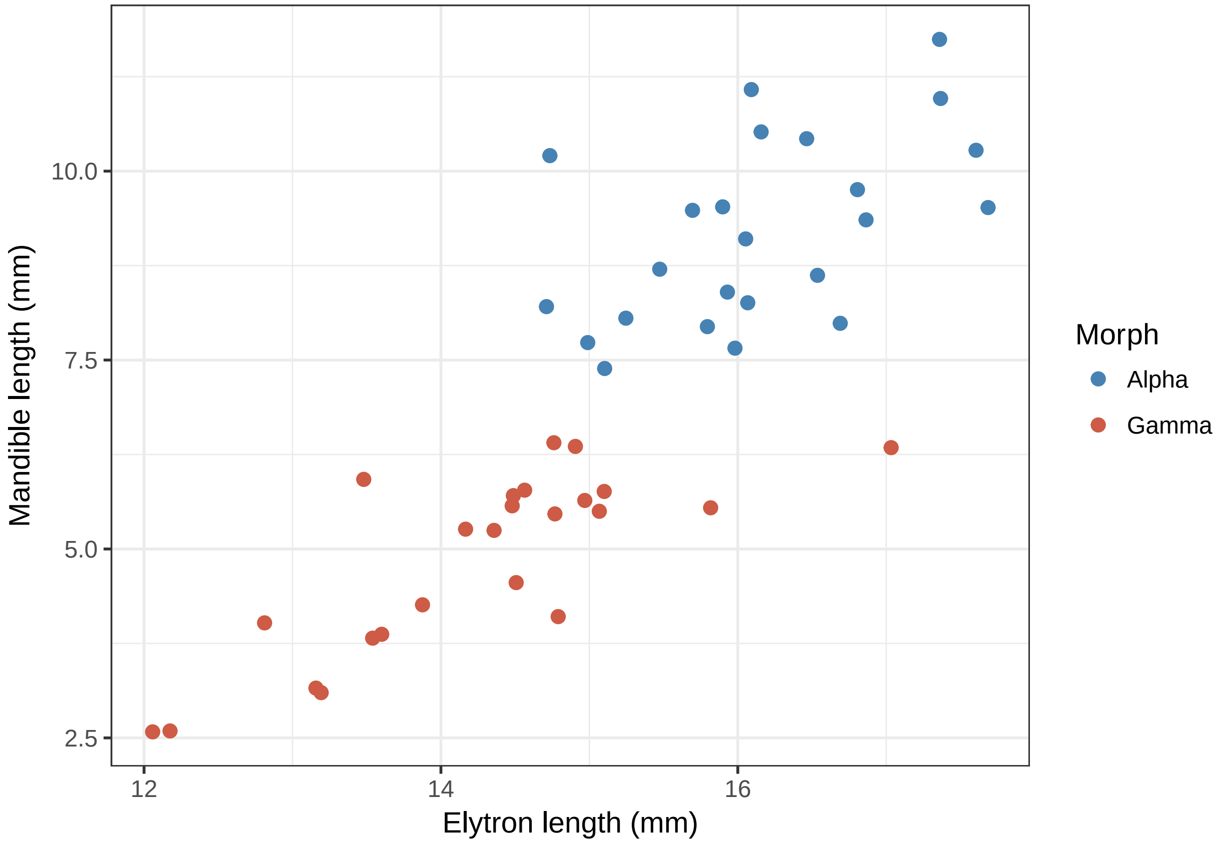

Figure S19 Mandible length against Elytron length with the morph allocation from the mixture model shown for *O platynota*

Model fitting for *O. platynota*

Comparison of candidate models explaining the variation in mandible length.

mod1: body size (elytra length) only
mod2: body size and morph, same slopes (no interaction)
mod3: body size and morph, different slopes

```
mod1 <- lm(log(left_mandible_straight) ~ log(elytra_middle),
    data = platy)
mod2 <- lm(log(left_mandible_straight) ~ log(elytra_middle) +
    Morph, data = platy)
mod3 <- lm(log(left_mandible_straight) ~ log(elytra_middle) *
    Morph, data = platy)

AIC(mod1, mod2, mod3)
```

```
##      df       AIC
## mod1  3 -15.91425
## mod2  4 -42.77186
## mod3  5 -46.94247
```

The model with two morphs and different slopes has a much lower AIC than the other models and so we accept this as the best one. The Gamma morphs have a lower intercept and a higher slope relating log elytron length to mandible length than the Alpha morphs.

```r
platy_mod <- mod3

summary(mod3)
```

```
##
## Call:
## lm(formula = log(left_mandible_straight) ~ log(elytra_middle) *
##     Morph, data = platy)
##
## Residuals:
##      Min       1Q   Median       3Q      Max
## -0.25393 -0.09419  0.00286  0.10738  0.38303
##
## Coefficients:
##                              Estimate Std. Error t value Pr(>|t|)
## (Intercept)                   -1.4105     1.5136  -0.932    0.356
## log(elytra_middle)             1.3029     0.5444   2.393    0.021 *
## MorphGamma                    -4.7854     1.8024  -2.655    0.011 *
## log(elytra_middle):MorphGamma  1.6155     0.6575   2.457    0.018 *
## ---
## Signif. codes:  0 '***' 0.001 '**' 0.01 '*' 0.05 '.' 0.1 ' ' 1
##
## Residual standard error: 0.1397 on 44 degrees of freedom
## Multiple R-squared:  0.8856, Adjusted R-squared:  0.8778
## F-statistic: 113.5 on 3 and 44 DF,  p-value: < 2.2e-16
```

```r
pred <- predict(platy_mod)  #predicted values from model

platy2 <- cbind(platy, pred)


platy_plot1 <- ggplot(data = platy2, aes(x = log(elytra_middle),
    y = log(left_mandible_straight), colour = Morph)) + geom_point(size = 2) +
    scale_colour_manual(values = palette3) + theme_bw() + xlab("Log elytron length (mm)") +
    ylab("Log mandible length (mm)")

platy_plot1 <- platy_plot1 + geom_line(aes(y = pred), size = 0.33) +
    ggtitle("O. platynota") + theme(plot.title = element_text(face = "italic"))

platy_plot1
```

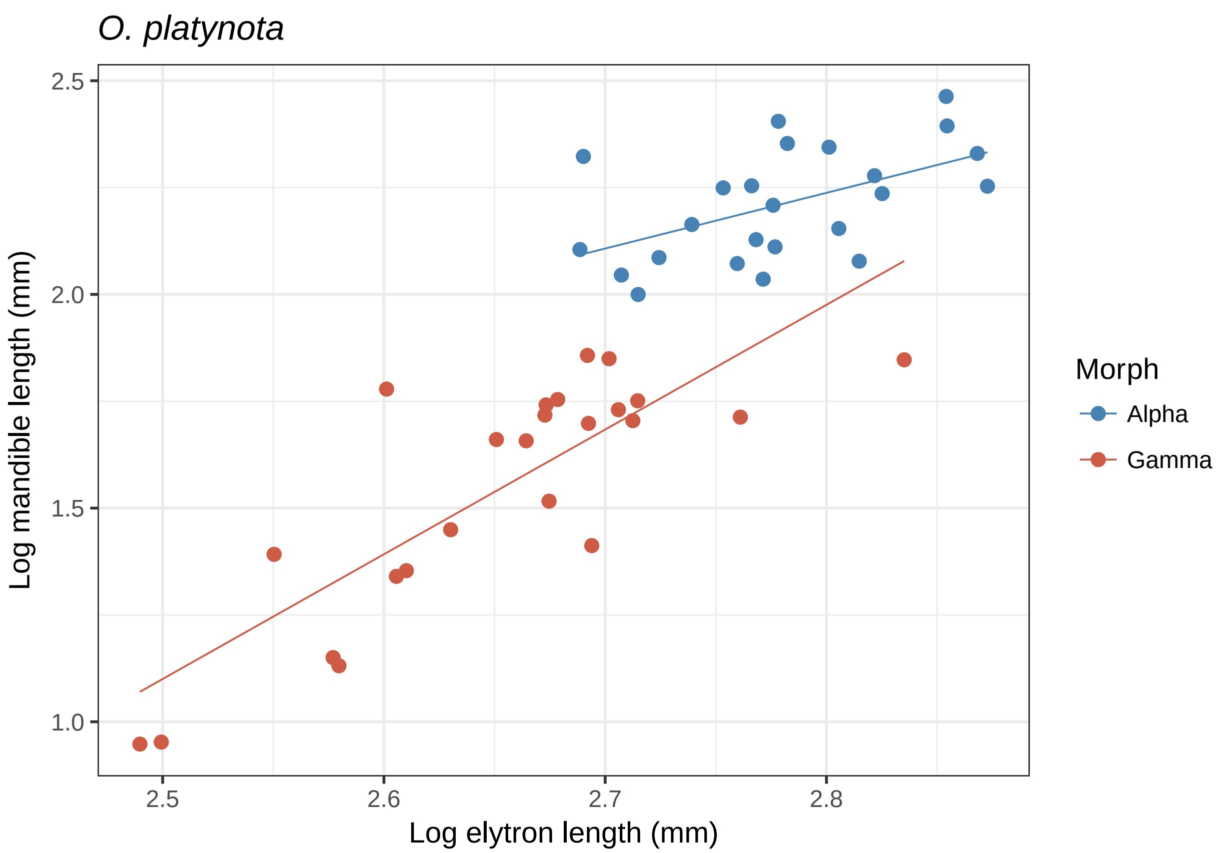

Figure S20 Final allometric plot for *O. platynota* with morph allocations and lines from the fitted model.

Morphometrics for *O. platynota*

```
s = platy %>% split(platy$Morph)

ch = s %>% # Compute which points are on the convex hull of each
# data.frame /!\ Check how 'chull' works
lapply(., function(el) chull(el$pc1, el$pc2))  # 'ch' now contains the
# row numbers of points on convex hull per sub-data.frame

# Get points for each sub-data.frame using names index
ch = lapply(names(ch), function(el) s[[el]][ch[[el]], ]) %>%
    do.call(rbind, .)  # Join all convex hull points in a single data.frame


# p1<-ggplot(data = platy, aes(x = pc1, y = pc2, colour =
# Morph)) + geom_point(size = 2) +
# scale_colour_brewer(palette = 'Set1') +
# scale_fill_brewer(palette = 'Set1') + theme_bw() +
# xlab('Principal component 1') + ylab('Principal component
# 2')

platynota_pca_plot <- ggplot(data = platy, aes(x = pc1, y = pc2,
    colour = Morph)) + geom_point(size = 2) + scale_colour_manual(values = palette3) +
```

```
    scale_fill_manual(values = palette3) + theme_bw() + xlab("Principal component 1") +
    ylab("Principal component 2")


platynota_pca_plot <- platynota_pca_plot + geom_polygon(data = ch,
    aes(fill = Morph, colour = NA), alpha = 0.2) + ggtitle("O. platynota") +
    theme(plot.title = element_text(face = "italic"))


platynota_pca_plot
```

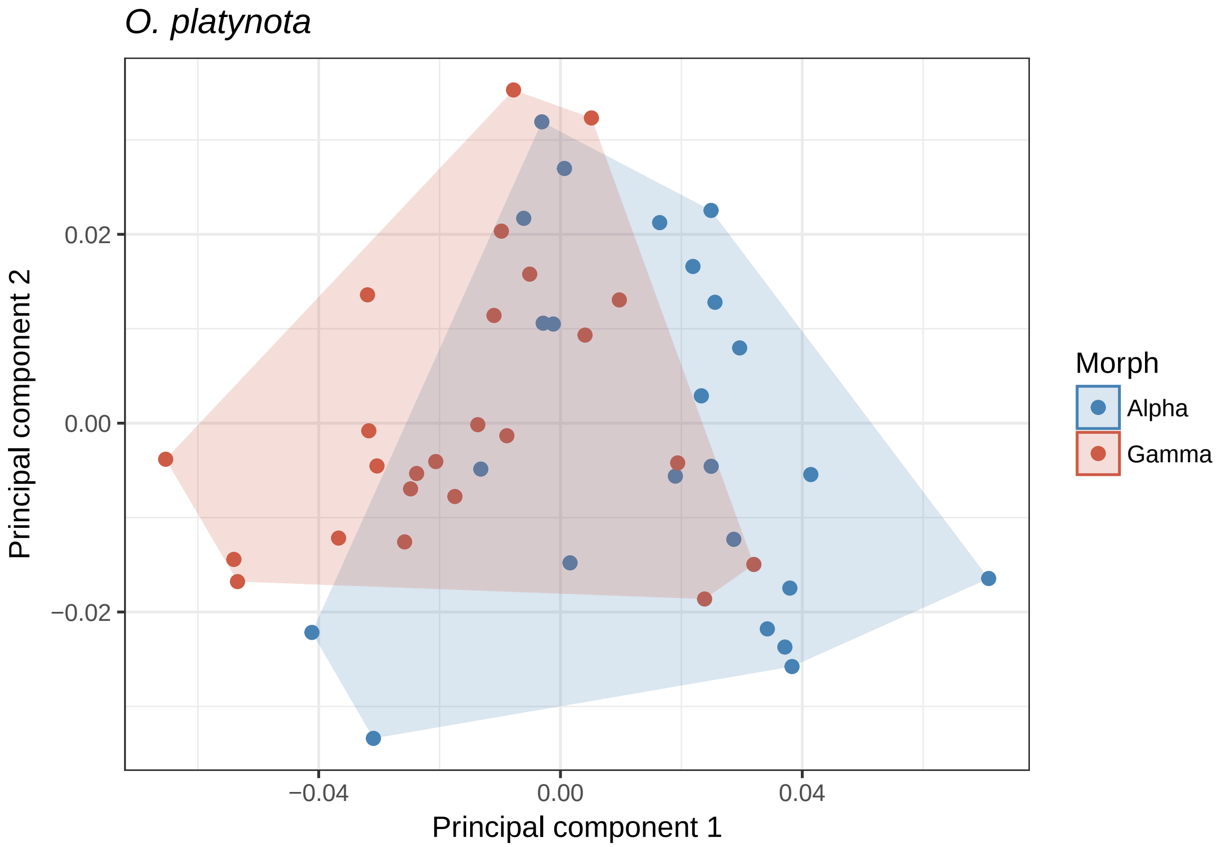

Figure S21 PC plot from relative warp analysis for *O. platynota*

Note the strong indication of a 'horseshoe' or 'arch' effect in this plot. These arise when there is one overridingly important gradient and different groups have nonlinear distributions along it - in this case the Alphas are at one end and the Gammas are at the other. Nonetheless, the two ends are reasonably well associated with high or low values of PC1. Examination of relative warps suggests that this gradient of shapes is largely associated with larger heads relative to prothoraxes, and wider fronts of the head, for more positive values of PC1.

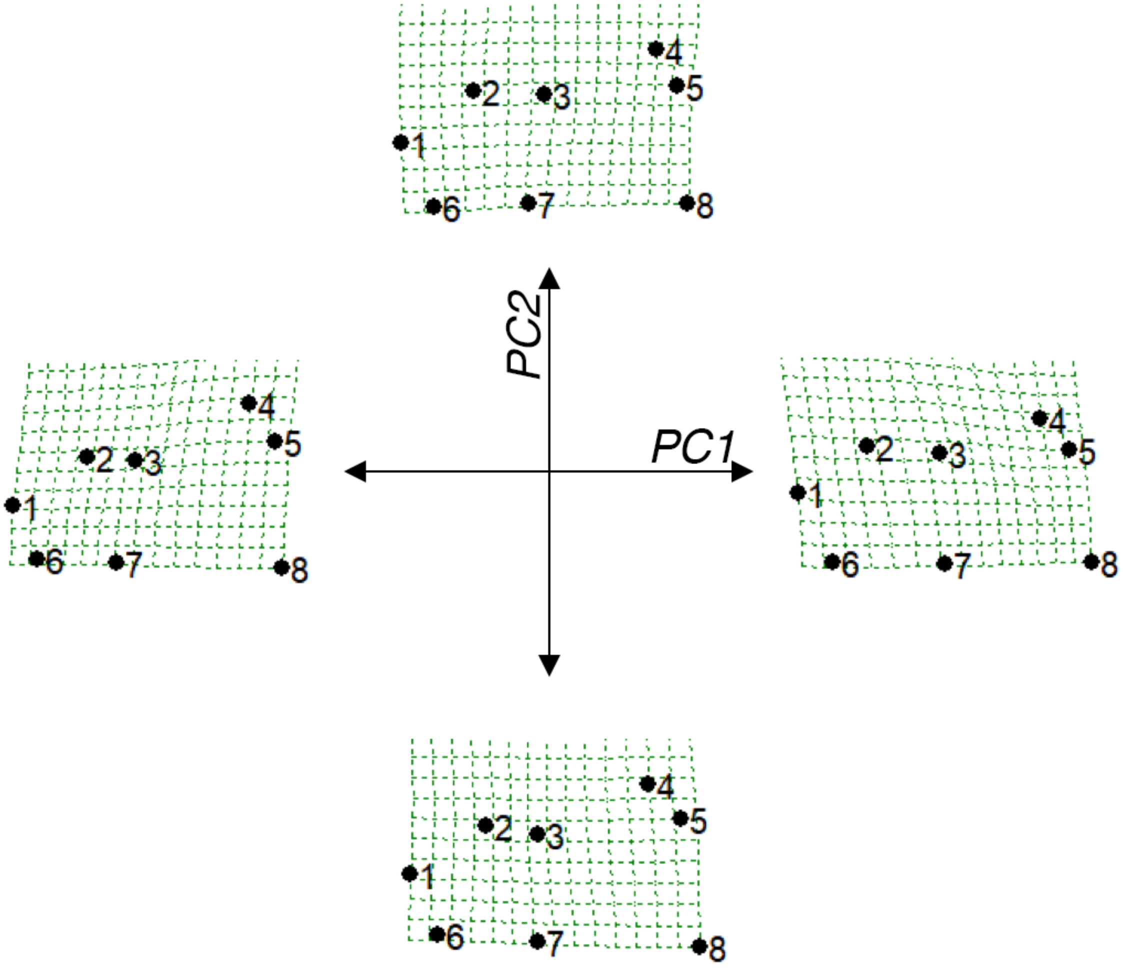

Figure S22 Thin plate splines showing how head and pronotum shape vary along the first two principal components for *O. platynota*

```
p1 <- ggplot(data = platy, aes(x = elytra_middle, y = pc1, colour = Morph)) +
    scale_colour_manual(values = palette3) + geom_point(size = 2) +
    theme_bw() + ylab("Principal component 1") + xlab("Elytron length (mm")

p1
```

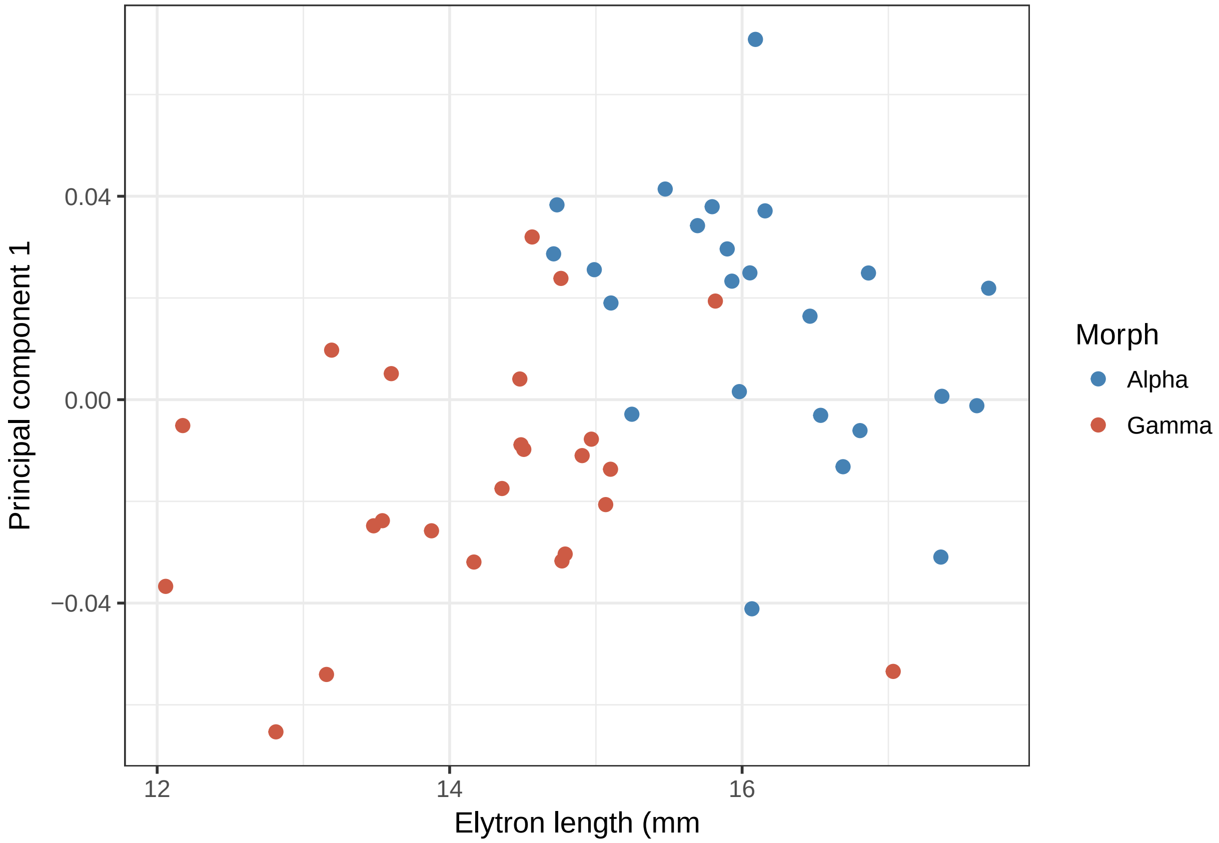

Figure S23 PC1 from the relative warp analysis plotted against body size, as indicated by elytron length.

Model comparison

mod1: body size (elytra length) only
mod2: two morph model, same slopes (no interaction)
mod3: two morph model, different slopes

```
mod1 <- lm(pc1 ~ elytra_middle, data = platy)
mod2 <- lm(pc1 ~ elytra_middle + Morph, data = platy)
mod3 <- lm(pc1 ~ elytra_middle * Morph, data = platy)

AIC(mod1, mod2, mod3)
```

```
##      df       AIC
## mod1  3 -203.5322
## mod2  4 -213.6477
## mod3  5 -216.2172
```

mod3 has the lowest AIC score and the difference between mod3 and mod2 is 2.6 so we cautiously accept the two morph, different slopes model as the best.

```
summary(mod3)
```

```
##
## Call:
## lm(formula = pc1 ~ elytra_middle * Morph, data = platy)
##
## Residuals:
##       Min        1Q    Median        3Q       Max
```

```
## -0.057714 -0.013919  0.000044  0.017679  0.054540
##
## Coefficients:
##                          Estimate Std. Error t value Pr(>|t|)
## (Intercept)               0.207996   0.093366   2.228   0.0311 *
## elytra_middle            -0.011913   0.005778  -2.062   0.0452 *
## MorphGamma               -0.272031   0.113039  -2.407   0.0204 *
## elytra_middle:MorphGamma  0.015305   0.007301   2.096   0.0418 *
## ---
## Signif. codes:  0 '***' 0.001 '**' 0.01 '*' 0.05 '.' 0.1 ' ' 1
##
## Residual standard error: 0.02395 on 44 degrees of freedom
## Multiple R-squared:  0.3677, Adjusted R-squared:  0.3246
## F-statistic: 8.531 on 3 and 44 DF,  p-value: 0.0001409
```

PC1 is correlated with size but only weakly and although the Alphas cluster towards the positive values of PC1 the pattern is not especially clear. Gamma morphs have higher values of PC1 on average but PC1 does not vary with body size, whereas PC1 increases with body size in Alpha morphs.

O. siva

Males of *O. siva* are clearly dimorphic, with well-separated groups visible in a scatterplot of mandible length against elytron length. There are Alpha males, with long and exaggerated mandibles, and Gamma males with short mandibles resembling those of females. Unlike *O. platynota*, where there appears to be a threshold size above which males develop into Alphas, in *O. siva* there is considerable overlap between the two morphs: below an elytron length of about 28mm all males develop into Gammas, but above this size the majority still develop into gammas, right up to the largest body sizes - indeed, the largest beetle in the sample is a gamma morph. As with *O.platynota*, allocation of individuals to morphs via a mixture model with two clusters corresponds with visual allocation of these animals.

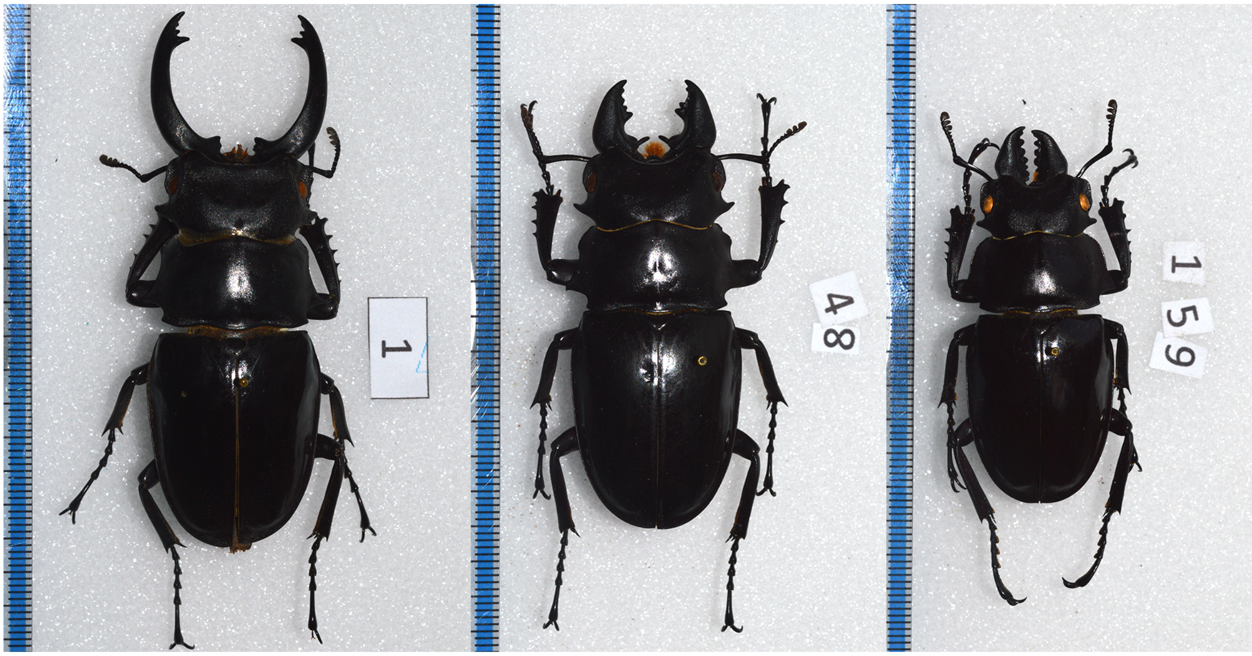

Figure S24 Examples of the different morphs for *O. siva*

```
siva <- subset(alldata, species == "siva")

siva$pc1 <- -siva$pc1  #Reverse sign for PC1 to make it comparable to other analyses



siva$morph_visual <- factor(ifelse(siva$morph_visual == "X-morph",
    "Alpha", as.character(siva$morph_visual)))  #Reclassify Keita's 'X-morph' as an alpha


# Set up new variable for ratio
ratio <- siva$left_mandible_straight/siva$elytra_middle


# Fit mixture model

mix.mod <- flexmix(ratio ~ 1, k = 2, cluster = as.numeric(droplevels(siva$morph_visual)))


#### plot histogram

x1 <- seq(min(ratio), max(ratio), length = 100)

counts <- table(clusters(mix.mod))

d1 <- dnorm(x1, parameters(mix.mod)[1, 1], parameters(mix.mod)[2,
```

```
    1]) * counts[1]/sum(counts)
d2 <- dnorm(x1, parameters(mix.mod)[1, 2], parameters(mix.mod)[2,
    2]) * counts[2]/sum(counts)


# densities <- data.frame(x1, d1, d2, d3)
densities <- data.frame(x1, d1, d2)

p1 <- ggplot(data = data.frame(ratio), aes(ratio, ..density..)) +
    geom_histogram(fill = "grey80", colour = "black", bins = 16) +
    theme_bw() + xlab("Mandible length / elytron length")

p1 <- p1 + geom_line(data = densities, aes(x = x1, y = d1))
p1 <- p1 + geom_line(data = densities, aes(x = x1, y = d2))

p1
```

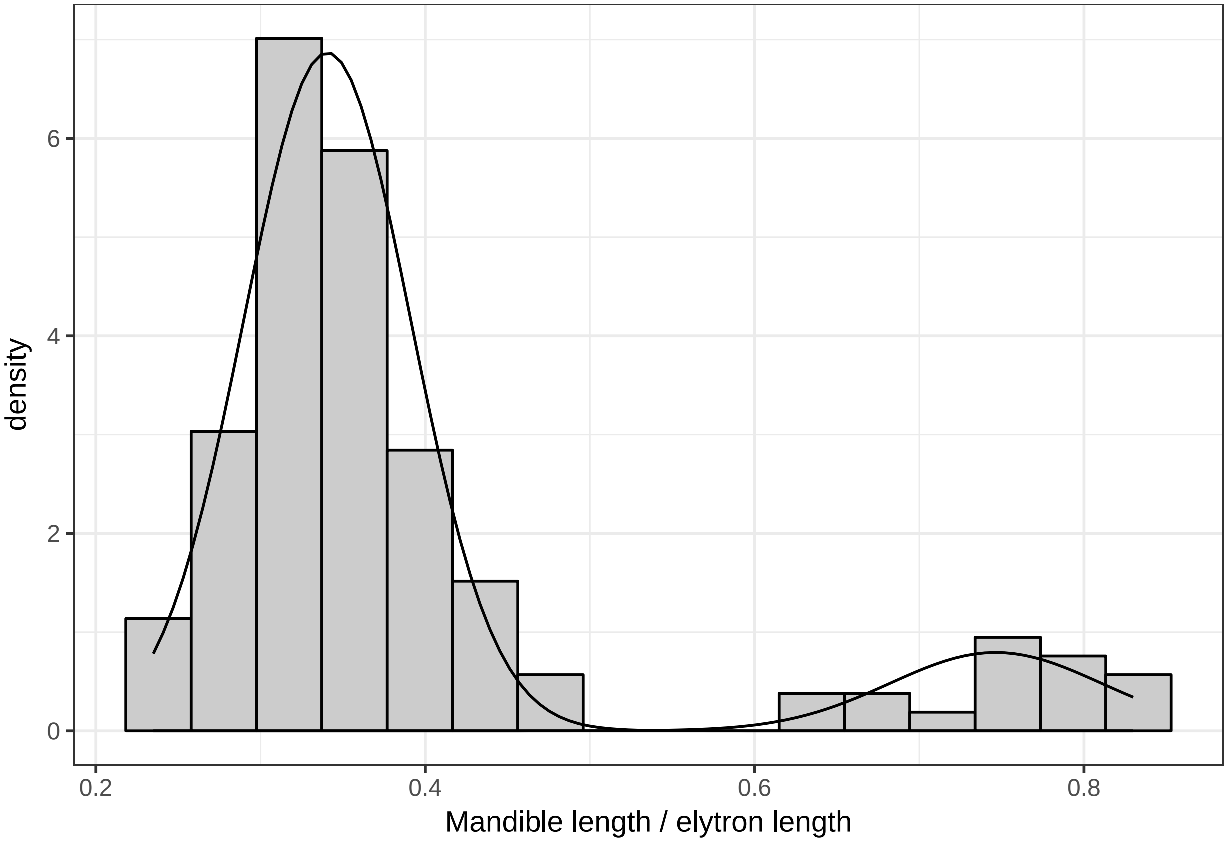

Figure S25 Histogram showing the frequency distribution of ratios of mandible length to elytra length for *O. siva* with the two fitted normal distributions from the mixture model.

```
Morph <- ifelse(clusters(mix.mod) == 1, "Alpha", "Gamma")

siva <- data.frame(siva, Morph)

Siva_Morph <- Morph  #save morph allocations for plotting proportions later on

rm(Morph)
```

```r
ggplot(data = siva, aes(x = elytra_middle, y = left_mandible_straight,
    colour = Morph)) + geom_point(size = 2) + scale_colour_manual(values = palette3) +
    theme_bw() + xlab("Elytron length (mm)") + ylab("Mandible length")
```

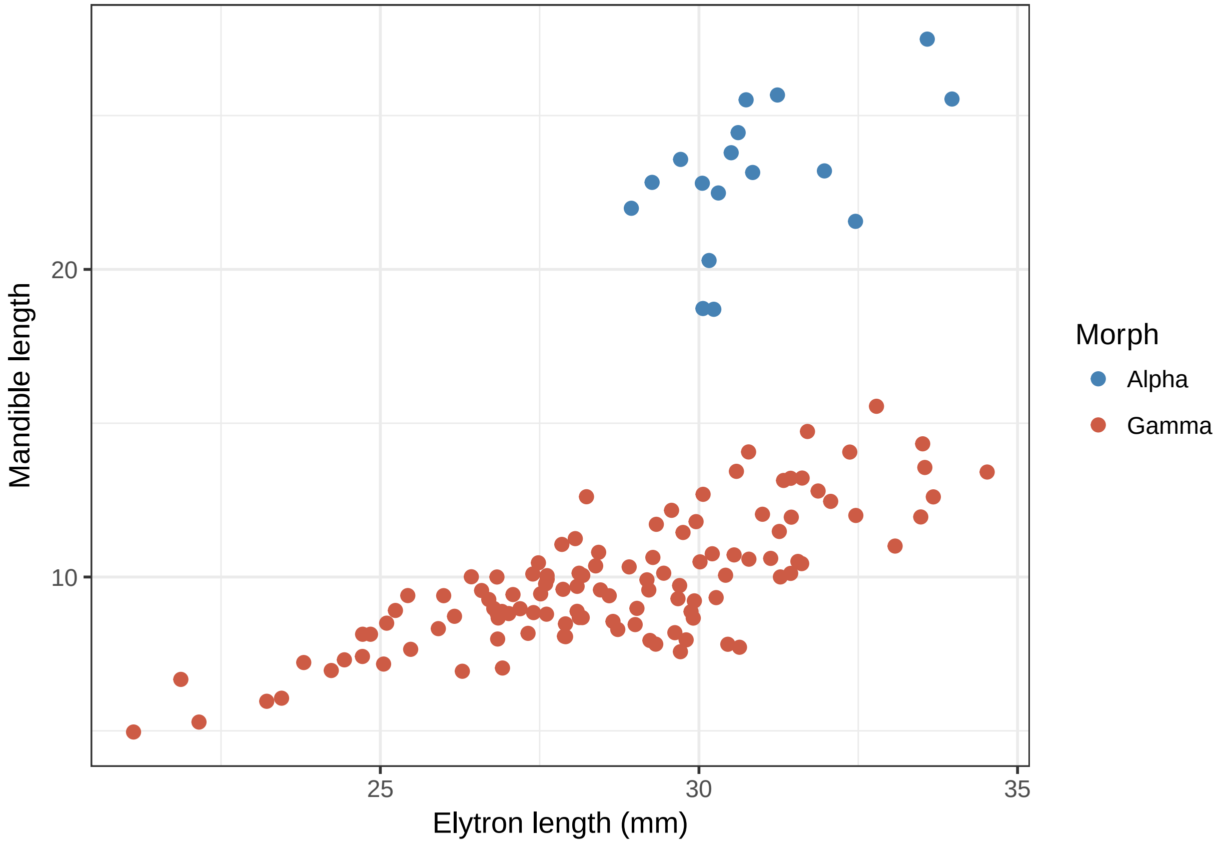

Figure S26 Mandible length against Elytron length with the morph allocation from the mixture model shown for *O siva*

Model fitting for *O. siva*

Comparison of candidate models explaining the variation in mandible length.

mod1: two morph, same slopes (no interaction)
mod2: two morph, different slopes mod3: body size (elytra length) only

```r
mod1 <- lm(log(left_mandible_straight) ~ log(elytra_middle) +
    Morph, data = siva)
mod2 <- lm(log(left_mandible_straight) ~ log(elytra_middle) *
    Morph, data = siva)
mod3 <- lm(log(left_mandible_straight) ~ log(elytra_middle),
    data = siva)


AIC(mod1, mod2, mod3)
```

```
##      df        AIC
## mod1  4 -166.62226
## mod2  5 -165.25532
```

```
## mod3  3   31.01341
```

On the basis of AIC the two-morph models are much better supported than the model based on body size alone, but it is not possible to distinguish between the two because their AIC values are almost identical, so we cannot say with confidence whether the slopes of the allometric relationships vary between the morphs.

```
siva_mod <- mod1

pred <- predict(siva_mod)  #predicted values from model

siva2 <- cbind(siva, pred)


siva_plot1 <- ggplot(data = siva2, aes(x = log(elytra_middle),
    y = log(left_mandible_straight), colour = Morph)) + geom_point(size = 2) +
    scale_colour_manual(values = palette3) + theme_bw() + xlab("Log elytron length (mm)") +
    ylab("Log mandible length (mm)")

siva_plot1 <- siva_plot1 + geom_line(aes(y = pred), size = 0.33) +
    ggtitle("O. siva") + theme(plot.title = element_text(face = "italic"))

siva_plot1
```

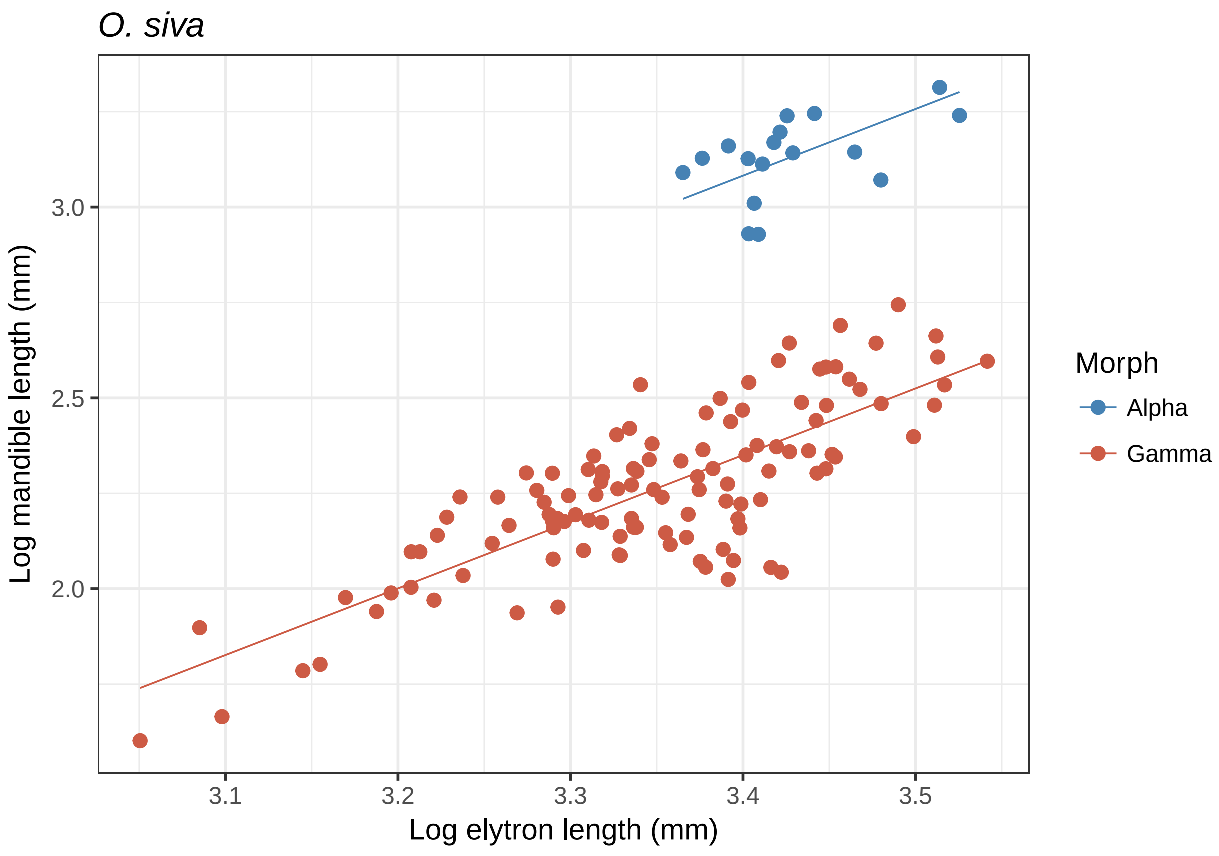

Figure S27 Final allometric plot for *O. siva* with morph allocations and lines from the fitted model.

Morphometrics for *O. siva*

```r
s = siva %>% split(siva$Morph)

ch = s %>% # Compute which points are on the convex hull of each
# data.frame /!\ Check how 'chull' works
lapply(., function(el) chull(el$pc1, el$pc2))  # 'ch' now contains the
# row numbers of points on convex hull per sub-data.frame

# Get points for each sub-data.frame using names index
ch = lapply(names(ch), function(el) s[[el]][ch[[el]], ]) %>%
    do.call(rbind, .)  # Join all convex hull points in a single data.frame


siva_pca_plot <- ggplot(data = siva, aes(x = pc1, y = pc2, colour = Morph)) +
    geom_point(size = 2) + scale_colour_manual(values = palette3) +
    scale_fill_manual(values = palette3) + theme_bw() + xlab("Principal component 1") +
    ylab("Principal component 2")


siva_pca_plot <- siva_pca_plot + geom_polygon(data = ch, aes(fill = Morph,
    colour = NA), alpha = 0.2) + ggtitle("O. siva") + theme(plot.title = element_text(face = "italic"))


siva_pca_plot
```

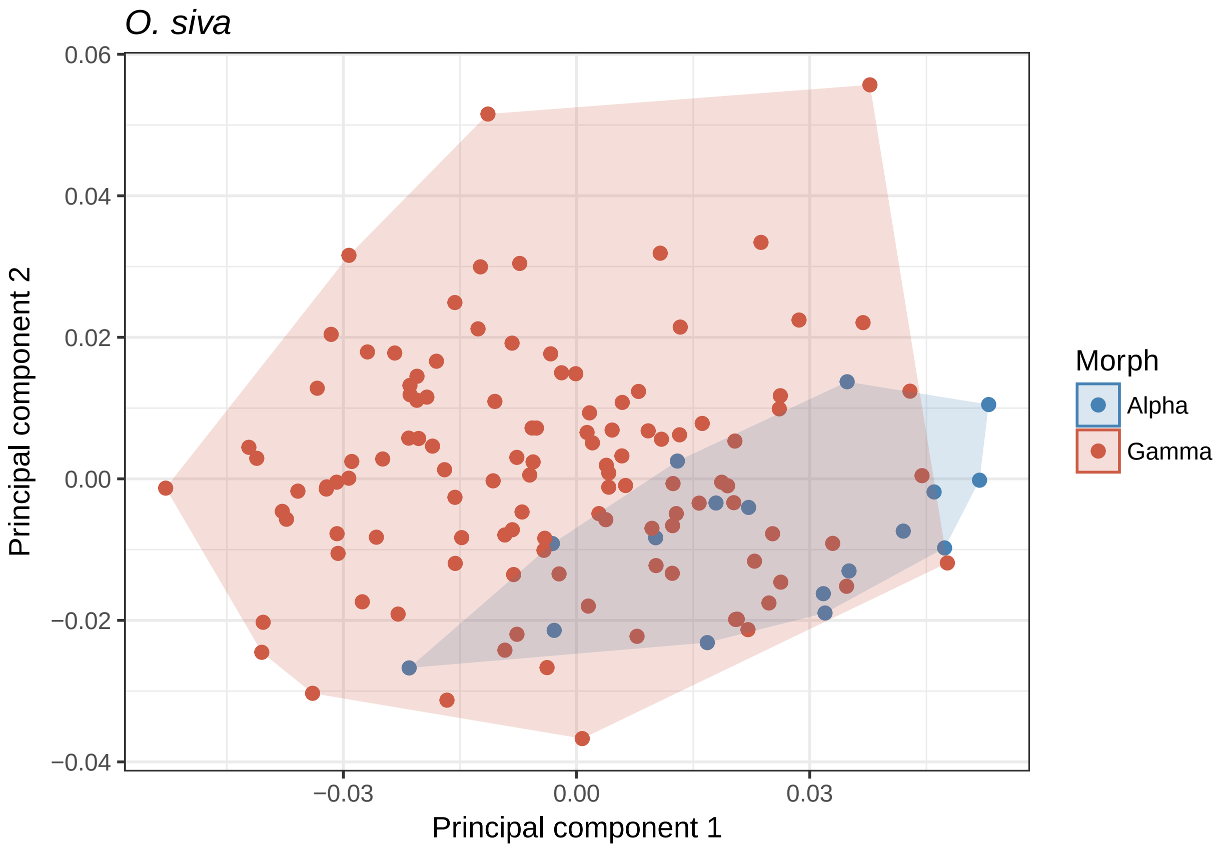

Figure S28 PC plot from relative warp analysis for *O. siva*

As can be seen there is considerable overlap between the morphs - they are not distinguishable on the basis of either PC1 or PC2 - although the Alphas do tend to cluster in the bottom right hand corner of the plot there is no indication of the clear separation of the 'large mandible' morphs which we have seen in other species.

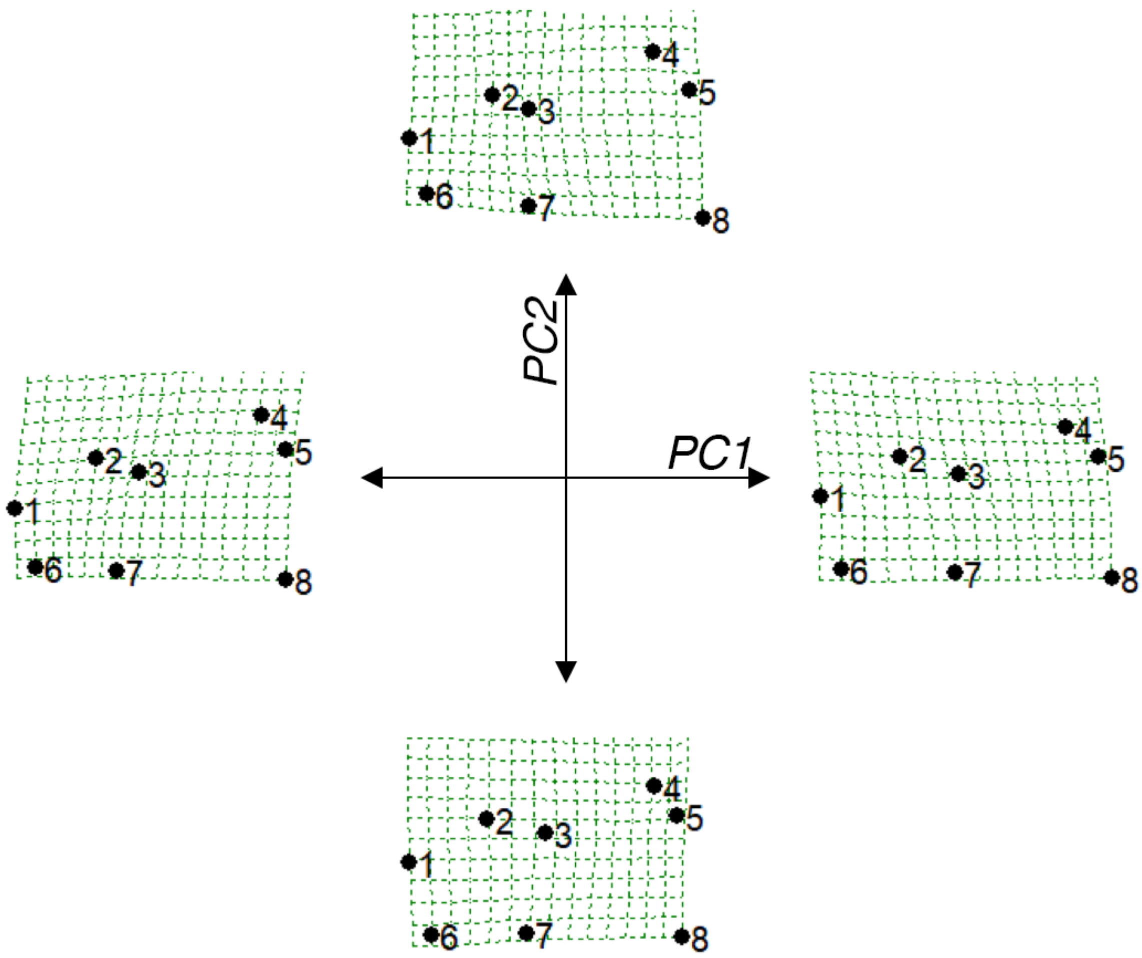

Figure S29 Thin plate splines showing how head and pronotum shape vary along the first two principal components for *O. siva*

Positive values of PC1 are associated with a wider front of the head and a relatively larger head, whereas PC2 is associated with the prothorax narrowing slightly and the distance between the eye (landmark 2) and the front of the prothorax being reduced.

Relation with body size - because PC2 is more important here than in the other analyses we'll look at both PC1 and PC2

```
library(cowplot)

p1 <- ggplot(data = siva, aes(x = elytra_middle, y = pc1, colour = Morph)) +
    scale_colour_manual(values = palette3) + geom_point(size = 2) +
    theme_bw() + ylab("Principal component 1") + xlab("Elytron length (mm")

p2 <- ggplot(data = siva, aes(x = elytra_middle, y = pc2, colour = Morph)) +
```

```
    scale_colour_manual(values = palette3) + geom_point(size = 2) +
    theme_bw() + ylab("Principal component 2") + xlab("Elytron length (mm")

plot_grid(p1, p2)
```

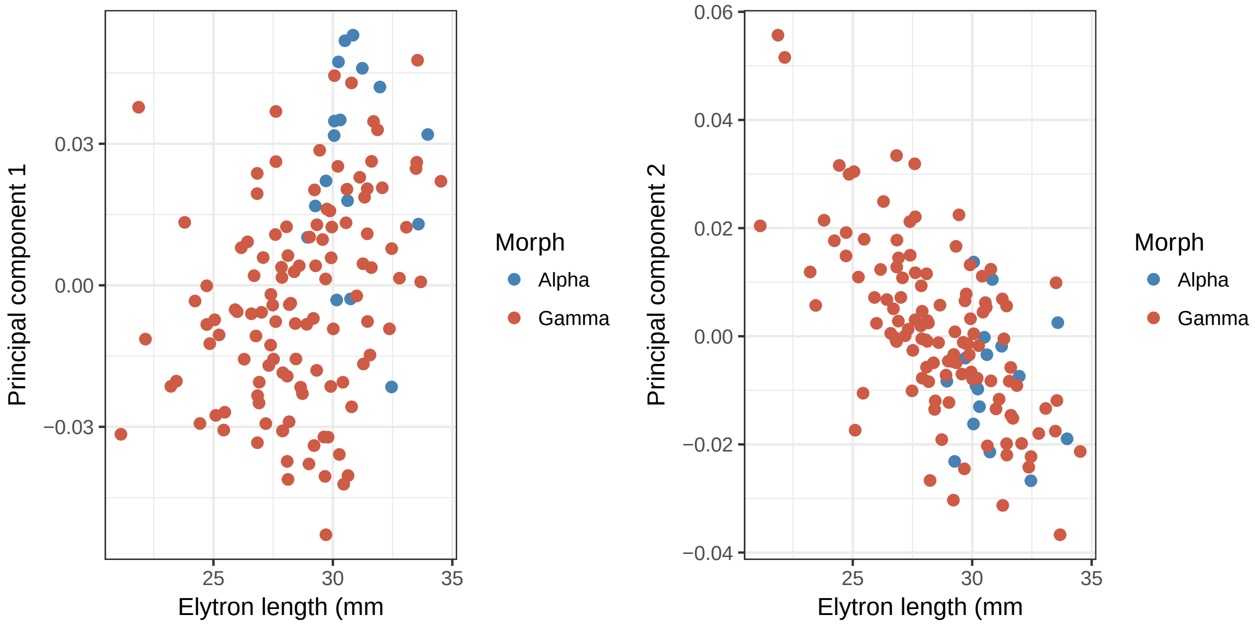

Figure S30 PC1 and PC2 from relative warp analysis plotted against body size (Elytron length)

Both PC1 and PC2 are strongly related to body size - large beetles have relatively large, squarer heads with realtively bigger distances between the eyes and the prothoraxes. Morph seems to be unrelated to shape - the clustering of Alphas on the original PCA plot is simply because Apha morphs tend to be larger.

Testing some models for the relationship between morph, body size and pc1 / pc2

pc1:

mod1: two morph, different slopes mod2: two morph, same slopes mod3: one morph (only elytron length as an explanatory variable)

pc2:

mod4: two morph, different slopes mod5: two morph, same slopes mod6: one morph (only elytron length as an explanatory variable)

```
mod1 <- lm(pc1 ~ Morph * elytra_middle, data = siva)
mod2 <- lm(pc1 ~ Morph + elytra_middle, data = siva)
mod3 <- lm(pc1 ~ elytra_middle, data = siva)


mod4 <- lm(pc2 ~ Morph * elytra_middle, data = siva)
mod5 <- lm(pc2 ~ Morph + elytra_middle, data = siva)
mod6 <- lm(pc2 ~ elytra_middle, data = siva)

AIC(mod1, mod2, mod3)
```

```
##      df       AIC
## mod1  5 -643.6430
## mod2  4 -644.7891
```

```
## mod3  3 -630.6013
```

```r
AIC(mod4, mod5, mod6)
```

```
##      df       AIC
## mod4  5 -791.2015
## mod5  4 -790.9107
## mod6  3 -792.8883
```

For PC1 the two morph models are preferred, although we can't distinguish between the same and different slopes models. For PC2 the two morph same slopes model and the one morph model are roughly smilar in explanatory power.

```r
summary(mod2)
```

```
##
## Call:
## lm(formula = pc1 ~ Morph + elytra_middle, data = siva)
##
## Residuals:
##       Min        1Q    Median        3Q       Max
## -0.051833 -0.015652  0.001627  0.014356  0.057166
##
## Coefficients:
##                 Estimate Std. Error t value Pr(>|t|)
## (Intercept)   -0.0473276  0.0229150  -2.065  0.04088 *
## MorphGamma    -0.0234045  0.0057057  -4.102 7.18e-05 ***
## elytra_middle  0.0023460  0.0007239   3.241  0.00151 **
## ---
## Signif. codes:  0 '***' 0.001 '**' 0.01 '*' 0.05 '.' 0.1 ' ' 1
##
## Residual standard error: 0.02103 on 130 degrees of freedom
## Multiple R-squared:  0.2271, Adjusted R-squared:  0.2152
## F-statistic:  19.1 on 2 and 130 DF,  p-value: 5.348e-08
```

So when we take body size into account Alphas do have slightly higher scores on PC1, so there are some morphological differences. These are small by comparison with the other species, however.

O. sommeri lowei

O. sommeri was initially compiled with both *O. sommeri s. stricto* and *O. sommeri lowei* included in the same data set, but initial analysis indicated that there were some important differences in allometry between the two and so they are analysed separately.

O. sommeri lowei have four distinguishable morphs: Alphas, Betas and Gammas which correspond morphologically to the three morphs distinguished by Rowland and Emlen in *O. cuvera* and a fourth morph of "Boltcutter" males with short, robust mandibles. These can be distinguished visually and also group separately on a scatterplot of mandible length versus elytron length.

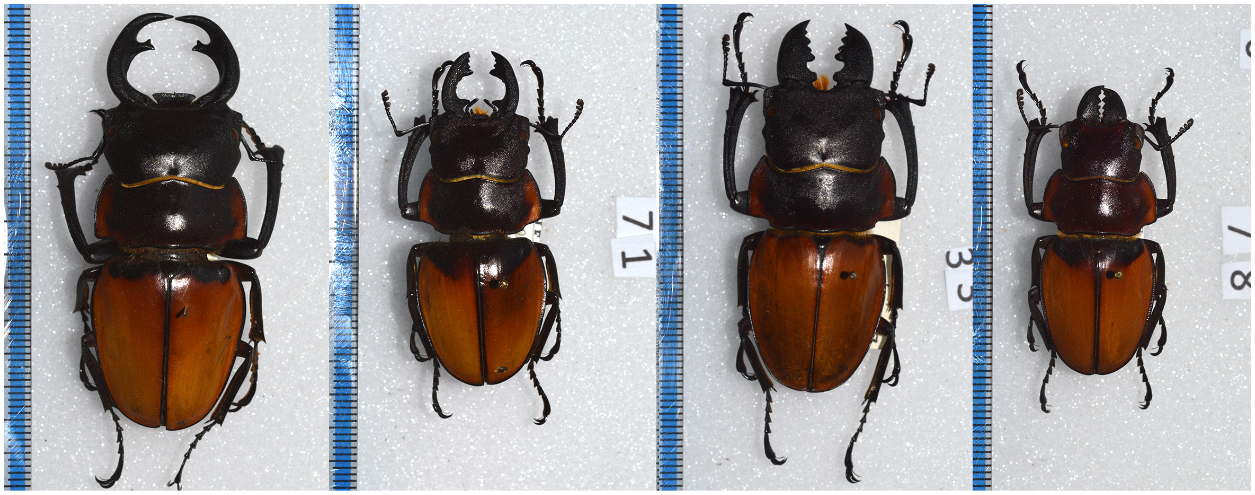

Figure S31 Examples of the different morphs for *O. someri lowei*

A mixture model with four clusters fitted to the ratio of mandible length to elytron length ratios is useful for separating the alpha morphs and the gamma morphs but because the lengths of the Beta and Boltcutter mandibles overlap the mixture model is not useful for separating these animals.

```
lowei <- subset(alldata, species == "lowei")

lowei$pc2 <- -lowei$pc2  #reverse values for pc2 to make it equivalent to the other species


# lowei2<-subset(lowei, morph_visual == 'Gamma' |
# morph_visual == 'Boltcutter') ggplot(data = lowei,
# aes(left_mandible_straight/elytra_middle))+geom_histogram(aes(y=..density..),
# fill = 'grey50', colour = 'black', bins =
# 20)+theme_bw()+geom_density(bw = 0.02) + ggtitle('Histogram
# of mandible to elytron ratios for the full dataset')


# Set up new variable for ratio
ratio <- lowei$left_mandible_straight/lowei$elytra_middle

# Gaussian models specified for mixture model (model will
# only converge with separate models specified)
g1 <- FLXMRglm(family = "gaussian")
g2 <- FLXMRglm(family = "gaussian")
g3 <- FLXMRglm(family = "gaussian")
g4 <- FLXMRglm(family = "gaussian")


# Fit mixture model

mix.mod <- flexmix(ratio ~ 1, k = 4, model = list(g1, g2, g3,
    g4), cluster = as.numeric(lowei$morph_visual))
```

```r
# mix.mod<-flexmix(ratio~1, k = 3)


#### plot histogram

x1 <- seq(min(ratio), max(ratio), length = 100)

counts <- table(clusters(mix.mod))

d1 <- dnorm(x1, parameters(mix.mod)[[1]][1, 1], parameters(mix.mod)[[1]][2,
    1]) * counts[1]/sum(counts)
d2 <- dnorm(x1, parameters(mix.mod)[[1]][1, 2], parameters(mix.mod)[[1]][2,
    2]) * counts[2]/sum(counts)
d3 <- dnorm(x1, parameters(mix.mod)[[1]][1, 3], parameters(mix.mod)[[1]][2,
    3]) * counts[3]/sum(counts)
d4 <- dnorm(x1, parameters(mix.mod)[[1]][1, 4], parameters(mix.mod)[[1]][2,
    4]) * counts[4]/sum(counts)

densities <- data.frame(x1, d1, d2, d3, d4)

p1 <- ggplot(data = data.frame(ratio), aes(ratio, ..density..)) +
    geom_histogram(fill = "grey80", colour = "black", bins = 16) +
    theme_bw() + xlab("Mandible length / elytron length")

p1 <- p1 + geom_line(data = densities, aes(x = x1, y = d1))
p1 <- p1 + geom_line(data = densities, aes(x = x1, y = d2))
p1 <- p1 + geom_line(data = densities, aes(x = x1, y = d3))
p1 <- p1 + geom_line(data = densities, aes(x = x1, y = d4))
p1
```

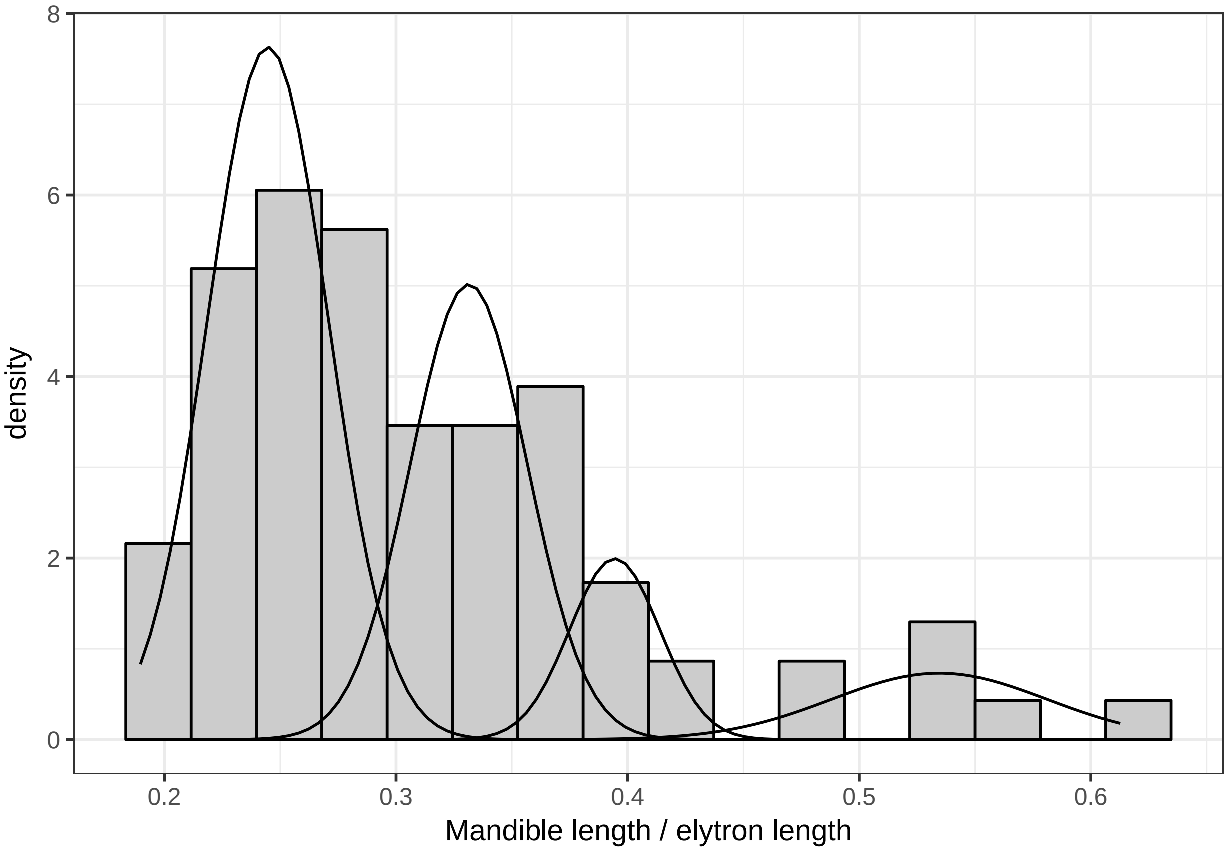

Figure S32 Histogram showing the frequency distribution of ratios of mandible length to elytra length for *O. sommeri lowei*. Fitted normal distributions from the 4-cluster mixture model are shown.

```
### Morph allocation

# Morph <- ifelse
# (lowei$left_mandible_straight/lowei$elytra_middle < 0.3,
# 'Gamma', 'Boltcutter')

Morph <- ifelse(clusters(mix.mod) == 1, "Alpha", "Beta")
Morph <- ifelse(clusters(mix.mod) == 3, "Boltcutter", Morph)
Morph <- ifelse(clusters(mix.mod) == 4, "Gamma", Morph)
# Morph[which(lowei$morph_visual == 'Beta')] <- 'Beta'
# Morph[which(lowei$morph_visual == 'Alpha')] <- 'Alpha'

Morph <- factor(Morph)

# lowei <- cbind (lowei, Morph)

p1 <- ggplot(data = lowei, aes(x = elytra_middle, y = left_mandible_straight,
    colour = Morph, shape = morph_visual))
p1 <- p1 + geom_point(size = 2) + scale_colour_manual(values = palette2) +
    theme_bw() + xlab("Elytron length (mm)") + ylab("Mandible length")

p1
```

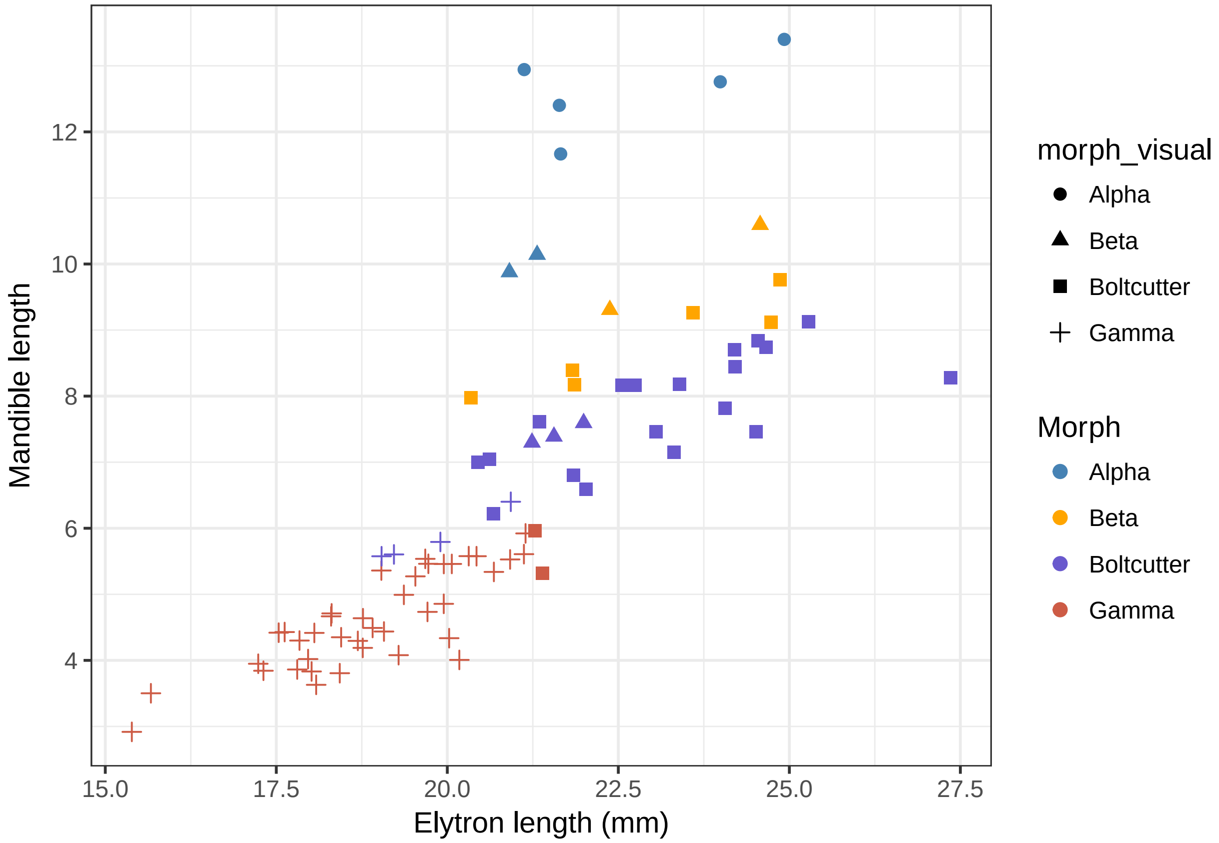

Figure S34 Mandible length against Elytron length with the initial morph allocation shown for *O sommeri lowei*. Shape indicates morph allocations from visual inspection, colour is morph allocation using the mixture model.

The mixture model here does not give an allocation that particularly agrees with the visual allocation, and the classifications differ between the two, particularly for the Beta and Boltcutter morphs. These tend to have similar length mandibles but with very different morphology.

Alphas or Betas?

```
which(Morph == "Alpha" & lowei$morph_visual == "Beta")
```

```
## [1] 53 54
```

Both of these (53 & 54, photos 346 & 351) are betas

Betas or Boltcutters?

```
which(Morph == "Boltcutter" & lowei$morph_visual == "Beta")
```

```
## [1] 21 31 37
```

```
which(Morph == "Beta" & lowei$morph_visual == "Boltcutter")
```

```
## [1] 11 22 23 24 26 82
```

Further examination of some of those which are classified differently or possibly erroneously:

The group of three betas with mandible lengths between 7 and 8:
21 (image 141) is a beta
31 (image 201) is a beta but rather intermediate between the morphs. 37 (image 251) is a beta

Others: 11 (image 76) is a boltcutter 22 (image 146) is a boltcutter 23 (image 156) is a boltcutter 24 (image 161) is a boltcutter 26 (image 171) is a boltcutter 82 (image 531) is a boltcutter

Boltcutters or Gammas?

```
which(Morph == "Boltcutter" & lowei$morph_visual == "Gamma")
```

```
## [1] 41 42 63 76
```

```
which(Morph == "Gamma" & lowei$morph_visual == "Boltcutter")
```

```
## [1] 29 30
```

41 (image 276) is a gamma 42 (image 281) is a gamma 63 (image 411) is a gamma 76 (image 496) is a gamma

29 (image 186) is a gamma 30 (image 191) is a gamma

```
Morph[c(53, 54, 21, 31, 37)] <- "Beta"
Morph[c(11, 22, 23, 24, 26, 82)] <- "Boltcutter"
Morph[c(41, 42, 63, 76, 29, 30)] <- "Gamma"

p1 <- ggplot(data = lowei, aes(x = elytra_middle, y = left_mandible_straight,
    colour = Morph))
p1 <- p1 + geom_point(size = 2) + scale_colour_manual(values = palette2) +
    theme_bw() + xlab("Elytron length (mm)") + ylab("Mandible length")

p1
```

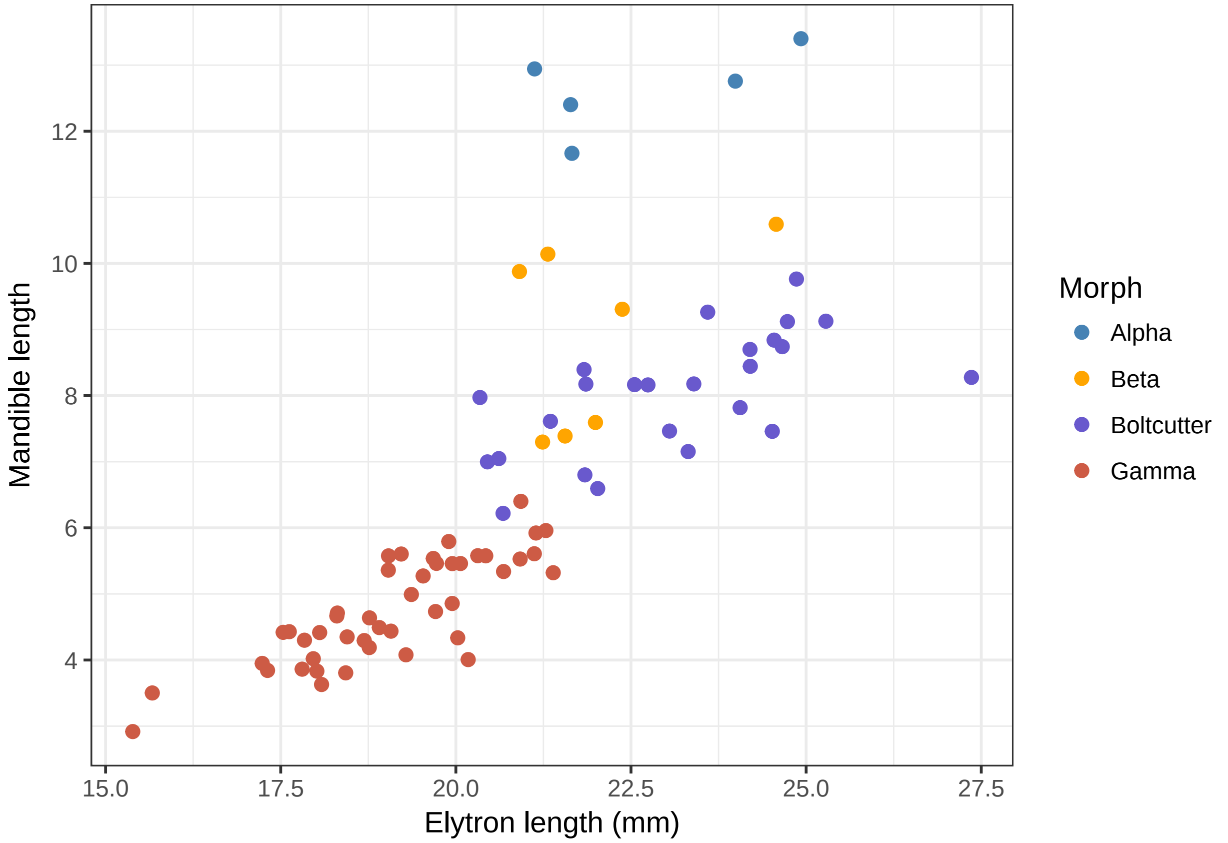

Figure S35 Mandible length against Elytron length with the final morph allocation shown for *O sommeri lowei*

Model fitting for *O. sommeri lowei*

Using two three morph models - one with boltcutters and gammas combined (mods 2 and 4) and one with boltcutters and betas combined (mods 3 and 6) plus two four morph models

mod1: four morph different slopes model
mod2: three morph (Gammas and boltcutters combined) different slopes mod3: three morph (Betas and boltcutters combined) different slopes
mod4: four morph same slopes (no interaction)
mod5: three morph (Gammas and boltcutters combined) same slopes
mod6: three morph (Betas and boltcutters combined) same slopes

```
Morph2 <- factor(ifelse(Morph == "Gamma" | Morph == "Boltcutter",
    "Small", as.character(Morph)))

Morph3 <- factor(ifelse(Morph == "Beta" | Morph == "Boltcutter",
    "Medium", as.character(Morph)))


mod1 <- lm(log(left_mandible_straight) ~ log(elytra_middle) *
    Morph, data = lowei)
mod2 <- lm(log(left_mandible_straight) ~ log(elytra_middle) *
    Morph2, data = lowei)
mod3 <- lm(log(left_mandible_straight) ~ log(elytra_middle) *
    Morph3, data = lowei)
mod4 <- lm(log(left_mandible_straight) ~ log(elytra_middle) +
    Morph, data = lowei)
mod5 <- lm(log(left_mandible_straight) ~ log(elytra_middle) +
    Morph2, data = lowei)
mod6 <- lm(log(left_mandible_straight) ~ log(elytra_middle) +
    Morph3, data = lowei)

AIC(mod1, mod2, mod3, mod4, mod5, mod6)
```

```
##      df       AIC
## mod1  9 -135.7034
## mod2  7 -103.0220
## mod3  7 -128.5410
## mod4  6 -130.4369
## mod5  5 -100.6955
## mod6  5 -118.8271
```

The four morph different slopes model has the lowest AIC and is our preferred model.

```
lowei_mod <- mod1

summary(lowei_mod)
```

```
##
## Call:
## lm(formula = log(left_mandible_straight) ~ log(elytra_middle) *
##     Morph, data = lowei)
##
## Residuals:
##      Min       1Q   Median       3Q      Max
## -0.26985 -0.06080  0.01758  0.06491  0.17945
```

```
##
## Coefficients:
##                                  Estimate Std. Error t value Pr(>|t|)
## (Intercept)                        1.2362     2.1263   0.581   0.5628
## log(elytra_middle)                 0.4166     0.6816   0.611   0.5430
## MorphBeta                         -2.7604     3.1424  -0.878   0.3826
## MorphBoltcutter                   -2.1506     2.2852  -0.941   0.3497
## MorphGamma                        -5.3034     2.2085  -2.401   0.0188 *
## log(elytra_middle):MorphBeta       0.7802     1.0126   0.771   0.4434
## log(elytra_middle):MorphBoltcutter  0.5363     0.7320   0.733   0.4661
## log(elytra_middle):MorphGamma      1.4890     0.7111   2.094   0.0397 *
## ---
## Signif. codes:  0 '***' 0.001 '**' 0.01 '*' 0.05 '.' 0.1 ' ' 1
##
## Residual standard error: 0.09978 on 74 degrees of freedom
## Multiple R-squared:  0.9277, Adjusted R-squared:  0.9208
## F-statistic: 135.5 on 7 and 74 DF,  p-value: < 2.2e-16
```

```r
pred <- predict(lowei_mod)

lowei2 <- lowei[, -25]  #remove the tooth_1_2 measurements



lowei2 <- cbind(lowei2, Morph, pred)

lowei_plot1 <- ggplot(data = lowei2, aes(x = log(elytra_middle),
    y = log(left_mandible_straight), colour = Morph)) + geom_point(size = 2) +
    scale_colour_manual(values = palette2) + theme_bw() + xlab("Log elytron length (mm)") +
    ylab("Log mandible length (mm)")

lowei_plot1 <- lowei_plot1 + geom_line(aes(y = pred), size = 0.33)

lowei_plot1 <- lowei_plot1 + ggtitle("O. sommeri lowei") + theme(plot.title = element_text(face = "itali

lowei_plot1
```

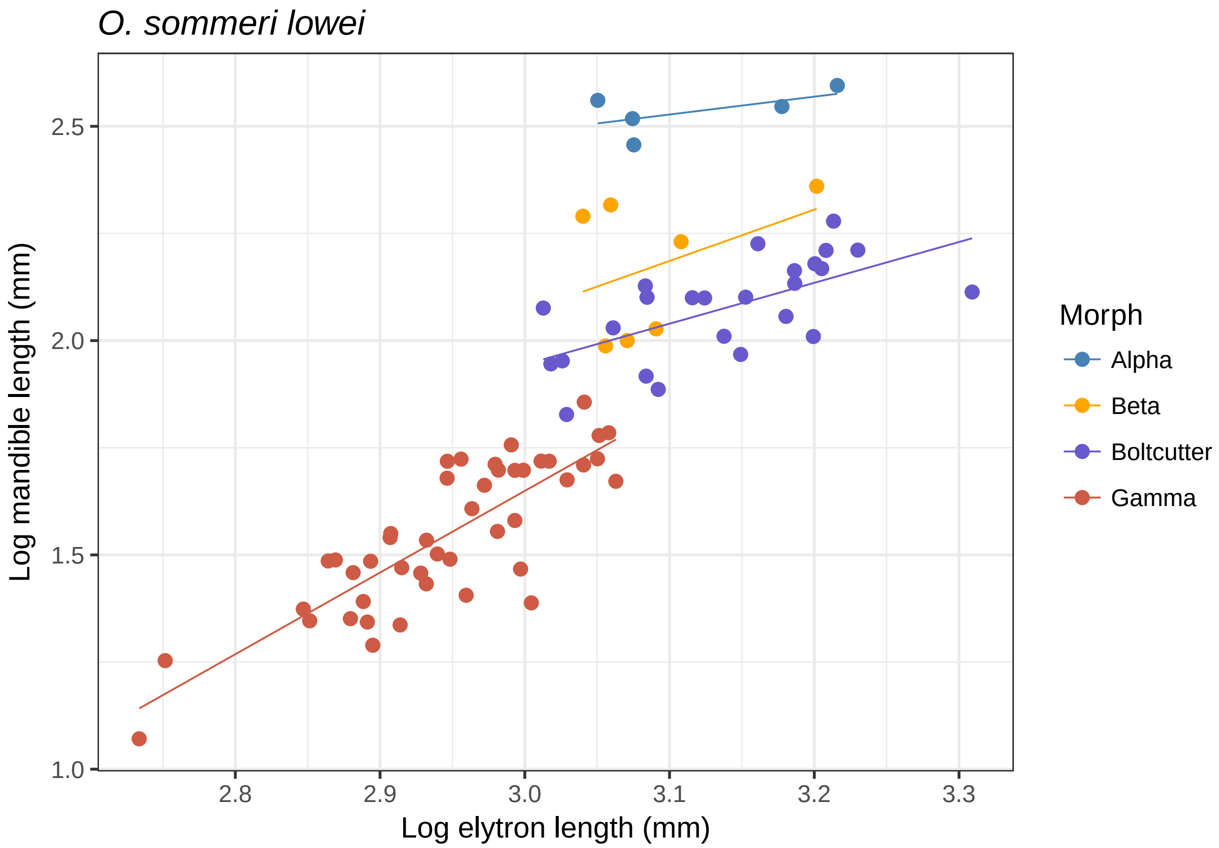

Figure S36 Final allometric plot for *O. sommeri lowei* with morph allocations and lines from the fitted model.

We can also check whether there is a real difference between the boltcutters and the beta morphs by looking at the distance between tooth 1 and tooth 2 on the mandible (as in Rowland and Emlen). This can't be measured in the Alpha males because their mandibles extend to a point and have no teeth at the distal end, but we can measure it for the other morphs.

```
p1 <- ggplot(data = subset(lowei, Morph != "Alpha"), aes(x = left_mandible_straight,
    y = Teeth_1_2, colour = Morph[Morph != "Alpha"]))

p1 <- p1 + geom_point() + theme_bw() + scale_colour_manual(values = palette4)

p1 <- p1 + labs(colour = "Morph") + xlab("Mandible length (mm)") +
    ylab("Distance between teeth 2 & 3 \nat distal end of mandible (mm)")

p1
```

```
## Warning: Removed 3 rows containing missing values (geom_point).
```

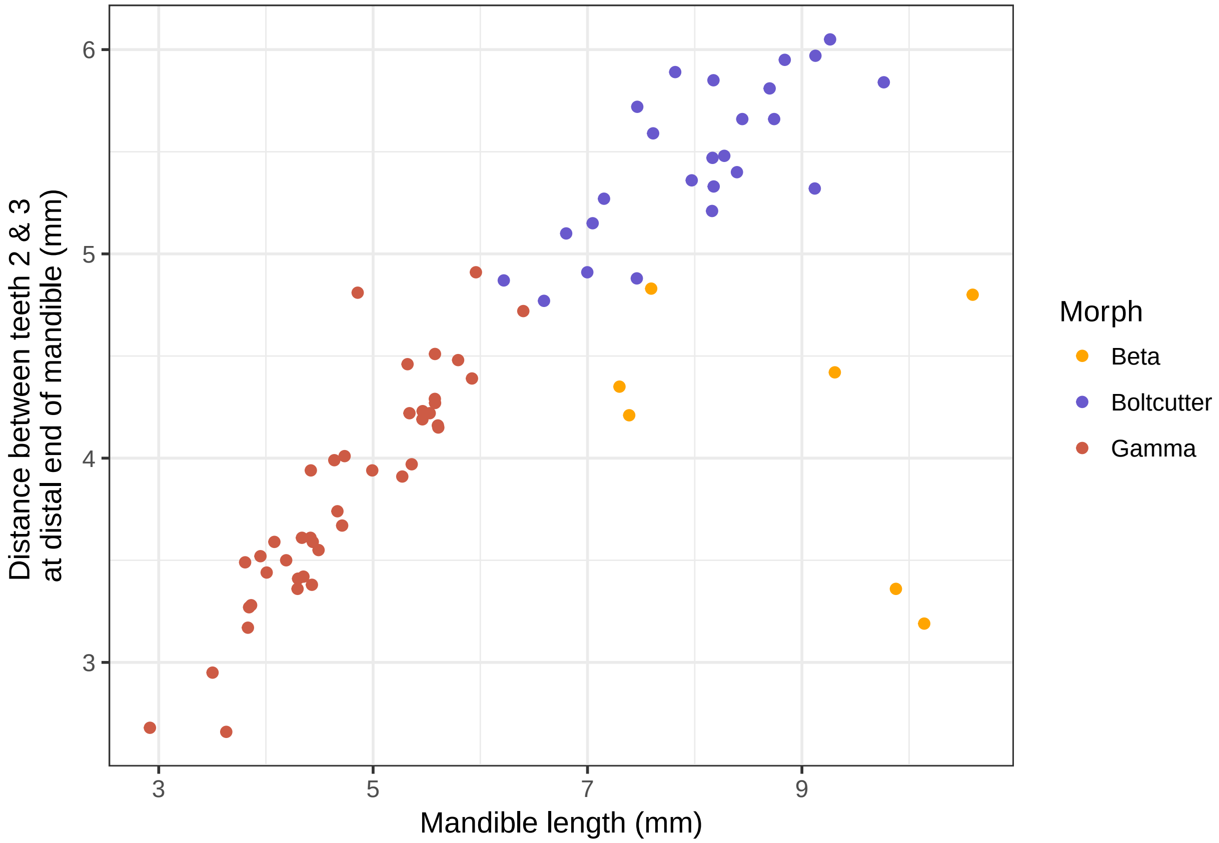

Figure S37 Distance between teeth 2 & 3 on mandibles from all *O. sommeri lowei* morphs except Alphas plotted against mandible length.

There is clear separation between the Boltcutter morphs and the Beta morphs, adding confidence to our interpretation that this species has four male morphs. NB the one Beta (31) which clusters with the Boltcutters is one which was identified as being somewhat intermediate earlier.

Morphometrics for *O. sommeri lowei*

```
lowei <- cbind(lowei, Morph)

s = lowei %>% split(lowei$Morph)

ch = s %>% # Compute which points are on the convex hull of each
# data.frame /!\ Check how 'chull' works
lapply(., function(el) chull(el$pc1, el$pc2))  # 'ch' now contains the
# row numbers of points on convex hull per sub-data.frame

# Get points for each sub-data.frame using names index
ch = lapply(names(ch), function(el) s[[el]][ch[[el]], ]) %>%
    do.call(rbind, .)  # Join all convex hull points in a single data.frame


lowei_pca_plot <- ggplot(data = lowei, aes(x = pc1, y = pc2,
    colour = Morph)) + geom_point(size = 2) + scale_colour_manual(values = palette2) +
    scale_fill_manual(values = palette2) + theme_bw() + xlab("Principal component 1") +
```

```
    ylab("Principal component 2")


lowei_pca_plot <- lowei_pca_plot + geom_polygon(data = ch, aes(fill = Morph,
    colour = NA), alpha = 0.2) + ggtitle("O. sommeri lowei") +
    theme(plot.title = element_text(face = "italic"))

lowei_pca_plot
```

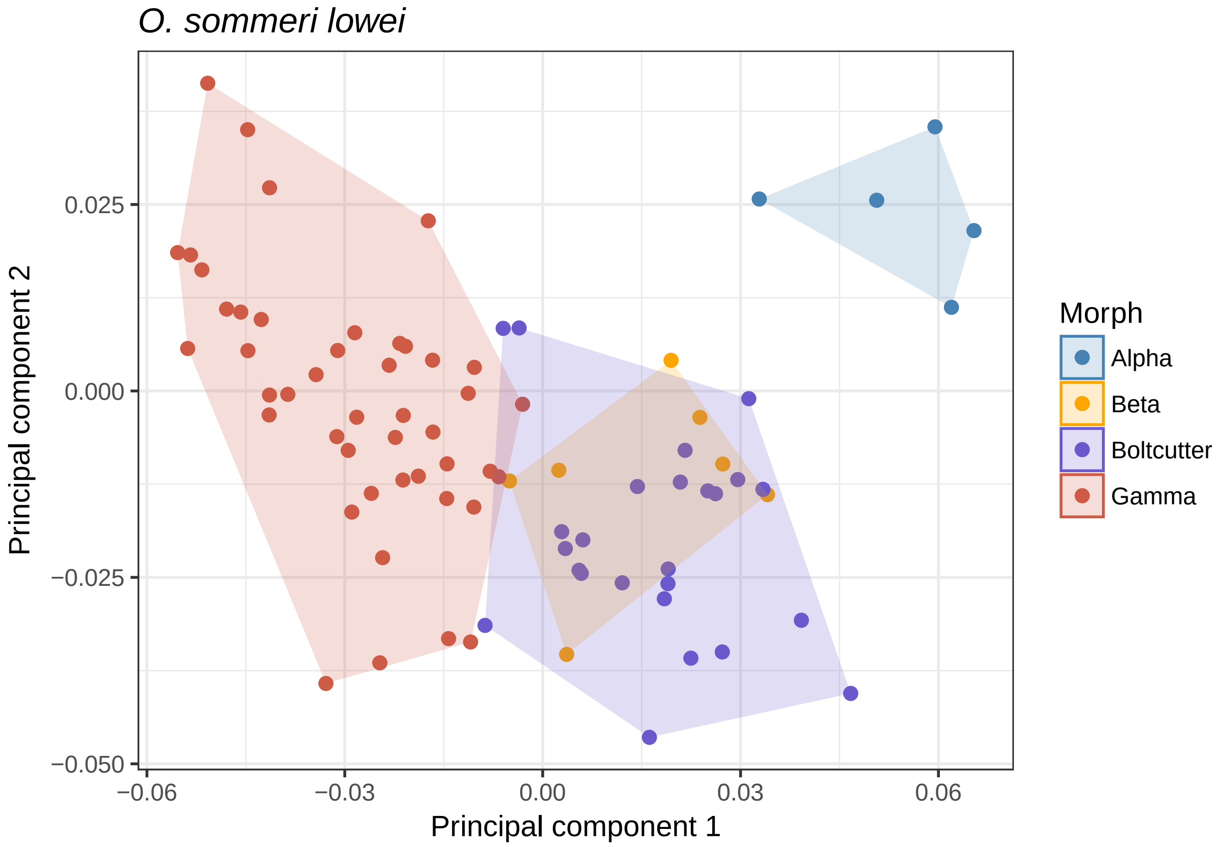

Figure S38 PC plot from relative warp analysis for *O. sommeri lowei*

Alphas cluster distinctly from all other morphs. Betas and boltcutters do not appear to differ but Gammas are separate. Seperation is mainly on PC1 but also on PC2 when comparing Alphas with Betas and Boltcutters.

There is some suggestion of a 'horseshoe effect' in this plot but it is not as pronounced as in some others.

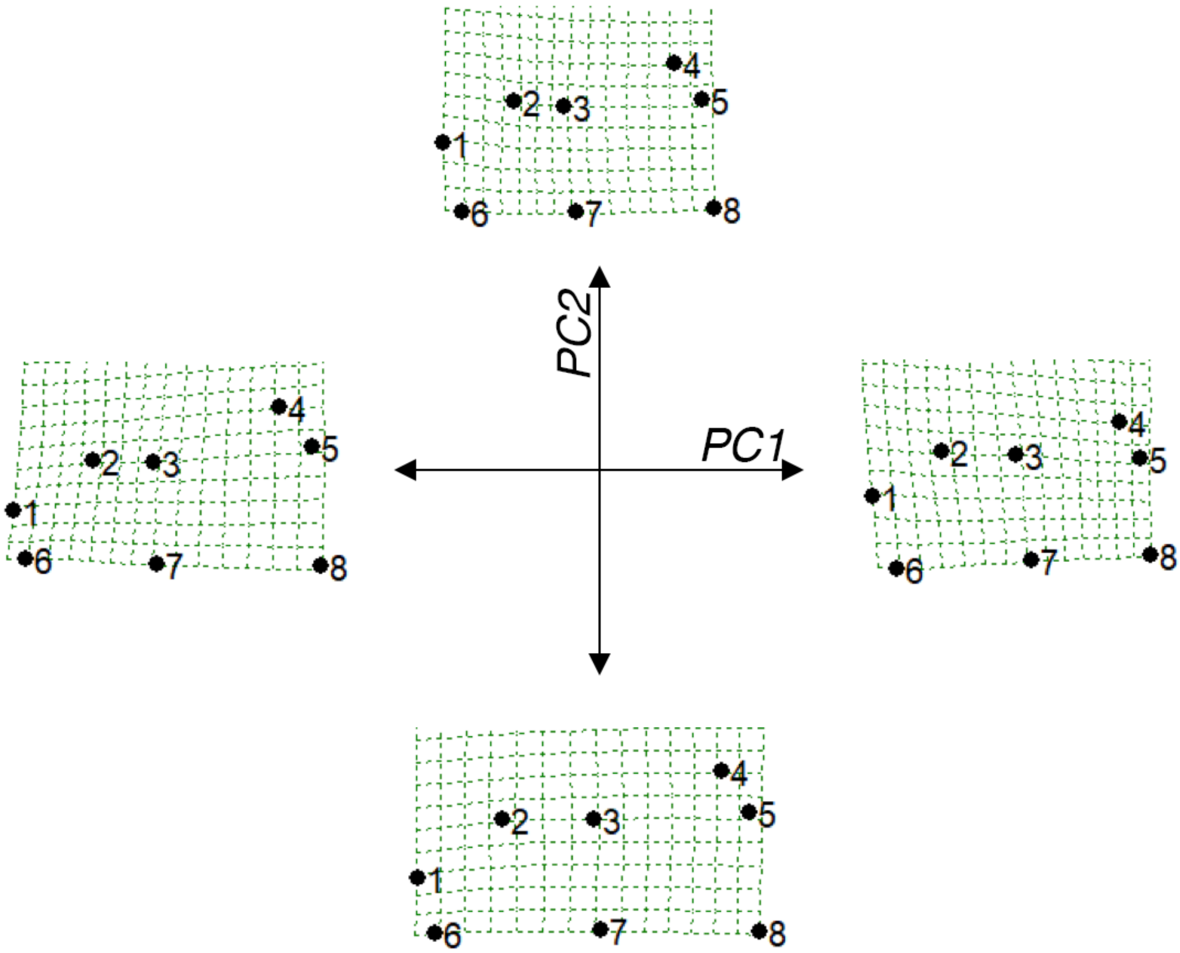

Figure S39 Thin plate splines showing how head and pronotum shape vary along the first two principal components for *O. sommeri lowei*

PC1 and PC2 are similar to those we have already seen, with increasing values of PC1 being associated with a relatively large, wider head and positive values of PC2 being mostly associated with an increasing distance between the eye (landmark 2) and the front of the prothorax.

Betas and boltcutters therefore have larger, wider heads than gammas and alphas have larger, wider heads than betas and boltcutters. Betas and boltcutters also have relatively longer distances between the front of the prothorax and the eye than do alphas.

Is there a strong effect of body size?

```
library(cowplot)

p1 <- ggplot(data = lowei, aes(x = elytra_middle, y = pc1, colour = Morph)) +
    scale_colour_manual(values = palette2) + geom_point(size = 2) +
    theme_bw() + ylab("Principal component 1") + xlab("Elytron length (mm")

p2 <- ggplot(data = lowei, aes(x = elytra_middle, y = pc2, colour = Morph)) +
    scale_colour_manual(values = palette2) + geom_point(size = 2) +
```

```
    theme_bw() + ylab("Principal component 2") + xlab("Elytron length (mm")

plot_grid(p1, p2)
```

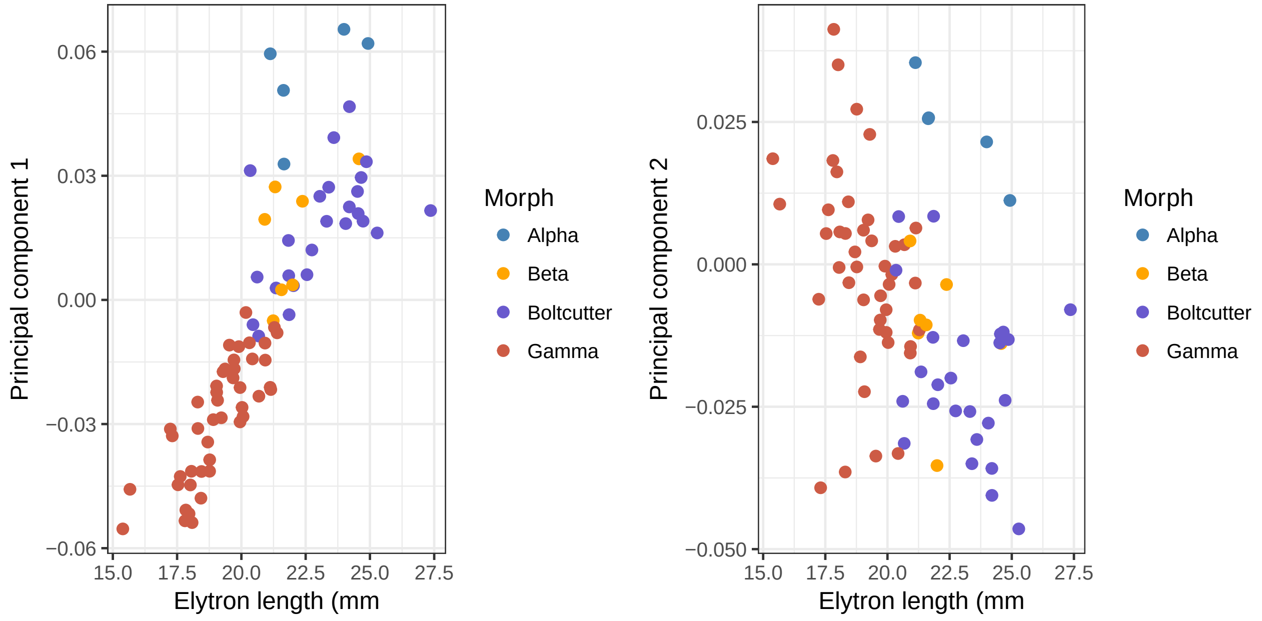

Figure S40 PC1 and PC2 from relative warp analysis plotted against body size (elytron length) for *O. sommeri lowei*

Yes there is. PC1 is strongly related to body size, PC2 less so but still a little.

Analysing the relationship between PC1 and body size using a similar set of candidate models as before:

mod1: four morph different slopes model
mod2: three morph (Gammas and boltcutters combined) different slopes mod3: three morph (Betas and boltcutters combined) different slopes
mod4: four morph same slopes (no interaction)
mod5: three morph (Gammas and boltcutters combined) same slopes
mod6: three morph (Betas and boltcutters combined) same slopes

```
mod1 <- lm(pc1 ~ elytra_middle * Morph, data = lowei)
mod2 <- lm(pc1 ~ elytra_middle * Morph2, data = lowei)
mod3 <- lm(pc1 ~ elytra_middle * Morph3, data = lowei)
mod4 <- lm(pc1 ~ elytra_middle + Morph, data = lowei)
mod5 <- lm(pc1 ~ elytra_middle + Morph2, data = lowei)
mod6 <- lm(pc1 ~ elytra_middle + Morph3, data = lowei)

AIC(mod1, mod2, mod3, mod4, mod5, mod6)
```

```
##      df       AIC
## mod1  9 -507.3016
## mod2  7 -486.9409
## mod3  7 -510.3880
## mod4  6 -507.1775
## mod5  5 -488.1890
## mod6  5 -507.7926
```

The preferred model is model three, which is a three-morph different slopes model with the beta and boltcutter morphs combined. This is consistent with the PCA plot above where Alphas and Gammas clustered separately

but Betas and Boltcutters were very similar.

```r
summary(mod3)
```

```
##
## Call:
## lm(formula = pc1 ~ elytra_middle * Morph3, data = lowei)
##
## Residuals:
##        Min         1Q     Median         3Q        Max
## -0.0170091 -0.0081405 -0.0001289  0.0077489  0.0261553
##
## Coefficients:
##                              Estimate Std. Error t value Pr(>|t|)
## (Intercept)                -0.0450131  0.0693693  -0.649   0.5184
## elytra_middle               0.0043709  0.0030536   1.431   0.1564
## Morph3Gamma                -0.1430578  0.0725953  -1.971   0.0524 .
## Morph3Medium               -0.0430686  0.0736278  -0.585   0.5603
## elytra_middle:Morph3Gamma   0.0039941  0.0032519   1.228   0.2232
## elytra_middle:Morph3Medium  0.0002089  0.0032376   0.065   0.9487
## ---
## Signif. codes:  0 '***' 0.001 '**' 0.01 '*' 0.05 '.' 0.1 ' ' 1
##
## Residual standard error: 0.01027 on 76 degrees of freedom
## Multiple R-squared:  0.8903, Adjusted R-squared:  0.8831
## F-statistic: 123.4 on 5 and 76 DF,  p-value: < 2.2e-16
```

```r
pred <- predict(mod3)

p1 <- ggplot(data = cbind(lowei, pred), aes(x = elytra_middle,
    y = pc1, colour = Morph)) + geom_point(size = 2) + scale_colour_manual(values = palette2) +
    theme_bw() + xlab("Elytron length (mm)") + ylab("Principal component 1")

p1 <- p1 + geom_line(aes(y = pred))

p1
```

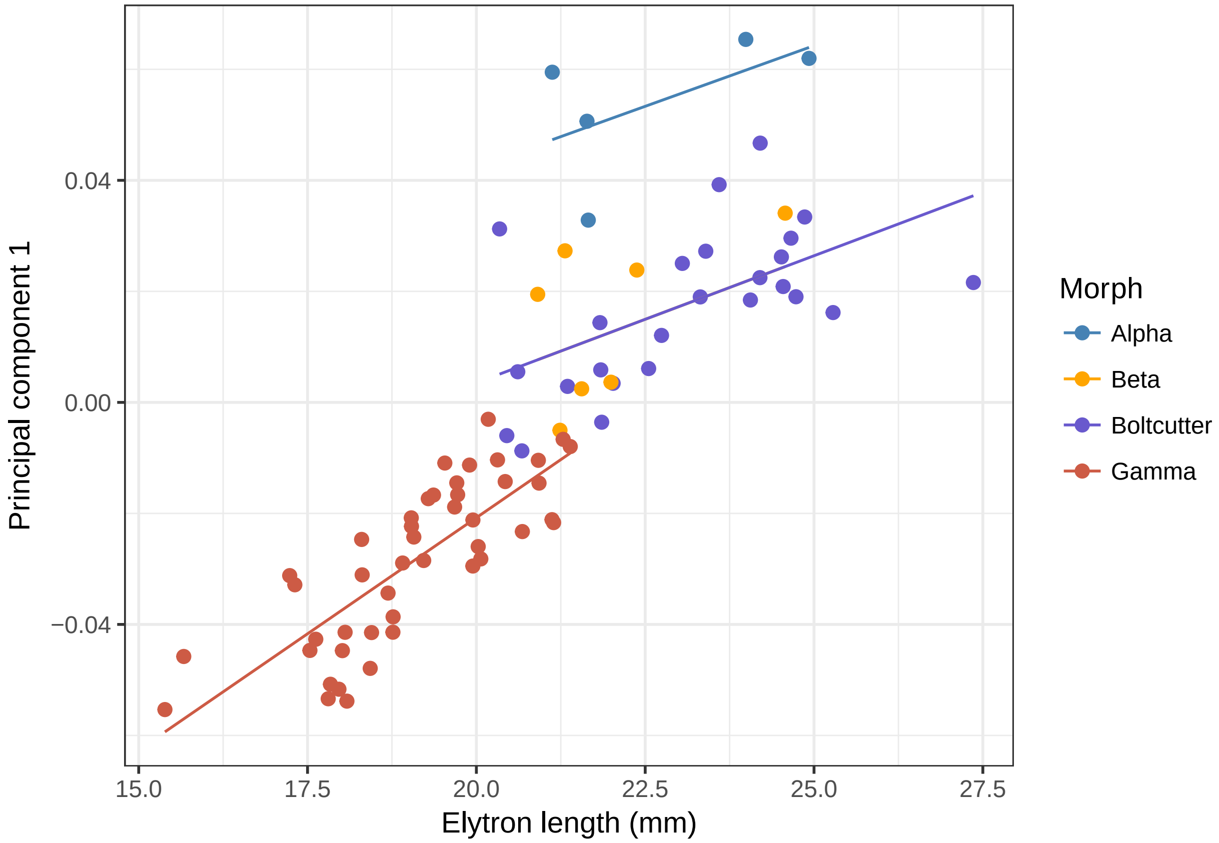

Figure S41 PC1 from relative warp analysis plotted against elytron length for *O. sommeri lowei* with lines showing predicted values from model.

```
Lowei_Morph <- Morph

rm(lowei)
```

O. sommeri s.stricto

Visual inspection finds some obvious beta and alpha males, including some very small males that are clearly betas. The larger males with 'short' mandibles resemble 'Boltcutters' but there is no obvious differentiation with smaller males who resemble Gammas more. Examination of a histogram of mandible length to body size ratios does not indicate an obvious bimodalism, and neither does a scatterplot of mandible length versus body size. We therefore conclude that this species has three morphs: Alphas, Betas and Gammas. The species differs from *O. cuvera* in the lack of any obvious threshold: the largest individuals in the sample are in fact Gamma morphs.

Note that with a larger sample size it might be the case that the large and small Gammas could be differentiated into two groups.

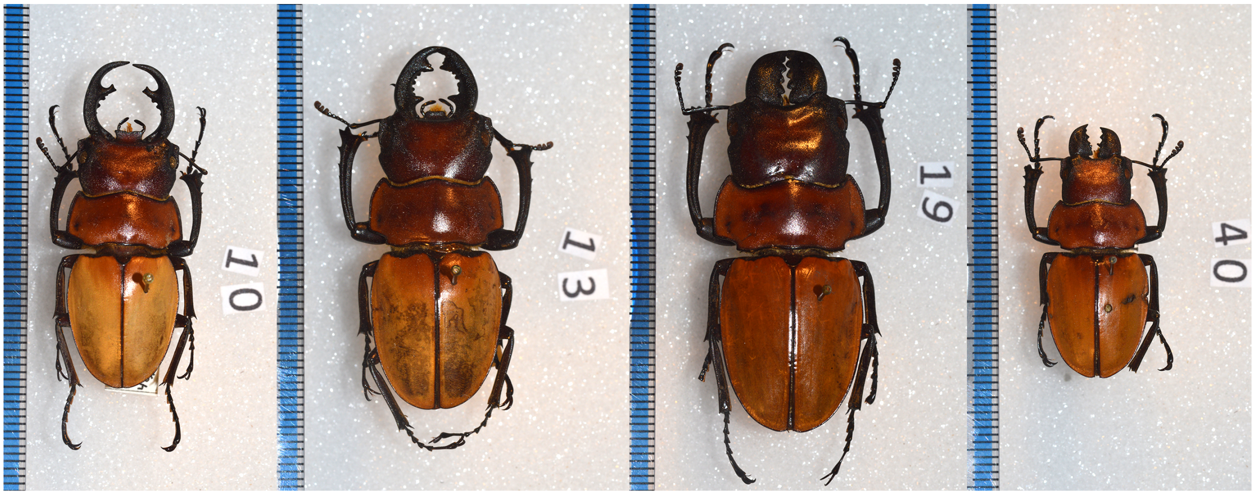

Alpha Beta Large Gamma Small Gamma

Odontolabis sommeri s. stricto

Figure S42 Examples of the different morphs for *O. sommeri s.s.*

```
sommeri <- subset(alldata, species == "sommeri")

sommeri2 <- subset(sommeri, morph_visual == "Gamma")

ggplot(data = sommeri, aes(left_mandible_straight/elytra_middle)) +
    geom_histogram(aes(y = ..density..), fill = "grey50", colour = "black",
        bins = 20) + theme_bw() + geom_density(bw = 0.02) + ggtitle("Histogram of mandible to elytron ra
```

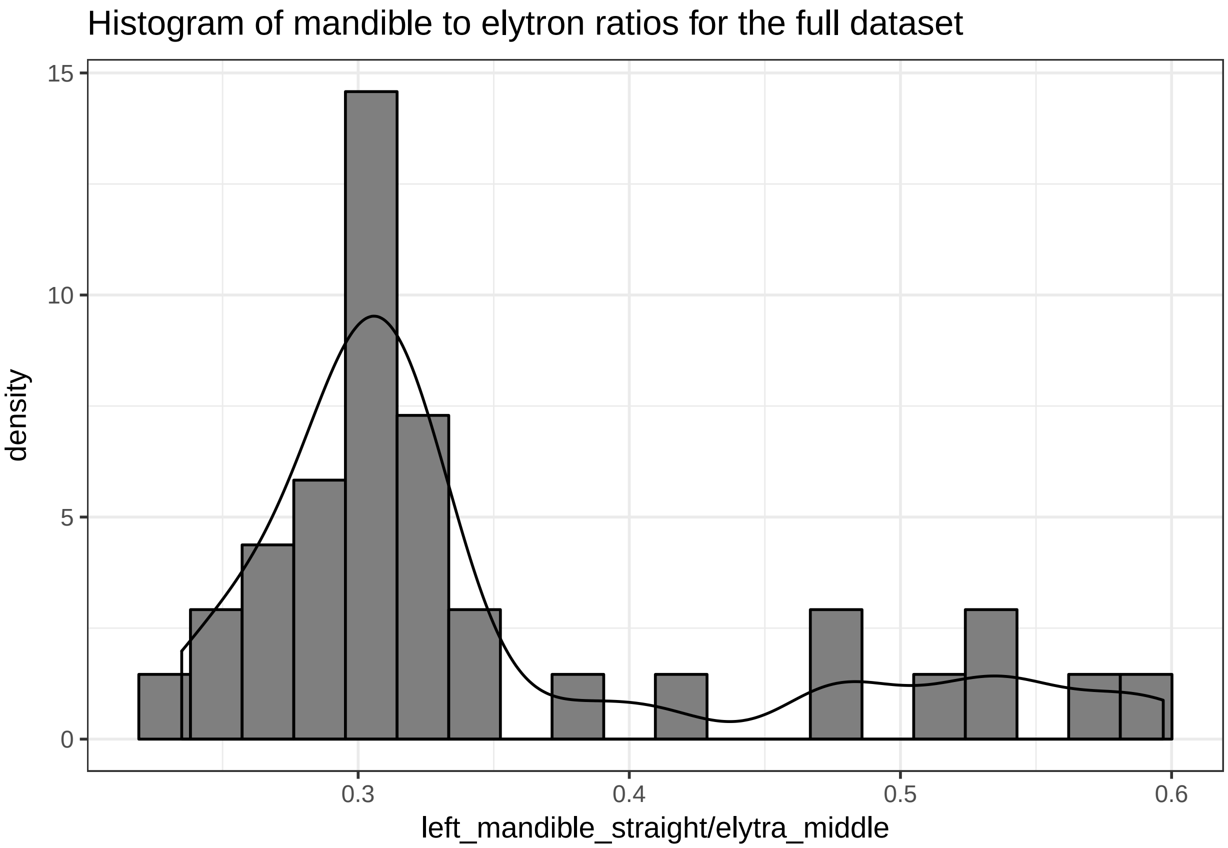


```r
# Set up new variable for ratio
ratio <- sommeri$left_mandible_straight/sommeri$elytra_middle




# Gaussian models specified for mixture model
g1 <- FLXMRglm(family = "gaussian")
g2 <- FLXMRglm(family = "gaussian")
g3 <- FLXMRglm(family = "gaussian")
# g4 <- FLXMRglm(family = 'gaussian')

# Fit three mixture models mix.mod1<-flexmix(ratio~1, k = 2,
# model = list(g1, g2))

mix.mod2 <- flexmix(ratio ~ 1, k = 3, model = list(g1, g2, g3),
    cluster = as.numeric(droplevels(sommeri$morph_visual)))
# mix.mod2<-flexmix(ratio~1, k = 3, cluster =
# as.numeric(droplevels(sommeri$morph_visual)))

# mix.mod3<-flexmix(ratio~1, k = 4, model = list(g1, g2, g3,
# g4), cluster = as.numeric(lowei$morph_visual))

# AIC(mix.mod1, mix.mod2)
```

```
# mix.mod2 (3 clusters) has much lower AIC



#### plot histogram

x1 <- seq(min(ratio), max(ratio), length = 100)

counts <- table(clusters(mix.mod2))

d1 <- dnorm(x1, parameters(mix.mod2)[[1]][1, 1], parameters(mix.mod2)[[1]][2,
    1]) * counts[1]/sum(counts)
d2 <- dnorm(x1, parameters(mix.mod2)[[1]][1, 2], parameters(mix.mod2)[[1]][2,
    2]) * counts[2]/sum(counts)
d3 <- dnorm(x1, parameters(mix.mod2)[[1]][1, 3], parameters(mix.mod2)[[1]][2,
    3]) * counts[3]/sum(counts)


densities <- data.frame(x1, d1, d2, d3)

p1 <- ggplot(data = data.frame(ratio), aes(ratio, ..density..)) +
    geom_histogram(fill = "grey80", colour = "black", bins = 16) +
    theme_bw() + xlab("Mandible length / elytron length")

p1 <- p1 + geom_line(data = densities, aes(x = x1, y = d1))
p1 <- p1 + geom_line(data = densities, aes(x = x1, y = d2))
p1 <- p1 + geom_line(data = densities, aes(x = x1, y = d3))

p1
```

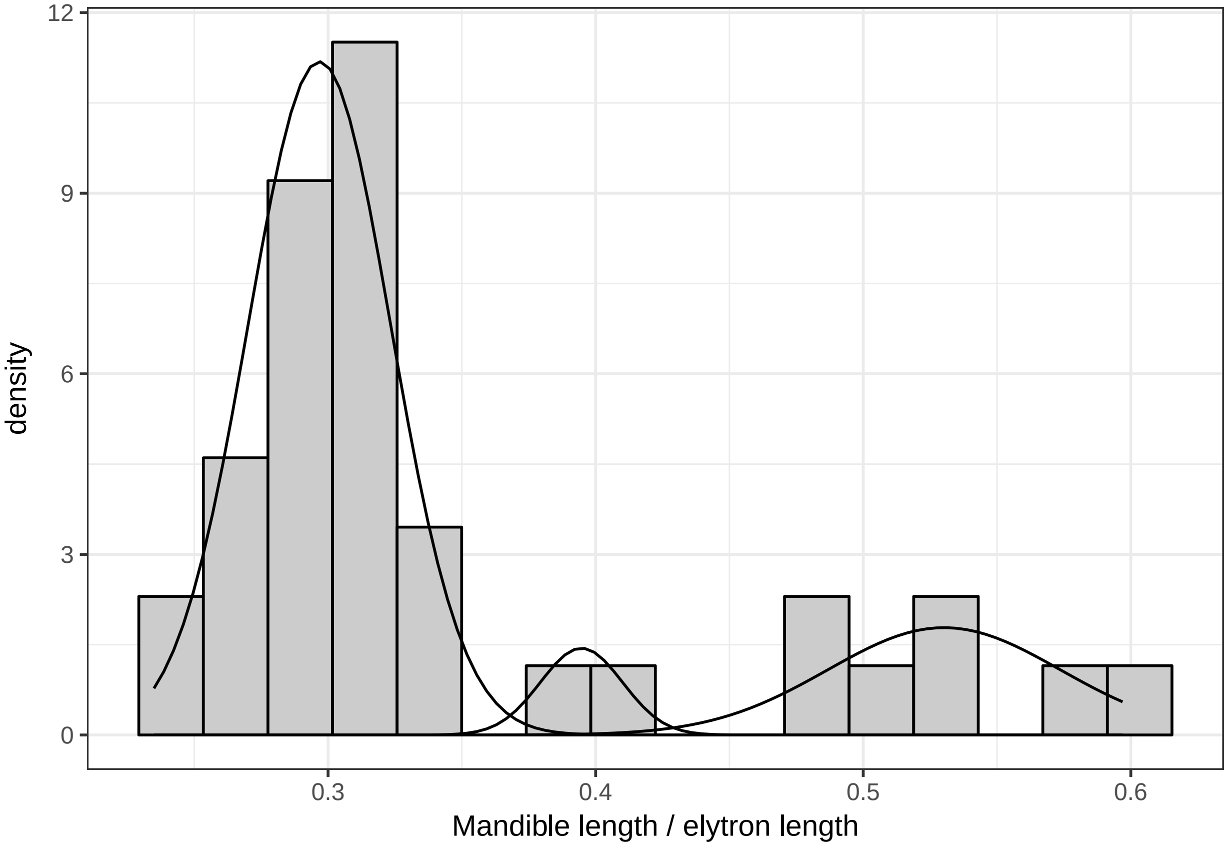

Figure S43 Histogram showing the frequency distribution of ratios of mandible length to elytra length for *O. sommeri s.s.* plus fitted distributions from the mixture model.

```
### Morph allocation

# Morph <- sommeri$morph_visual

Morph <- ifelse(clusters(mix.mod2) == 1, "Alpha", "Beta")
Morph <- ifelse(clusters(mix.mod2) == 3, "Gamma", Morph)


p1 <- ggplot(data = sommeri, aes(x = elytra_middle, y = left_mandible_straight,
    colour = morph_visual, shape = Morph))
p1 <- p1 + geom_point(size = 2) + scale_colour_manual(values = palette1) +
    theme_bw() + xlab("Elytron length (mm)") + ylab("Mandible length")

p1
```

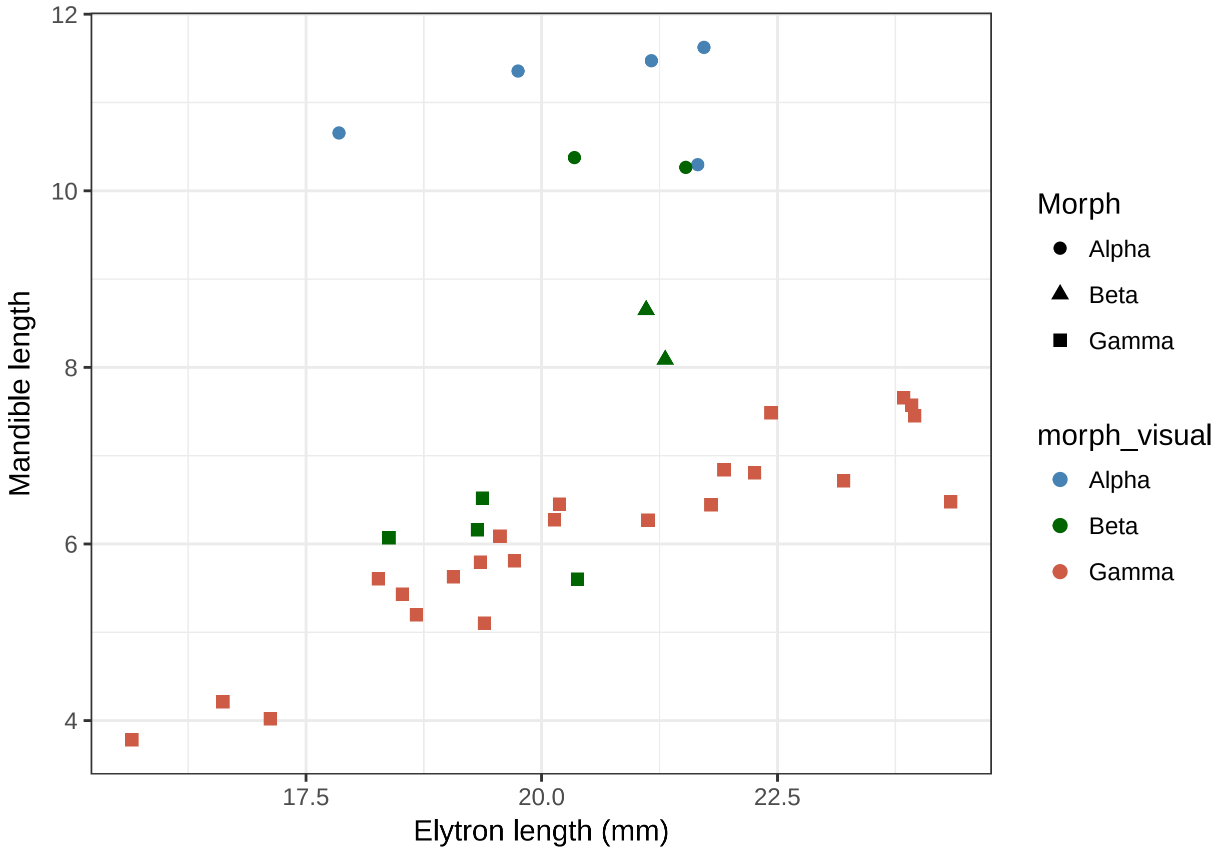

Figure S45 Mandible length against Elytron length with the initial morph allocation shown for *O sommeri s.s.*. Morph is the allocation from the mixture model, morph_visual is the allocation from the visual inspection.

The mixture model really doesn't capture much of the variation here and in particular fails to categorise most of the beta morphs. Following review of the data we use the visual allocations only.

There is a suggestion of a break in the Gammas at about 20.5 mm elytron length but this is more likely to be a sampling artefact- it looks as though there is a break because there are no specimens in that region.

Model fitting for *O. sommeri s.s.*

Comparison of candidate models explaining the variation in mandible length.

Mod1: three morph, different slopes model Mod2: three morph, same slopes (no interaction)

```
Morph <- sommeri$morph_visual
sommeri <- data.frame(sommeri, Morph)
rm(Morph)

mod1 <- lm(log(left_mandible_straight) ~ log(elytra_middle) *
    Morph, data = sommeri)
mod2 <- lm(log(left_mandible_straight) ~ log(elytra_middle) +
    Morph, data = sommeri)

AIC(mod1, mod2)
```

```
##      df       AIC
```

```
## mod1  7 -50.65649
## mod2  5 -45.76454
```

The three morph, different slopes model has the lowest AIC and is preferred.

```
sommeri_mod <- mod1

summary(sommeri_mod)
```

```
##
## Call:
## lm(formula = log(left_mandible_straight) ~ log(elytra_middle) *
##     Morph, data = sommeri)
##
## Residuals:
##      Min       1Q   Median       3Q      Max
## -0.32133 -0.05269  0.00558  0.04617  0.29957
##
## Coefficients:
##                              Estimate Std. Error t value Pr(>|t|)
## (Intercept)                    1.9340     1.9554   0.989  0.33054
## log(elytra_middle)             0.1560     0.6485   0.240  0.81159
## MorphBeta                     -8.9137     2.9491  -3.023  0.00509 **
## MorphGamma                    -4.5765     2.0345  -2.249  0.03198 *
## log(elytra_middle):MorphBeta   2.8377     0.9798   2.896  0.00699 **
## log(elytra_middle):MorphGamma  1.3131     0.6748   1.946  0.06108 .
## ---
## Signif. codes:  0 '***' 0.001 '**' 0.01 '*' 0.05 '.' 0.1 ' ' 1
##
## Residual standard error: 0.108 on 30 degrees of freedom
## Multiple R-squared:  0.8814, Adjusted R-squared:  0.8617
## F-statistic:  44.6 on 5 and 30 DF,  p-value: 5.319e-13
```

The slope for Alphas is essentially zero, but Gammas and especialy Betas have much higher allometric slopes.

```
# pred <- predict(sommeri_mod)
pred <- sommeri_mod$fitted.values

sommeri2 <- cbind(sommeri, pred)

sommeri_plot1 <- ggplot(data = sommeri, aes(x = log(elytra_middle),
    y = log(left_mandible_straight), colour = Morph)) + geom_point(size = 2) +
    theme_bw() + xlab("Log elytron length (mm)") + scale_colour_manual(values = palette1) +
    ylab("Log mandible length (mm)")

sommeri_plot1 <- sommeri_plot1 + geom_line(aes(y = pred), size = 0.33)

# sommeri_plot1 <- sommeri_plot1 + geom_smooth(method = 'lm',
# se = FALSE, size = 0.33)

sommeri_plot1 <- sommeri_plot1 + ggtitle("O. sommeri s.s.") +
    theme(plot.title = element_text(face = "italic"))

sommeri_plot1
```

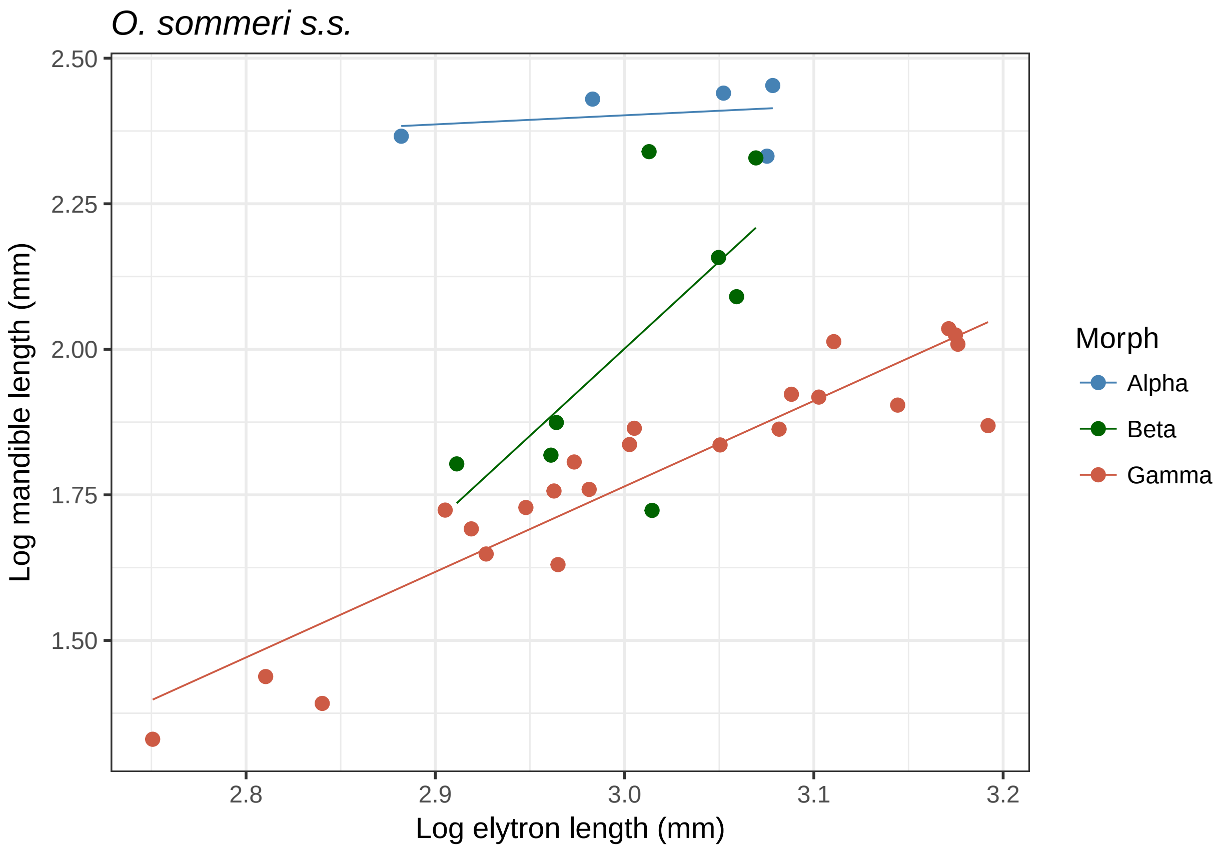

Figure S46 Final allometric plot for *O. sommeri s.s.* with morph allocations and lines from the fitted model.

Morphometrics for *O. sommeri s.stricto*

```
s = sommeri %>% split(sommeri$Morph)

ch = s %>% # Compute which points are on the convex hull of each
# data.frame /!\ Check how 'chull' works
lapply(., function(el) chull(el$pc1, el$pc2))  # 'ch' now contains the
# row numbers of points on convex hull per sub-data.frame

# Get points for each sub-data.frame using names index
ch = lapply(names(ch), function(el) s[[el]][ch[[el]], ]) %>%
    do.call(rbind, .)  # Join all convex hull points in a single data.frame


sommeri_pca_plot <- ggplot(data = sommeri, aes(x = pc1, y = pc2,
    colour = Morph)) + geom_point(size = 2) + scale_colour_manual(values = palette1) +
    scale_fill_manual(values = palette1) + theme_bw() + xlab("Principal component 1") +
    ylab("Principal component 2")


sommeri_pca_plot <- sommeri_pca_plot + geom_polygon(data = ch,
    aes(fill = Morph, colour = NA), alpha = 0.2) + ggtitle("O. sommeri s.s.") +
    theme(plot.title = element_text(face = "italic"))
```

```
sommeri_pca_plot
```

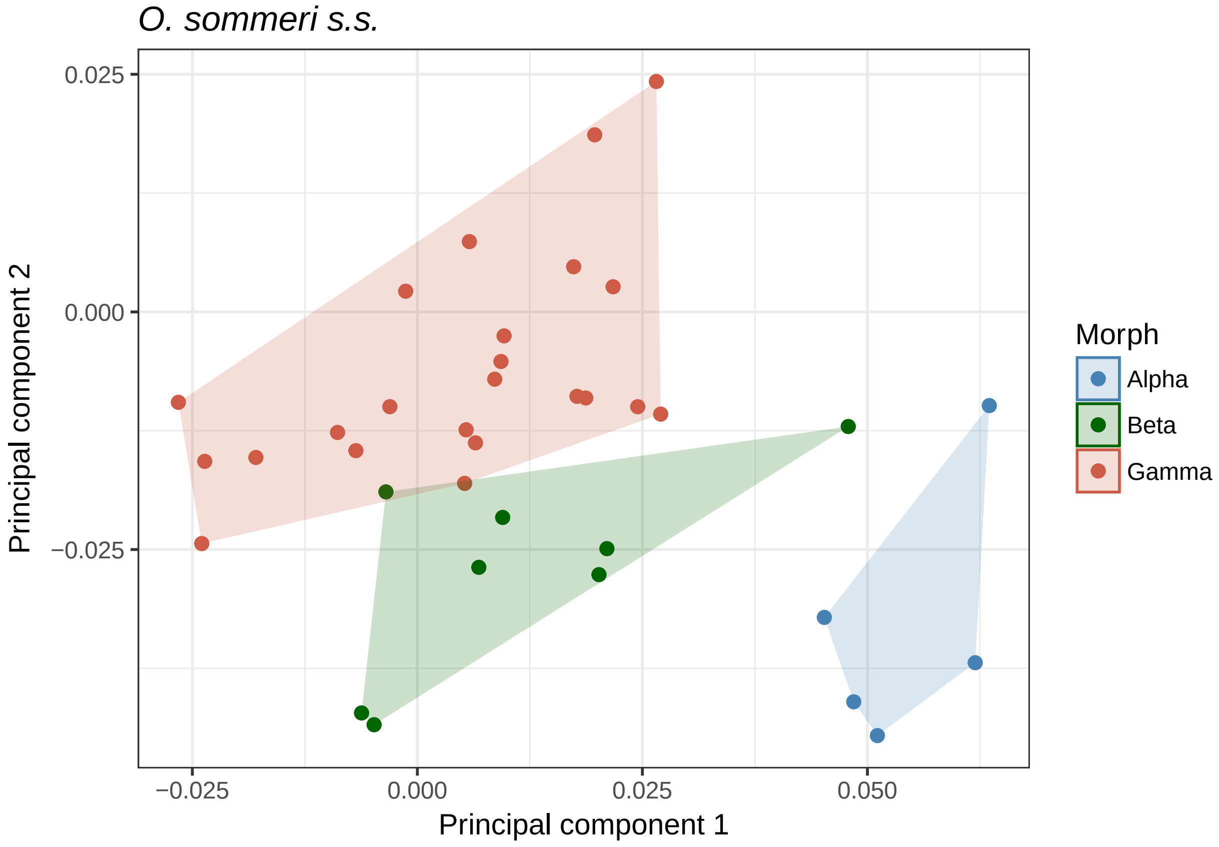

Figure S47 PC plot from relative warp analysis for *O. sommeri s.s.*

There is clear separation between the morphs, with Alphas especially separating from Betas and Gammas on the basis of PC1. NB the one Beta which is close to the Alpha group is the Beta with the longest mandibles, which is in fact intermediate between the two - it was classified as a Beta becuase it resembled a Beta more than an Alpha but it lacks the two teeth towards the base of the mandibles which are an important diagnostic feature of the Beta morph.

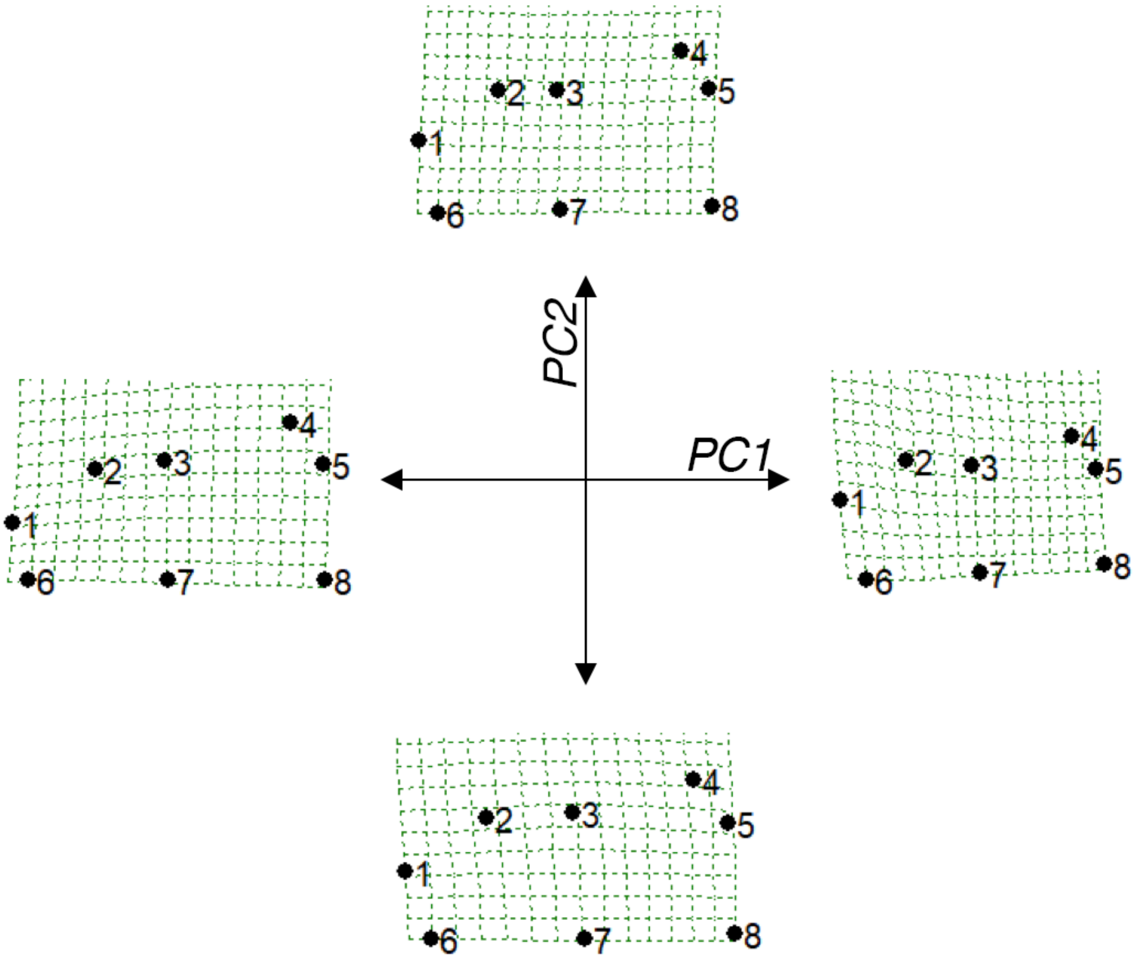

Figure S48 Thin plate splines showing how head and pronotum shape vary along the first two principal components for *O. sommeri s.s.*

Body size effects

```
p1 <- ggplot(data = sommeri, aes(x = elytra_middle, y = pc1,
    colour = Morph)) + geom_point(size = 2) + scale_colour_manual(values = palette1) +
    scale_fill_manual(values = palette1) + theme_bw() + ylab("Principal component 1") +
    xlab("Elytra length (mm)")

p1
```

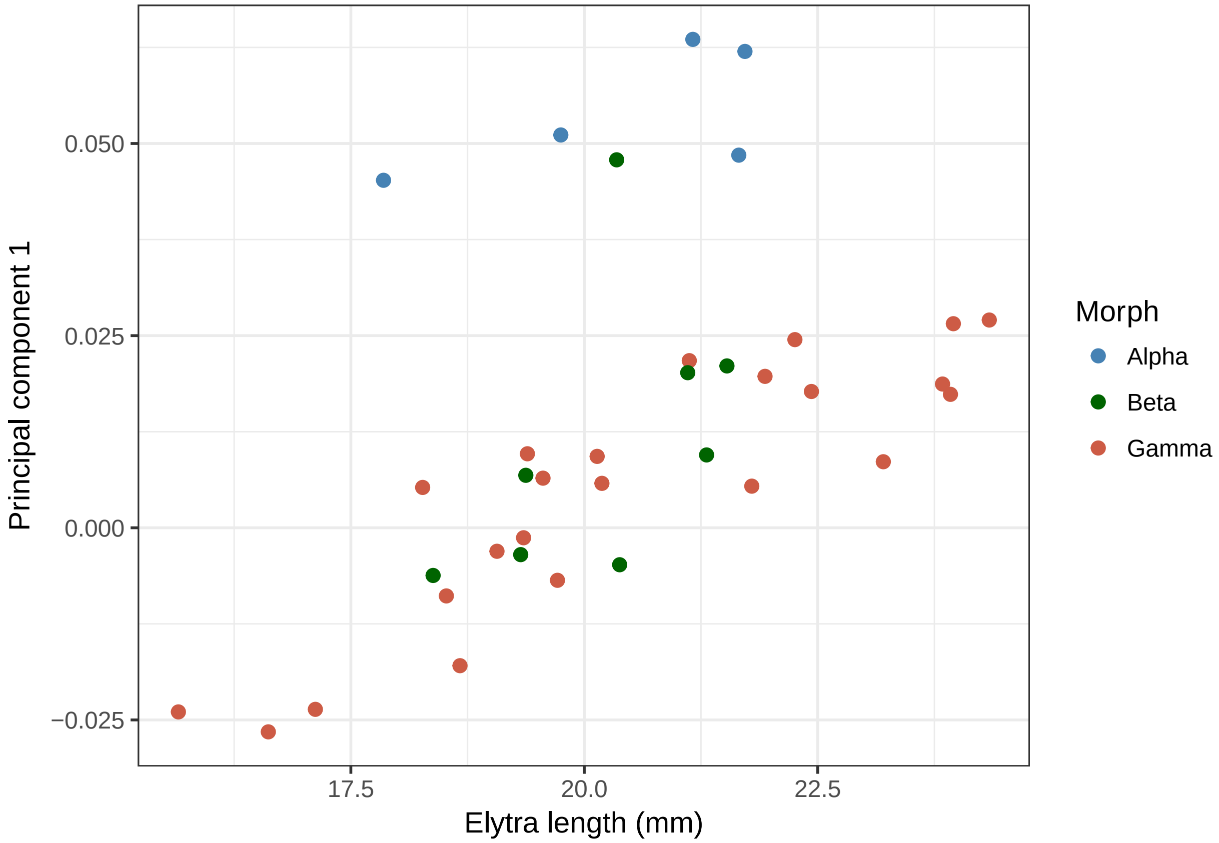

Figure S49 PC1 from relative warp analysis plotted against body size (elytron length) for *O. sommeri s.s.*

There is an obvious body size effect with PC1 declining with bigger bodies, but the Alphas (and the intermediate male) are clearly separated from the remainder of the males.

Modeling:

Mod1: Three morphs different slopes Mod2: Two morphs (Gamma and Betas combined), different slopes Mod3: Three morphs same slopes (no interaction) Mod4: Two morphs same slopes Mod5: Elytron length only as an explanatory variable

```
Morph2 <- ifelse(sommeri$Morph == "Alpha", "Alpha", "Other")

mod1 <- lm(pc1 ~ elytra_middle * Morph, data = sommeri)
mod2 <- lm(pc1 ~ elytra_middle * Morph2, data = sommeri)
mod3 <- lm(pc1 ~ elytra_middle + Morph, data = sommeri)
mod4 <- lm(pc1 ~ elytra_middle + Morph2, data = sommeri)
mod5 <- lm(pc1 ~ elytra_middle, data = sommeri)

# mod1 <- lm(pc1 ~ elytra_middle * Morph, data = sommeri,
# subset = -6) mod2 <- lm(pc1 ~ elytra_middle * Morph2, data
# = sommeri, subset = -6) mod3 <- lm(pc1 ~ elytra_middle +
# Morph, data = sommeri, subset = -6) mod4 <- lm(pc1 ~
# elytra_middle + Morph2, data = sommeri, subset = -6) mod5
# <- lm(pc1 ~ elytra_middle, data = sommeri, subset = -6)

AIC(mod1, mod2, mod3, mod4, mod5)
```

```
##      df       AIC
```

```
## mod1  7 -218.9589
## mod2  5 -218.5188
## mod3  5 -221.6944
## mod4  4 -219.8416
## mod5  3 -175.9497
```

All models with Alpha morphs separated from the others give a lower AIC than the model without but the AIC scores for mod2, mod3 and mod4 are sufficiently similar that we don't really have a good indication of which is the best. This means that we have good condfidence that we need a model that distinguishes Alphas from others but we don't have much confidence as to whether we should also separate Betas from Boltcutters to explain the variability in pc1. Removing the intermediate Beta/Alpha male gives a slightly clearer result result with mods 1 & 2 being excluded but mods 3 & 4 are still separated by less than 2 on AIC score.

```
Sommeri_Morph <- sommeri$Morph
Sommeri_Morph <- droplevels(Sommeri_Morph)

rm(sommeri)
```

Morph proportions by species

```
Numbers <- matrix(data = rep(0, times = 24), nrow = 6, ncol = 4)
rownames(Numbers) <- c("Cuvera", "Brookeana", "Lowei", "Sommeri",
    "Platy", "Siva")
colnames(Numbers) <- c("Alpha", "Beta", "Boltcutter", "Gamma")

Numbers[2, ] <- summary(as.factor(Brook_Morph))
Numbers[1, c(1:2, 4)] <- summary(as.factor(Cuvera_Morph))
Numbers[5, c(1, 4)] <- summary(as.factor(Platy_Morph))
Numbers[6, c(1, 4)] <- summary(as.factor(Siva_Morph))
Numbers[3, ] <- summary(as.factor(Lowei_Morph))
Numbers[4, c(1:2, 4)] <- summary(as.factor(Sommeri_Morph))

Totals <- rowSums(Numbers)

Species <- factor(rownames(Numbers), levels = c("Cuvera", "Brookeana",
    "Lowei", "Sommeri", "Platy", "Siva"))


Numbers1 <- data.frame(Species, Numbers)

library(reshape2)

Proportions_long <- melt(Numbers1, variable.name = "Morph")
```

```
## Using Species as id variables
```

```
names(Proportions_long) <- c("Species", "Morph", "Count")

Totals2 <- data.frame(Species, Totals)

Proportions <- merge.data.frame(Proportions_long, Totals2, by = "Species")

Proportions$Proportion <- round(Proportions$Count/Proportions$Totals,
```

```
    3)


library(binom)


CIs <- binom.confint(Proportions$Count, Proportions$Totals, methods = "exact")

Proportions <- data.frame(Proportions, CIs$lower, CIs$upper)

species_labels1 <- c(Cuvera = "O. cuvera", Brookeana = "O. brookeana",
    Lowei = "O. sommeri lowei", Sommeri = "O. sommeri s.s.")
species_labels2 <- c(Platy = "O. platynota", Siva = "O. siva")


p1 <- ggplot(data = subset(Proportions, Species != "Platy" &
    Species != "Siva"), aes(x = Morph, y = Proportion, fill = Morph))

p1 <- p1 + geom_col(position = position_dodge()) + theme_bw() +
    scale_fill_manual(values = palette2) + ylab("Proportion") +
    xlab("") + scale_x_discrete(breaks = NULL)

p1 <- p1 + geom_errorbar(aes(ymin = CIs.lower, ymax = CIs.upper),
    width = 0, position = position_dodge(0.9))

p1 <- p1 + facet_grid(. ~ Species, switch = "both", labeller = labeller(Species = species_labels1)) +
    theme(strip.text = element_text(face = "italic"), strip.background = element_rect(colour = "white",
        fill = "white"))

Prop_2morph <- subset(Proportions, Species == "Platy" | Species ==
    "Siva")

Prop_2morph <- subset(Prop_2morph, Morph == "Alpha" | Morph ==
    "Gamma")



p2 <- ggplot(data = Prop_2morph, aes(x = Morph, y = Proportion,
    fill = Morph))

p2 <- p2 + geom_col(position = position_dodge()) + theme_bw() +
    scale_fill_manual(values = palette3) + ylab("Proportion") +
    xlab("") + scale_x_discrete(breaks = NULL)

p2 <- p2 + geom_errorbar(aes(ymin = CIs.lower, ymax = CIs.upper),
    width = 0, position = position_dodge(0.9))

p2 <- p2 + facet_grid(. ~ Species, switch = "both", labeller = labeller(Species = species_labels2)) +
    theme(strip.text = element_text(face = "italic"), strip.background = element_rect(colour = "white",
        fill = "white"))

p1
```

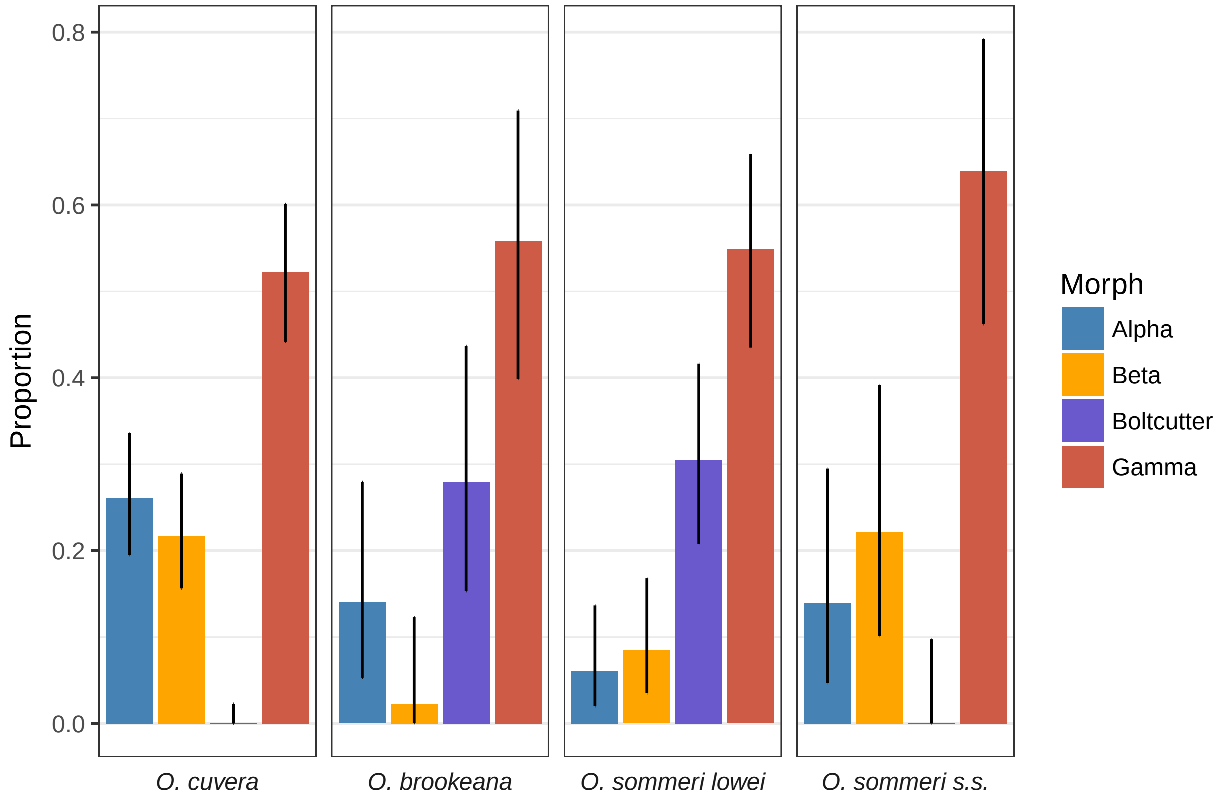


```
p2
```

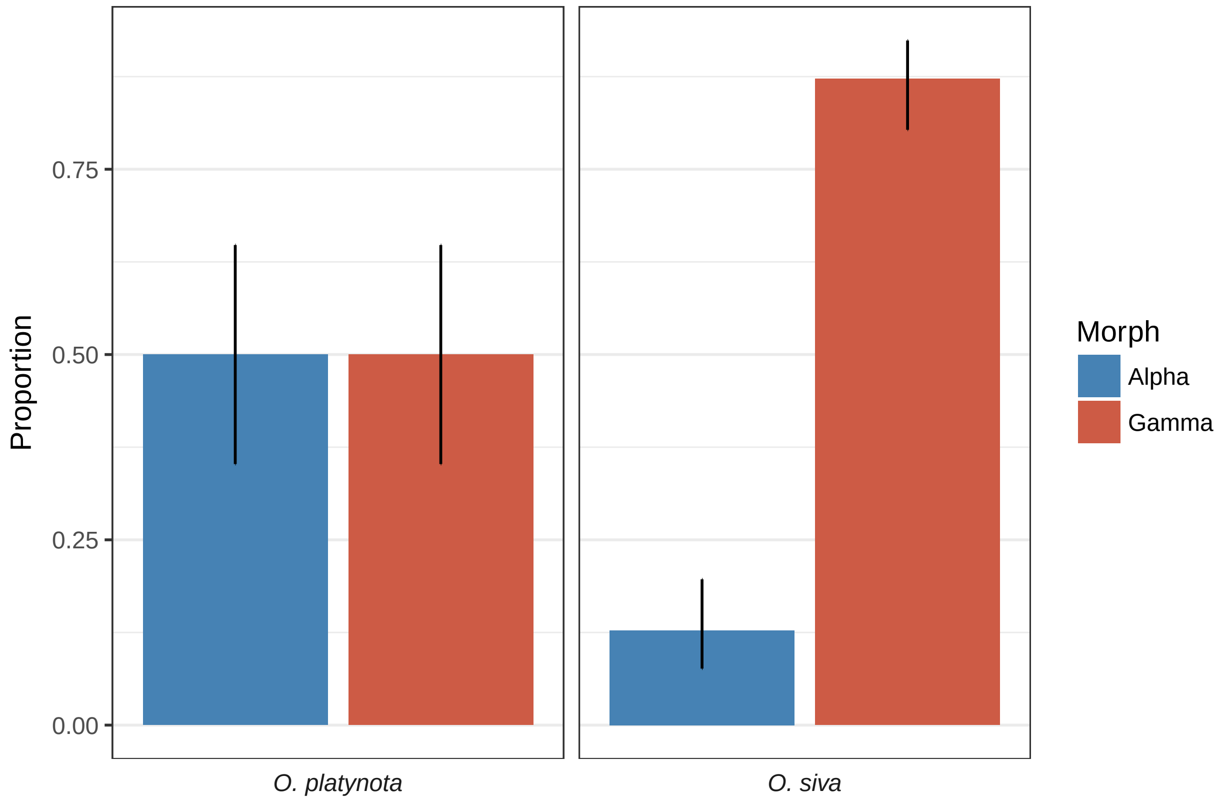

Figure S50 Proportions of each morph per species. Error bars show 95% confidence intervals.

Figure with all species allometry

```r
alldata <- rbind(brook2, cuvera2, platy2, siva2, lowei2, sommeri2)

alldata$species = factor(alldata$species, levels(alldata$species)[c(1:3,
    6, 4, 5)])  #Reorder levels

species_names <- c(brookeana = "O. brookeana", cuvera = "O. cuvera",
    lowei = "O. sommeri lowei", sommeri = "O. sommeri s.s.",
    platynota = "O. platynota", siva = "O. siva")


###### Allometry plot

allometry_plot1 <- ggplot(data = alldata, aes(x = log(elytra_middle),
    y = log(left_mandible_straight), colour = Morph)) + geom_point(size = 2) +
    scale_colour_manual(values = palette2) + theme_bw() + theme(panel.grid.major = element_blank(),
    panel.grid.minor = element_blank()) + xlab("Log elytron length (mm)") +
    ylab("Log mandible length (mm)")

allometry_plot1 <- allometry_plot1 + geom_line(aes(y = pred),
    size = 0.33)

allometry_plot1 <- allometry_plot1 + facet_wrap(~species, nrow = 3,
    ncol = 2, scales = "free", labeller = labeller(species = species_names))

allometry_plot1 <- allometry_plot1 + theme(strip.background = element_rect(fill = "white",
    colour = "white"), strip.text = element_text(face = "bold.italic",
    size = 12), axis.title = element_text(face = "bold", size = 11),
    legend.title = element_text(face = "bold", size = 11))

allometry_plot1
```

```
## Warning: Removed 1 rows containing missing values (geom_path).
```

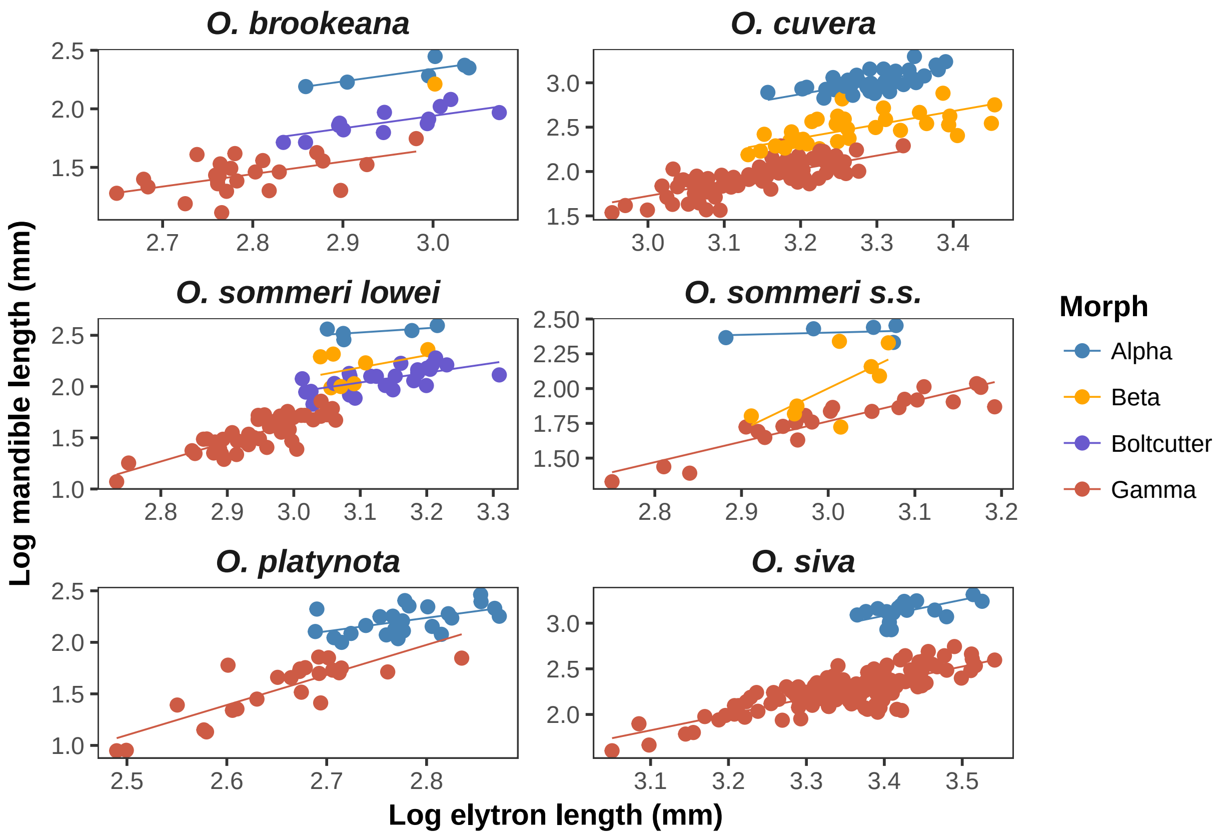


```
# ggsave('Figures/allometry_plot2.png', height = 25, width =
# 20, dpi = 300, units = 'cm')
```
